# WAO International Scientific Conference (WISC 2016) Abstracts: Jerusalem, Israel. 6–9 December 2016

**DOI:** 10.1186/s40413-017-0155-2

**Published:** 2017-06-20

**Authors:** Jun Bao, Yi-Hui Wang, Quan-Hua Liu, Yi-Xiao Bao, Nurit Azouz, Julie Caldwell, Leanne Ray, Mark Rochman, Melissa Mingler, Matthew Eilerman, Ting Wen, Jocelyn Biagini Myers, Gurjit Khurana Hershey, Leah Kottyan, Lisa Martin, Rothenberg Marc, Victor Gonzalez-Uribe, Jaime Del Rio-Chivardi, Blanca Del Rio-Navarro, Hongfei Lou, Siyuan Ma, Yan Zhao, Feifei Cao, Fei He, Zhongyan Liu, Chengshuo Wang, Claus Bachert, Luo Zhang, Elissa Abrams, Allan Becker, Amit Kandhare, Subhash Bodhankar, Nicole Grossman, Gheorghe Doros, Francine Laden, Anne Fuhlbrigge, Michael Wechsler, Wilson Pace, Barbara Yawn, Elliot Israel, Junehyuk Lee, Frederick Adler, Peter Kim, Yung Feng Huang, Ying Yao Chen, Chiun Yen Pan, Herng Sheng Lee, Michael Khalemsky, David G. Schwartz, Pavel Kolkhir, Dmitry Pogorelov, Nikolay Kochergin, Hirsh Komarow, Michael Young, Robin Eisch, Linda Scott, Dean Metcalfe, Alexander Singer, Andrew Wakeman, Thomas Gerstner, Elissa Abrams, Woo-Jung Song, Ji-Su Shim, Ha-Kyeong Won, Sung-Yoon Kang, Kyoung-Hee Sohn, Byung-Keun Kim, Eun-Jung Jo, Min-Hye Kim, Sang-Heon Kim, Heung-Woo Park, Sun-Sin Kim, Yoon-Seok Chang, Alyn H. Morice, Byung-Jae Lee, Sang-Heon Cho, Kyung-Up Min, Maria Assunta Boscolo, Giulio Brivio, Sergio Bosisio, Nicoletta Manzocchi, Edoardo Pulixi, Giulia Grignani, Eloisia D’Andrea, Massimo Ricci, Elena Passini, Maurizio Italia, Marilyn Urrutia-Pereira, Stefani Fagundes, Vinicius Jardim Oliano, Dirceu Solé, Sadia Benzaquen, Alejandro Aragaki, Ricardo Balestra, Dawn Harden, Danielle Caudell-Stamper, Gilbert Glady, Mark Holbreich, Nataliya Lyakhovska, Igor Kaidashev, Jaromir Bystron, Beata Hutyrova, Galina Balakirski, Luk Vanstreels, Gerda Wurpts, Hans F. Merk, Jens Malte Baron, Johanna Plange, Hans-Peter Rihs, Monika Raulf, Stefani Roeseler, Alberto Tolcachier, Armando Chamorro, Ruth Otero, Joel Brooks, Michael Hess, Jared Benz, Joseph MacDonald, Joel Brooks, Jared Benz, Usma Chatha, Dale Lent, Şükran Köse, Bengü Gireniz Tatar, Gülgün Akkoçlu, İbrahim Çukurova, İlker Ödemiş, Ayşin Kılınç Toker, Abdullahi Hasssan, Abdulrazaq Abdullahi Gobir, Cheol-Woo Kim, Young Hwa Choi, Jeong Hye Lee, Rae Jeong Cho, Yu Ran Nam, Joo Hyun Nam, Woo Kyung Kim, Ivana Filipovic, Zorica Zivkovic, Djordje Filipovic, Dana Shik, Andrew Smith, Wang Yui Hsi, Stuart Friedman, Yonatan Gizaw, Rima Bakhda, Kumail Mohammed, Richard Wasserman, Angela Hague, Deanna Pence, Joanna Rolen, Robert Sugerman, Stacy Silvers, Qurat Kamili, Nadezhda Knauer, Alexandr Zazernyi, Elena Blinova, Daria Demina, Vladimir Kozlov, Komal Agrawal, Sagar Kale, Naveen Arora, Volha Vasilkova, Tatiana Mokhort, William Silvers, Rachel Eisenberg, Rushita Mehta, Arye Rubinstein, Antony Aston, Paul Turner, Monica Ruiz-Garcia, Robert Boyle, Simon Brown, Yael Dinur Schejter, Adi Ovadia, Vy Kim, Brenda Reid, Chaim Roifman, Lana Rosenfield, Ernie Avilla, Laurie Harada, Marilyn Allen, Susan Waserman, Ho Joo Yoon, Gun Woo Koo, Suk-Il Chang, Hye-Ran Yoon, Dong Won Park, Tai Sun Park, Ji-Yong Moon, Sang-Heon Kim, Tae Hyung Kim, Jang Won Sohn, Dong Ho Shin, Tsici Jorjoliani, Lia Jorjoliani, Nino Adamia, Nona katamadze, Deepika Ramachandra, Liana Jorjoliani, Rusudan Karseladze, Lali Saginadze, Nino Adamia, Natalia Chkuaseli, Anna Dolgova, Olga Stukolova, Anna Sudina, Anna Cherkashina, German Shipulin, Giulio Brivio, Maria Assunta Boscolo, Richard Rosenthal, Harvey Howe, Paul Knause, Rony Greemberg, Jean Jacques De Bruycker, Isabel Fernandez, Françoise Le Deist, Elie Haddad, Yeong Ho Rha, Kyung Suk Lee, Sun Hee Choi, Herman Tam, Estelle Simons, Elinor Simons, Maria Golebiowska-Wawrzyniak, Katarzyna Markiewicz, Yoram Faitelson, Miguel Stein, Avigdor Mandelberg, Ilan Dalal, Michael Levin, Lelani Hobane, Wisdom Basera, Maresa Botha, Claudia Gray, Heather Zar, Biserka Jovkovska Kjaeva, Zoran Arsovski, Vesna Grivcheva-Panovska, Adeyinka Odebode, Adedotun Adekunle, Peter Adeonipekun, Ebenezer Farombi, Nadezhda Camacho-Ordoñez, Alejandrina Josefina Martinez-Vázquez, María de la Luz H. García-Cruz, Qi Tan, Rui Min, Guan-qun Dai, Wei-Ping Xie, Huang Mao, Hong Wang, Rakesh Yadav, Sneha Singh, Divya Yadav, Ekaterina Khaleva, Henry T. Bahnson, Amber Franz, Lene Heise Garvey, Nicola Jay, Rubaiyat Haque, Adam Fox, Gideon Lack, George du Toit, Snezana Radic, Branislava Milenkovic, Ana Neskovic, Ljiljana Danojevic, Liat Nachshon, Michael Goldberg, Michael Levy, Yitzhak Katz, Arnon Elizur, Cristine Rosario, Juliana Kasper, Herbeto Chong-Neto, Carlos Riedi, Nelson Rosario, Arnon Elizur, Michael B. Levy, Ronly Har-Even, Liat Nachshon, Mor Carmel, Michael R. Goldberg, Maia Kherkheulidze, Nani Kavlashvili, Eka Kandelaki, Nino Adamai, Maia Kherkheulidze, Lia Jorjoliani, Irma Ubiria, Andrea Burke, Ernie Avilla, Monika Kastner, Susan Waserman, Denica Zheleva, Razvigor Darlenski, Konstantinos Bozinakis, Anastasios Kriebardis, Sofia Styliara, Aikaterini Karastathi, Nikolaos Farmakas, Maria Luiza Kraft Kohler Ribeiro, Ana Carolina Barcellos, Hannah Gabriele Ferreira Silva, Luís Henrique Mattei Carletto, Marcela Carolina Bet, Nathalia Zorze Rossetto, Nelson Augusto Rosario, Herberto Jose Chong-Neto, Fernanda Valença, Marina Novaes, Mariana Gomes, Carla Seifert, Alfredo Neto, Flavia Loyola, José Rios, Tatiana Silva, Aline Neves, Marina Novaes, Fernanda Valença, Mariana Gomes, Alfredo Neto, Flavia Loyola, José Rios, Oznur Abadoglu, Bilun Gemicioglu, Hasan Bayram, Arif Cimrin, Levent Akyildiz, Aykut Cilli, Hakan Gunen, Tevfik Ozlu, Mecit Suerdem, Esra Uzaslan, Zeynep Misirligil, Branislava Milenkovic, Snezana Radic, Snezana Ristic-Stojanovic, A. Milicevic, A. Milenkovic, Jelena Cvejic, Jelena Jankovic, Sanja Dimic-Janjic, Natasa Djurdjevic, Vladyslava Barzylovych, Tetiana Umanets, Anastasia Barzylovych, Karyn Winkler, Jessica Margarinos, Dylan Martin, Maja Nowakowski, Rauno Joks, Miguel Stein, Tsili Zangen, Olga Bernadsky, Mona Boaz, Gratiana Hermann, Yoram Faitelson, Ilan Dalal, Rachel Aviv, Olga Kuperboim, Larisa Ramichanov, Efrat Broide, Raanan Shamir, Noam Zevit, Ron Shaoul, Alex Fich, Arie Levine, Adi Ovadia, Yael Dinur Schejter, Chaim Roifman, Isaac Melamed, Roopesh Singh Gangwar, Yael Minai-Fleminger, Mansour Seaf, Amichai Gutgold, Aarti Shikotra, Anoop Chauhan, Stephen Holgate, Peter Bradding, Peter Howarth, Ron Eliashar, Neville Berkman, Francesca Levi-Schaffer, Sung-il Woo, Betul Celik, Tangul Bulut, Arzu Didem Yalcin, Betul Celik, Arzu Didem Yalcin, Tangul Bulut, Luiz Querino Caldas, Ronit Confino-Cohen, Yossi Rosman, Arnon Goldberg, Oded Breuer, Roopesh Singh, Mansour Seaf, Ahlam Barhoum, Eitan Kerem, Francesca Levi-Schaffer, Tatiana Slavyanskaya, Revaz Sepiashvili, Elena A. Blinova, Ekaterina A. Pashkina, Marina I. Leonova, Vera M. Nepomnyaschikh, Darya V. Demina, Vladimir A. Kozlov, Revital Shamri, Kristen M. Young, Peter F. Weller, Rodolfo de Paula Vieira, Manoel Carneiro Oliveira-Junior, Nilsa Regina Damasceno-Rodrigues, Fernanda Magalhães Arantes-Costa, Milton Arruda Martins, Ana Paula Ligeiro Oliveira, Alfred Bernard, Antonia Sardella, Catherine Voisin, Simon Royce, Hamish Philpott, Sanjay Nandurkar, Francis Thien, Peter Gibson, Rodolfo Bianchini, Franziska Roth-Walter, Anna Ohradanova-Repic, Gerlinde Hofstetter, Ina Herrmann, Maria Isabel Carvalho, Karin Hufnagl, Erika Bajna, Georg Roth, Hannes Stockinger, Erika Jensen-Jarolim, Meital Almog, Aharon Kessel, Larisa Apov, Carlos Sanchez Salguero, Alvaro Sanchez Chacon, Abbos Nazarov, Shaxbos Ergashev, Irina Nesterova, Svetlana Kovaleva, Galina Chudilova, Ludmila Lomtatidze, Sarah De Schryver, Alizee Dery, Ann Clarke, Kari Nadeau, Laurie Harada, Kimberly Weatherall, Celia Greenwood, Denise Daley, Yuka Asai, Moshe Ben-Shoshan, Irina Balmasova, Elena Malova, Karin Hufnagl, Stefanie Wagner, Luis F. Pacios, Michael Wallner, Markus Wiederstein, Gerlinde Hofstetter, Franziska Roth-Walter, Erika Jensen-Jarolim, Anna E. Tevs, Ekaterina A. Pashkina, Marina I. Leonova, Vera M. Nepomnyaschikh, Darya V. Demina, Vladimir A. Kozlov, Elena A. Blinova, Nataly Tataurshchikova, Baigalmaa Sangidorj, Anna Ronzhina, Revaz Sepiashvili, Tatiana Slavyanskaya, Manana Chikhladze, Oliver F. Wirz, Willem van de Veen, David Mirer, Hideaki Morita, Can Altunbulakli, Sebastian L. Johnston, Nicholas Glanville, Nikolaos G. Papadopoulos, Cezmi A. Akdis, Mübeccel Akdis, Avner Reshef, Marc Riedl, Vesna Grivcheva Panovska, Dumitru Moldovan, James Baker, William H. Yang, Sladjana Andrejevic, Richard F. Lockey, Roman Hakl, Shmuel Kivity, Luca Bellizzi, Joseph R. Harper, Anurag Relan, Marco Cicardi

**Affiliations:** 1 Xin Hua Hospital, Shanghai Jiao Tong University School of Medicine Pediatric Respirology, Shanghai, China; 2 Cincinnati Childrens Hospital Medical Center, Division of Allergy and Immunology, Cincinnati, OH USA; 3 Cincinnati Childrens Hospital Medical Center, Division of Asthma Research, Cincinnati, OH USA; 4 Cincinnati Childrens Hospital Medical Center, Division of Human Genetics, Cincinnati, OH USA; 5 Universidad La Salle Facultad Mexicana de Medicina, Mexico City, Mexico; 6 Hospital Infantil de Mexico Federico Gomez Pediatric Allergy & Clinical Immunology, Mexico City, Mexico; 7 Beijing TongRen Hospital, Department of Otolaryngology Head and Neck Surgery, Beijing, China; 8 Beijing Institute of Otolaryngology, Beijing Key Laboratory of Nasal Diseases, Beijing, China; 9 Beijing TongRen Hospital, Department of Allergy, Beijing, China; 10 Ghent University Hospital, Upper Airways Research Laboratory, Ghent, Belgium; 11 University of Manitoba Section of Allergy and Immunology, Department of Paediatrics and Child Health, Winnipeg, Canada; 12 India Department of Pharmacology, Pune, Poona College of Pharmacy, Bharati Vidyapeeth Deemed University, Department of Pharmacology, Erandwane, Paud Road, Pune, 411038 Maharashtra India; 13 Brigham and Women Pulmonary Critical Care, Boston, MA USA; 14 Harvard University, Harvard Clinical Research Institute, Boston, MA USA; 15 Harvard TH Chan School of Public Health, Environmental Health, Boston, MA USA; 16 Brigham and Women’s Hospital, Pulmonary Critical Care, Boston, MA USA; 17 National Jewish Health, Pulmonary Critical Care, Denver, CO USA; 18 American Academy of Family Physicians, Family Medicine, Denver, CO USA; 19 University of Minnesota, Family and Community Health, Minneapolis, Minnesota, MN USA; 20 Soonchynhyang University Respiratory-Allergy, Bucheon, South Korea; 21 Utah University, Biology, Salt Lake City, UT USA; 22 Sydney University, Mathematics, Sydney, Australia; 23 Kaohsiung Veterans General Hospital, Pediatrics, Kaohsiung, Taiwan; 24 Kaohsiung Veterans General Hospital, Cardiac Surgery, Kaohsiung, Taiwan; 25 Kaohsiung Veterans General Hospital, Pathology, Kaohsiung, Taiwan; 26 Bar-Ilan University, Graduate School of Business Administration, Ramat Gan, Israel; 27 Sechenov First Moscow State Medical University, Dermatology and Venereology, Moscow, Russia; 28 National Institute of Allergy and Infectious Diseases, National Institutes of Health, Mast Cell Biology Section, Laboratory of Allergic Diseases, Bethesda, MD USA; 29 Leidos Biomedical Research, Inc., Frederick National Laboratory for Cancer Research Clinical Research Directorate/Clinical Monitoring Research Program, Frederick, MD USA; 30 University of Manitoba, Family Medicine, Winnipeg, Canada; 31 UCD Medical School, Dublin, Ireland; 32 University of Manitoba, Paediatric Allergy, Winnipeg, Canada; 33 Seoul National University, College of Medicine, Department of Internal Medicine, Seoul, South Korea; 34 Pusan National University, College of Medicine, Department of Internal Medicine, Busan, South Korea; 35 Ewha Woman’s University, School of Medicine, Department of Internal Medicine, Seoul, South Korea; 36 Hanyang University, College of Medicine, Department of Internal Medicine, Seoul, South Korea; 37 Hull York Medical School, Castle Hill Hospital, University of Hull, Cottingham Centre for Cardiovascular and Metabolic Research, Cottingham, United Kingdom; 38 Sungkyunkwan University, School of Medicine, Department of Medicine, Seoul, South Korea; 39 Merate Hospita,l Allergology, Osnago, Italy; 40 Federal University of Pampas (Unipampa), Children Pediatric, Uruguaiana, Brazil; 41 Federal University of Pampas (Unipampa), Pediatrics, Uruguaiana, Brazil; 42 University of Campanha Region (URCAMP), Pediatrics, Uruguaiana, Brazil; 43 Federal University of São Paulo Allergy, Clinical Immunology and Rheumatology, São Paulo, Brazil; 44 University of Cincinnati, Pulmonary, Cincinnati, OH USA; 45 EBMA clinical research, Colmar, France; 46 Allergy Consultants, Indianapolis, IN USA; 47 Supreme State educational institution of Ukraine “Ukrainian Medical Stomatological Academy”, Ministry of Health, Poltava, Ukraine; 48 University Hospital Allergology and Clin. Immunology, Olomouc, Czech Republic; 49 University Hospital of RWTH Aachen, Department of Dermatology and Allergology, Aachen, Germany; 50 Ruhr-University Bochum, Institute for Prevention and Occupational Medicine of the German Social Accident Insurance, Bochum, Germany; 51 Durand Hospital, Allergy, Buenos Aires, Argentina; 52 Soluciones Ambientales CIH, Buenos Aires, Argentina; 53 Yale University, School of Medicine, Allergy and Immunology, New Haven, CT USA; 54 Heart of Lancaster Regional Medical Center, Internal Medicine, Lititz, PA USA; 55 Yale University, School of Medicine, Allergy and Immunology, New Haven, CT USA; 56 Heart of Lancaster Regional Medical Center Internal Medicine, Lititz, PA USA; 57 Tepecik Training and Research Hospital, Infectious Diseases and Clinical Microbiology and Immunology, Izmir, Turkey; 58 Tepecik Training and Research Hospital, Infectious Diseases and Clinical Microbiology, Izmir, Turkey; 59 Tepecik Training and Research Hospital, Department of Otorhinolaryngology, Izmir, Turkey; 60 Federal medical Centre Scientific Research, Keffi, Nasarawa state Nigeria; 61 Faculty of Medicine, Ahmadu Bello University Community Medicine, Zaria, Kaduna State Nigeria; 62 Inha University School of Medicine, Incheon, South Korea; 63 Ajou University School of Medicine, Suwon, South Korea; 64 Dongguk University, Physiology, Goyang-Korea, South Korea; 65 Faculty of Medical Science Kragujevac-Immunology, Kragujevac, Serbia; 66 Children’s Hospital for Lung Diseases and Tuberculosis- Medical Center Dr Dragisa Misovic-Pediatric, Belgrade, Serbia; 67 Institution for Emergency Medical Care Anesthesiology, Belgrade, Serbia; 68 CCHMC Allergy and Immunology, Cincinnati, OH USA; 69 Schmidt College of Medicine Florida Atlantic University Clinical Integrative Medicine, Boca Raton, FL USA; 70 Boca Raton Regional Hospital Medicine, Boca Raton, FL USA; 71 Medical City Dallas Hospital Pediatrics, Dallas, TX USA; 72 Medical City Dallas Hospital Medicine, Dallas, TX USA; 73 Research Institute of Fundamental and Clinical Immunology, Laboratory of Clinical Immunopathology, Novosibirsk, Russia; 74 Novosibirsk State Medical University Pediatric Faculty, Novosibirsk, Russia; 75 Research Institute of Fundamental and Clinical Immunology, Allergology Department of the Clinic of Immunopathology, Novosibirsk, Russia; 76 CSIR-Institute of Genomics and Integrative Biology Allergy and Immunology section, Delhi, India; 77 Gomel State Medical University Endocrinology, Gomel, Belarus; 78 Belarusian State Medical University Endocrinology, Minsk, Belarus; 79 University of Colorado School of Medicine Medicine GWV, Aurora, CO USA; 80 Montefiore Medical Center Allergy and Immunology, Bronx, New York USA; 81 Imperial College London Paediatric Allergy & Immunology, London, United Kingdom; 82 University of Western Australia Emergency Medicine, Perth, Australia; 83 Hospital for Sick Children, Allergy and Immunology, Toronto, Canada; 84 McMaster University Division of Clinical Immunology and Allergy, Hamilton, Canada; 85 McMaster University Department of Medicine, Hamilton, Canada; 86 Food Allergy Canada, Toronto, Canada; 87 Hanyang University College of Medicine Department of Internal Medicine, Seoul, South Korea; 88 Sungae General Hospital Department of Internal Medicine, Seoul, South Korea; 89 Duksung Women’s University College of Pharmacy, Seoul, South Korea; 90 D.aghmashenebeli University Pediatric, Tbilisi, Georgia; 91 I.Javakhishvili Tbilisi Steit University Pediatric, Tbilisi, Georgia; 92 Tbilisi State Medical University Pediatric, Tbilisi, Georgia; 93 BMCRI Immunology, Bangalore, India; 94 Tbilisi State Medical University, Department of Pediatrics, Tbilisi, Georgia; 95 Central Research Institute for Epidemiology, Federal Service on Consumers’ Rights Protection and Human Well-Being Surveillance Molecular Diagnostics and Epidemiology, Moscow, Russia; 96 Ospedale Civile San Leopoldo Mandic Allergology Merate, Lecco, Italy; 97 Johns Hopkins School of Medicine Medicine, Baltimore, Maryland USA; 98 Inova Fairfax Hospital, Fairfax, VA USA; 99 Self, Vienna, VA USA; 100 Self, Arlington, VA USA; 101 CHU Sainte-Justine, Allergy & Clinical Immunology, Montreal, Canada; 102 Kyung Hee University School of Medicine Pediatrics, Seoul, South Korea; 103 CHA Unitersity Scool of Medicine Pediatircs, Bungdang, South Korea; 104 Department of Pediatrics and Child Health, University of Manitoba, Winnipeg, Canada; 105 Institute of Mother and Child Clinical Immunology, Warszawa, Poland; 106 Sackler Faculty of Medicine, Tel Aviv University, Tel Aviv, Israel Allergy and Immunology Unit, Wolfson Medical Center, Holon, Israel; 107 Sackler Faculty of Medicine, Tel Aviv University, Tel Aviv, Israel The Pulmonary Unit, Department of Pediatrics, Wolfson Medical Center, Holon, Israel; 108 University of Cape Town, Paediatric Allergology, Cape Town, South Africa; 109 University of Cape Town, Public health, Cape Town, South Africa; 110 University of Cape Town, Paediatrics, Cape Town, South Africa; 111 Clinic of Pulmonology and Allergy, Skopje, Macedonia; 112 Clinic of Pulmonology and Allergy Functional Diagnostic, Skopje, Macedonia; 113 School of Medicine, University St. Cyril and Methodius University, Clinic of Dermatology, Skopje, Macedonia; 114 University of Lagos, Botany, Akoka, Nigeria; 115 University of Ibadan, Biochemistry, Ibadan, Nigeria; 116 Londres Clinic Allergy and Clinical Immunology, Mexico City, Mexico; 117 Dalinde Medical Center Allergy and Clinical Immunology, Mexico City, Mexico; 118 National Institute of Respiratory Diseases Allergy and Immunogenetic Research Department, Mexico City, Mexico; 119 The First Affiliated Hospital of Nanjing Medical University, Department of Respiratory and Critical Care Medicine, Nanjing, China; 120 Banasthali University, Pharmaceutical Chemistry, Banasthali, India; 121 Guy’s and St Thomas’ NHS Foundation Trust, Children’s Drug Allergy Clinic, London, UK; 122 Benaroya Research Institute, Seattle, WA USA; 123 Seattle Children’s Hospital, Seattle, WA USA; 124 University Hospital Gentofte, Danish Anaesthesia Allergy Centre, Allergy Clinic, Department of Dermato-Allergology, Gentofte, Denmark; 125 Sheffield Children, Sheffield, UK; 126 Guy’s and St Thomas’ NHS Foundation Trust, Adult’s Drug Allergy Clinic, London, UK; 127 Clinical Hospital Center Dr Dragisa Misovic Dedinje Children’s Hospital for Respiratory Diseases and TB, Belgrade, Serbia; 128 Clinical Centre of Serbia,Faculty of Medicine, University of Belgrade Clinic for Pulmonary Diseases, Belgrade, Serbia; 129 Gerontology Center Belzanijska kosa Medical practice, Belgrade, Serbia; 130 Assaf Harofeh Medical Center Allergy and Immunology, Zerifin, Israel; 131 Federal university of Parana Allergy and Immunology, Curitiba, Brazil; 132 Assaf Harofeh Medical Center Allergy and Immunology, Beer Yaakov, Israel; 133 State Medical Universitym, Pediatrics, Tbilisi, Georgia; 134 Tbilisi State Medical University, Department of Pediatrics, Tbilisi, Georgia; 135 McMaster University, Clinical Immunology & Allergy, Hamilton, Canada; 136 McMaster University, Medicine, Hamilton, Canada; 137 North York General Hospital, Knowledge Translation and Implementation, Toronto, Canada; 138 Tokuda Hospital Sofia, Dermatology and venereology, Sofia, Bulgaria; 139 General Hospital of Nikea-piraeus, Allergy and immunology, Nikea-Piraeus, Greece; 140 Tei Tei, Aigaleo, Greece; 141 Federal University of Paraná, Healthy Community, Curitiba, Brazil; 142 Federal University of Paraná, Pediatrics, Curitiba, Brazil; 143 Policlinica Geral do Rio de Janeiro, Allergy, Rio de Janeiro, Brazil; 144 Policlínica Geral do Rio de Janeiro, Allergy, Rio de Janeiro, Brazil; 145 Cumhuriyet University, Faculty of Medicine, Department of Pulmonary Diseases, Sivas, Turkey; 146 Istanbul University, Cerrahpasa Faculty of Medicine, Department of Pulmonary Diseases, Istanbul, Turkey; 147 Gaziantep University, Faculty of Medicine, Department of Pulmonary Diseases, Gaziantep, Turkey; 148 Dokuz Eylul University, Faculty of Medicine, Department of Pulmonary Diseases, Izmir, Turkey; 149 Mardin Medical Park Hospital, Department of Pulmonary Diseases, Mardin, Turkey; 150 Akdeniz University, Faculty of Medicine, Department of Pulmonary Diseases, Antalya, Turkey; 151 Sureyyapasa Pulmonary Diseases Hospital and Research Centre, Department of Pulmonary Diseases, Istanbul, Turkey; 152 Karadeniz Technical University, Faculty of Medicine, Department of Pulmonary Diseases, Trabzon, Turkey; 153 Selcuk University, Faculty of Medicine, Department of Pulmonary Diseases, Konya, Turkey; 154 Uludag University, Faculty of Medicine, Department of Pulmonary Diseases, Bursa, Turkey; 155 Ankara University, Faculty of Medicine, Department of Pulmonary Diseases, Ankara, Turkey; 156 Clinical Centre of Serbia, Clinic for Pulmonary Diseases, Belgrade, Serbia; 157 Clinical Hospital Center “Dr Dragisa Misovic-Dedinje”, Children’s Hospital for Respiratory Diseases and TB, Belgrade, Serbia; 158 Institute Student’s Health Care Institute, Belgrade, Serbia; 159 Bohomolets National Medical Univercity, Pediatrician departemen, Kiev, Ukraine; 160 Institute of Pediatrics, Obstetrics and Gynecology, Drug Allergy Center, Kiev, Ukraine; 161 Oberig clinic, Pediatrician department, Kiev, Ukraine; 162 State University Of New York Downstate Medical Center, Center for Allergy and Asthma Research, Brooklyn, New York, NY USA; 163 State University Of New York Downstate Medical Center, Department of Pediatrics, Brooklyn, New York, NY USA; 164 State University Of New York Downstate Medical Center, College of Medicine, Brooklyn, New York, NY USA; 165 State University Of New York Downstate Medical Center, Department of Pathology, Brooklyn, New York, NY USA; 166 State University Of New York Downstate Medical Center, Department of Medicine, Brooklyn, New York, NY USA; 167 E. Wolfson Medical Center, Allergy/Immunology Unit, Holon, Israel; 168 E. Wolfson Medical Center, Pediatric Gastroenterology, Holon, Israel; 169 E. Wolfson Medical Center, Pathology, Holon, Israel; 170 E. Wolfson Medical Center, Biostatistics, Holon, Israel; 171 Assaf Harofeh, Pathology, Zerifin, Israel; 172 Assaf Harofeh Medical Center, Pediatric Gastroenterology, Zerifin, Israel; 173 Schneider Children, Pediatric Gastroenterology, Petach Tikva, Israel; 174 Rambam Medical Center, Pediatric Gastroenterology, Haifa, Israel; 175 Soroka Medical Center, Gastroenterology, Beer Sheva, Israel; 176 Hospital for sick children, Immunology and Allergy, Toronto, Canada; 177 ImmunoE Immunology, Centennial, CO USA; 178 The Hebrew University of Jerusalem Institute For Drug Research, Portsmouth, Israel; 179 University of Leicester Institute for Lung Health, Leicester, UK; 180 Portsmouth Hospitals NHS Trust Respiratory and General Medicine, Portsmouth, UK; 181 Southampton General Hospital NIHR Respiratory Biomedical Research Unit, Southampton, UK; 182 Hadassah Hebrew University Medical Center Department of Otolaryngology/Head and Neck Surgery, Jerusalem, Israel; 183 Hadassah Hebrew University Medical Center Institute of Pulmonary Medicine, Jerusalem, Israel; 184 Chung-buk National University Hospital Department of Pediatrics, Choeng-ju, South Korea; 185 Antalya Training Hospital Pathology, Antalya, Turkey; 186 Antalya Training Hospital Allergy and Immunology, Antalya, Turkey; 187 Antalya Training Hospital Pathology, Antalya, Turkey; 188 Antalya Training Hospital Allergy and Immunology, Antalya, Turkey; 189 Federal Fluminense University Pathology, Niterói, Brazil; 190 Meir Medical Center Allergy and Clinical Immunology Unit, Kefar Saba, Israel; 191 Hadassah Hebrew University Medical Center Pediatric Pulmonology, Jerusalem, Israel; 192 The Hebrew University of Jerusalem Department of Pharmacology and Experimental Therapeutics, Institute for Drug Research, Faculty of Medicine, Jerusalem, Israel; 193 People’s Friendship University of Russia Allergy & Immunology, Moscow, Russia; 194 RIFCI laboratory of clinical immunopathology, Novosibirsk, Russia; 195 Clinic of Immunopathology RIFCI allergological department, Novosibirsk, Russia; 196 Hebrew University of Jerusalem School of Pharmacy, Jerusalem, Israel; 197 Beth Israel Deaconess Medical Center, Harvard Medical School Department of Medicine, Boston, MA USA; 198 Nove de Julho University Laboratory of Pulmonary and Exercise Immunology, Sao José dos Campos, Brazil; 199 School of Medicine, University of Sao Paulo Pathology (LIM 59), Sao Paulo, Brazil; 200 School of Medicine, University of Sao Paulo Clinical Medicine (LIM 20), Sao Paulo, Brazil; 201 Catholic University of Louvain Toxicology, Brussels, Belgium; 202 Monash University Medicine, CCS, Melbourne, Australia; 203 Monash University Gastroenterology, CCS, Melbourne, Australia; 204 Monash University Gastroenterology, ECS, Melbourne, Australia; 205 Monash University Respiratory Medicine, Melbourne, Australia; 206 The Interuniversity Messerli Research Institute; University of Veterinary Medicine; Medical University of Vienna; University of Vienna Department of Comparative Medicine, Vienna, Austria; 207 Center for Pathophysiology, Infectiology and Immunology, Medical University of Vienna Institute for Hygiene and Applied Immunology, Vienna, Austria; 208 University of Veterinary Medicine Clinical Department of Small Animal Internal Medicine, Vienna, Austria; 209 University of Tràs-os-Montes and Alto Douro Department of Veterinary Science, Vila Real, Portugal; 210 Center of Pathophysiology, Infectiology and Immunology, Medical University Vienna Department of Pathophysiology and Allergy Research, Vienna, Austria; 211 General Intensive Care and Pain Medicine, Medical University Vienna Department of Anesthesiology, Vienna, Austria; 212 Bnai Zion Medical Center Division of Allergy & Clinical Immunology, Haifa, Israel; 213 Bnai Zion Medical Center Division of Allergy & Clinical Immunology, Haifa, Israel; 214 University Hospital Puerto Real Pediatric, Puerto Real (Cadiz), Spain; 215 Tashkent Medical Academy Allergology, Tashkent, Uzbekistan; 216 Medstar Biology, Qarshi, Uzbekistan; 217 The Peoples’ Friendship University of Russia, Department of Allergology and Immunology, Moscow, Russia; 218 McGill University Pediatric Allergy and Immunology, Montreal, Canada; 219 McGill University Clinical Epidemiology, Montreal, Canada; 220 University of Calgary Rheumatology, Calgary, Canada; 221 Stanford University Pediatric Allergy, Immunology and Rheumatology, Palo Alto, CA USA; 222 Anaphylaxis Canada, Toronto, Canada; 223 Multiple Births Canada, Lefroy, Canada; 224 McGill University Oncology, Epidemiology, Biostatistics, Occupational Health and Human Genetics, Montreal, Canada; 225 University of British Columbia Medicine, Vancouver, Canada; 226 Queen’s University Dermatology, Kingston, Canada; 227 Peoples’ Friendship University of Russia Allergology & Immunology, Moscow, Russia; 228 The interuniversity Messerli Research Institute of the University of Veterinary Medicine, the Medical University and the University of Vienna Comparative Medicine, Vienna, Austria; 229 ETSI Montes, Technical University of Madrid Department of Natural Systems and Resources, Madrid, Spain; 230 University of Salzburg Department of Molecular Biology, Salzburg, Austria; 231 Center of Pathophysiology, Infectiology and Immunology, Medical University Vienna Department of Pathophysiology and Allergy Research, Vienna, Austria; 232 Clinic of Immunopathology RIFCI Allergological Department, Novosibirsk, Russia; 233 RIFCI Laboratory of Clinical Immunopathology, Novosibirsk, Russia; 234 Peoples Allergology and Immunology, Moscow, Russia; 235 Peoples’ Friendship University of Russia Allergy and Immunology, Moscow, Russia; 236 National Institute of Allergology, Asthma and Clinical Immunology of Georgian Academy of Sciences Immunology and Allergy, Tskhaltubo, Georgia; 237 University of Zurich Swiss Institute of Allergy and Asthma Research (SIAF), Davos Platz, Switzerland; 238 University of Tokyo National Research Institute for Child Health and Development, Tokyo, Japan; 239 Imperial College London Airway Disease Infection Section, National Heart and Lung Institute, London, United Kingdom; 240 University of Manchester Division of Infection, Immunity and Respiratory Medicine, Manchester, United Kingdom; 241 University of Tel Aviv Sheba Medical Center, Tel-Hashomer, Israel; 242 University of California, San Diego Department of Medicine, La Jolla, CA USA; 243 Medical University of Skopje PHU Clinic for Dermatology, Skopje, Macedonia; 244 Mures County Hospital University of Medicine and Pharmacy, Tirgu, Mures Romania; 245 Baker Allergy Asthma Dermatology Departments of Allergy and Asthma, Lake Oswego, CA USA; 246 Ottawa Allergy Research Corporation/University of Ottawa Medical School Division of General Internal Medicine, Department of Medicine, Ottawa, Canada; 247 Clinical Center of Serbia Clinical Center, Belgrade, Serbia; 248 University of South Florida Morsani College of Medicine Division of Allergy and Immunology, Department of Internal Medicine, Tampa, FL USA; 249 St. Anne’s University Medical Department, Brno, Czech Republic; 250 The Tel Aviv Medical Center, Sackler Medica School The Allergy and Immunology Unit, Tel Aviv, Israel; 251 Pharming Technologies BV Department of Clinical Research and Medical Affairs, Leiden, The Netherlands; 252 Salix Pharmaceuticals Department of Medical Affairs, Raleigh, NC USA; 253 Universita degli Studi di Milano Department of Internal Medicine, Milan, Italy

## A1 A four-loci interaction model: a new predictive tool for asthma in Chinese children 5 years and younger

### Jun Bao, Yi-Hui Wang, Quan-Hua Liu, Yi-Xiao Bao

#### Xin Hua Hospital, Shanghai Jiao Tong University School of Medicine, Pediatric Respirology, Shanghai, China

##### **Correspondence:** Jun Bao

**Background**

Asthma is the most common chronic respiratory disease in childhood. However, it is quite difficult to make a prompt diagnosis of asthma in a child 5 years and younger, partly due to a lack of objective diagnostic means. Our previous studies on susceptibility gene of asthma showed that a gene-gene interaction among 4 single nucleotide polymorphisms including IL13 rs20541 and IL4 rs2243250, ADRB2 rs1042713 and FcER1B rs569108 had a predictable role for asthma in wheezing children.

**Objective**

The study was aimed to further investigate the predictive effects of the four-loci interaction model in Chinese preschool children with asthma.

**Methods**

A total of 212 wheezing children aged 6 months to 5 years were enrolled and followed up for at least one year at Shanghai Xinhua Hospital between Dec 2014 and Mar 2016. Clinical data and lab findings of atopy were collected. All the children were divided into the high-risk group and the low-risk group according to genotypes of the four-loci interaction model. The differences of clinical features were compared between the two groups. The predictive effects for asthma were analyzed among asthma predictive index (API), 2015 Canadian Diagnostic Criteria for Asthma in Preschoolers and our four-loci interaction model.

**Results**

Of all the enrolled 212 children, 117 (55.2%) were assigned into the high-risk group and 95 (44.8%) were the low-risk group. Compared with the low-risk group, the high-risk group had more yearly episodes of wheeze and a higher level of blood eosinophilia. More children in the high-risk group presented with afebrile wheeze, eczema and positive food or aero allergens and had a history of tobacco exposure. If the asthma-predictive effect of positive API was considered as 1, the four-loci interaction model had a sensitivity of 77.2%, a specificity of 80.0% and an AUC area of 0.786 with a modest consistency (*P*=0.22, Kappa=0.49), while the Canadian criteria had a sensitivity of 97.8%, a specificity of 53.3% and an AUC area of 0.539 with a low consistency (*P*<0.01, Kappa=0.265).

**Conclusions**

The four-loci interaction model is associated with the phenotypes of wheezing in Chinese preschoolers. It has a consistent predictive effect with API for asthma and is more specific than the Canadian criteria in the diagnosis of asthma, which indicates that the four-loci interaction model may be developed as a new objective predictive tool for asthma in Chinese children 5 years and younger.

## A2 Loss of esophageal epithelial SPINK7 unleashes uncontrolled proteolytic activity, impaired epithelial barrier, defective differentiation and pro-inflammatory cytokine production

### Nurit Azouz^1^, Julie Caldwell^1^, Leanne Ray^1^, Mark Rochman^1^, Melissa Mingler^1^, Matthew Eilerman^1^, Ting Wen^1^, Jocelyn Biagini Myers^2^, Gurjit Khurana Hershey^2^, Leah Kottyan^3^, Lisa Martin ^3^, Rothenberg Marc^1^

#### ^1^Cincinnati Childrens Hospital Medical Center, Division of Allergy and Immunology, Cincinnati, OH, United States; ^2^Cincinnati Childrens Hospital Medical Center, Division of Asthma Research, Cincinnati, OH, United States ; ^3^Cincinnati Childrens Hospital Medical Center, Division of Human Genetics, Cincinnati, OH, United States

##### **Correspondence:** Nurit Azouz

**Background**

Epithelial barrier impairment has been implicated in the development of allergic disease. However, the molecular mechanisms by which impaired epithelial barrier function induces Th2-type immune responses remain largely unknown. In this study, we examined the role of the serine peptidase inhibitor kazal type (SPINK)7 on epithelial barrier function and mucosal Th2-associated immune responses in the esophagus, with a focus on eosinophilic esophagitis (EoE), a chronic, antigen-driven, inflammatory allergic disease.

**Methods**

Primary human esophageal epithelial cells stably transduced with either control or SPINK7-directed shRNAs were cultured at the air-liquid interface (ALI) to induce squamous cell differentiation. The integrity of the epithelium was examined by functional assays complemented with histological and ultrastructural analyses as well as quantitation and localization of junctional proteins by immunofluorescence microscopy. The impact of SPINK7 on epithelial protease activity and global transcription was assessed. Furthermore, cytokine and chemokine secretion were analyzed following SPINK7 silencing. In vitro assays using recombinant proteins were conducted to identify direct targets of SPINK7. Protease activity of human esophageal tissue was measured, and receptor expression of esophageal tissue-derived eosinophils was quantified. Using a genetic approach, we assessed whether genetic variants in the SPINK7 gene were associated with EoE susceptibility.

**Results**

Loss of SPINK7 expression caused a defect in epithelial cell differentiation, reduced expression of barrier proteins including filaggrin, impaired epithelial barrier function, and unleashed the production of a set of pro-inflammatory mediators including IL-8, GM-CSF and CCL2. Mechanistically, recombinant SPINK7 directly inhibited urokinase plasminogen activator (uPA) and kallikrein (KLK)5 and translational studies revealed increased uPA activity in the esophagus of EoE patients and a marked decrease in expression of the uPA receptor by esophageal eosinophils. Genetic studies revealed epistasis between genetic variants in SPINK7 and PLAU (gene product, uPA) with atopy risk variants in ST2 and thymic stromal lymphopoietin (TSLP), respectively.

**Conclusions**

We propose that SPINK7 deficiency and uncontrolled protease activity serve a causative role in compromising the esophageal barrier. We suggest that SPINK7 represents a novel checkpoint in regulating innate immunity, and its deficiency, as occurs in EoE, induces pro-inflammatory and pro-allergic responses characterized by excessive cytokine production and epithelial barrier impairment, likely via a KLK- and uPA-dependent mechanism. Additionally, EoE disease susceptibility is influenced by genetic interactions between variants in this pathway (SPINK7 and PLAU) and cardinal atopy pathways (ST2 and TSLP).

## A3 Epidemiological transition of viruses associated with wheezing. The role of new virus

### Victor Gonzalez-Uribe^1^, Jaime Del Rio-Chivardi^2^, Blanca Del Rio-Navarro^2^

#### ^1^Universidad La Salle Facultad Mexicana de Medicina, Mexico City, Mexico ; ^2^Hospital Infantil de Mexico Federico Gomez Pediatric Allergy & Clinical Immunology, Mexico City, Mexico

##### **Correspondence:** Victor Gonzalez-Uribe

**Background**

Recurrent episodes of wheezing, coughing and respiratory distress in early life are associated with viral infections, being a common background in the history of patients with persistent wheezing & subsequent onset of asthma. This study allows to determine the prevalence of viruses associated with wheezing in preschool patients.

**Methods**

Analytical cohort study was conducted in preschool children who had airway infections with wheezing; in them, associated viruses were identified by nasopharyngeal and/or bronchial lavage with DNA extraction/viral RNA visualization and analysis by microarray. All patients underwent clinical history. Univariate statistical analysis was performed.

**Results**

A total of 714 patients were included for 1 year 6 months to 6 years of life, the mean age 3.7 years. In 342 patients (47.9%) a viral agent was not found, however, in patients with positive results, 38.4%, was isolated only one virus, 9.8% had coinfection by 2 viruses and 3.9% coinfection by 3 viruses. The viruses identified in order of frequency were: Rhinovirus 88 (12.3%), Respiratory Syncytial Virus (RSV) B 46 (6.4%), Parainfluenza3 31 (4.3%), VSRA 31 (4.3%), VSRA + Bocavirus 25 (3.5%), Influenza A H1N1 16 (2.2%), Enterovirus 14 (2%), VSRA + RSVB 12 (1.7%), Bocavirus 9 (1.3%) and the remaining 14.1% 10 other viruses.

**Conclusions**

While traditional viruses such as rhinovirus or RSV had greater participation, the presence of “nontraditional” viruses or coinfection by 2 or more virus requires asking questions about the participation of early immunological changes that favor the genesis of the disease.

## A4 Aeroallergen sensitizations in patients with allergic rhinitis in mainland China

### Hongfei Lou^1^, Siyuan Ma^1^, Yan Zhao^2^, Feifei Cao^3^, Fei He^2^, Zhongyan Liu^2^, Chengshuo Wang^1^, Claus Bachert^4^, Luo Zhang^1^

#### ^1^Beijing TongRen Hospital, Department of Otolaryngology Head and Neck Surgery, Beijing, China ; ^2^Beijing Institute of Otolaryngology, Beijing Key Laboratory of Nasal Diseases, Beijing, China ; ^3^Beijing TongRen Hospital, Department of Allergy, Beijing, China ; ^4^Ghent University Hospital, Upper Airways Research Laboratory, Ghent, Belgium

##### **Correspondence:** Hongfei Lou

**Background**

Sensitization pattern of allergic rhinitis (AR) was not clear within main land China.

Objective

Our aim was to investigate the pattern of sensitization in AR patients within mainland China and to define the minimal panel of skin prick test (SPT) allergens required to identify a patient as sensitized.

**Methods**

In the current patient-based study, 7148 patients suffering from AR symptoms in 28 provinces from 4 regions of mainland China underwent standardized SPT with 21 common aeroallergens. Conditional approach allowed to determine the allergens selection.

**Results**

Among the 7148 suspected AR patients, 6350 (88.8%) had at least one positive skin prick reaction. The prevalence of positive skin prick responses was 47.2% for Der f and 41.4% for Der p, respectively, which were the two most prevalent aeroallergens in main land China. The highest standardized sensitization rates for house dust mites were observed in south China (69.2% for Der f and 61.4% for Der p, respectively). However, in north-west China with cold arid and desert/steppe climate, the three most prevalent aeroallergens were mugwort, ragweed and dandelion (58.2%, 47.1% and 45.3%, respectively). Sensitization rates of outdoor aeroallergens were higher in moderate/severe AR compared to mild AR. Similar allergen sensitization pattern was observed in persistent AR compared to intermittent AR. Skin index of sensitization to outdoor allergens and mold allergens was found to be significantly correlated with symptom VAS score of AR, whereas severity of AR was not significantly correlated with skin index of reactivity of Derp, Der f and Alterneria. Sensitization rates of outdoor allergens and animal dander was higher in patients with AR and conjunctivitis compared to patients with only AR. Overall, eight allergens (Der f, mugwort, blatella, hazel, coosefoot, penicillium notatum, animal dander and Der p) allowed to identified more than 95% of sensitized subjects. However, differences were observed between regions, 4 allergens being sufficient for south China (Der f, blatella, dandelion and ragweed) as opposed to eight for middle China (same order in mainland China).

**Conclusions**

Dust mites were the most prevalent allergens in patients with AR in mainland China. There were significant differences in patterns of sensitizations in patients from different geographic areas. Eight allergens allowed the identification of the majority of sensitized subjects.

## A5 Oral food challenge outcomes in a tertiary care center

### Elissa Abrams, Allan Becker

#### University of Manitoba Section of Allergy and Immunology, Department of Paediatrics and Child Health, Winnipeg, Canada

##### **Correspondence:** Elissa Abrams

**Background**

Open oral food challenges are the usual clinical standard for food tolerance. However, clinicians continually search for better predictive approaches.

**Methods**

A retrospective chart review of all food challenges in children between 2008 and 2010 was performed.

**Results**

Using available predictive approaches 313 challenges were performed (105 peanut, 71 egg, 41 milk, 29 tree nut, 67 other) in children 8 months - 18 years old (median age 5.5 years). There were 104 failures (33%); 82 objective, and 22 subjective. Older children were more likely to fail an oral challenge than younger children (*p*=0.046). Rates of asthma (72% vs 47%) and atopic dermatitis (74% vs 60%) were significantly higher among those who failed oral challenges (*p*=0.0014 and *p*=0.03 respectively). Rates of food allergy (48% overall) and aeroallergen sensitization (81% overall) were not significantly different.

Risk of challenge failure was significantly different between food allergens (*p*=0.0013) with more failures noted for peanut (43%) than tree nut (34%), milk (22%), or egg (17%). Skin test size was significantly correlated with challenge failure for peanut only (*p*<0.0001). Specific IgE was significantly correlated with challenge failure for peanut only (*p*<0.0001). Food dose eliciting a reaction was significantly different (*p*=0.0177) between milk (3.0 mL), egg (2.0 mg), tree nut (0.75 mg), and peanut (0.30 mg). There was no significant correlation between initial reaction characteristics and reaction characteristics at oral challenge.

Among challenge failures, 20/104 (19%) met the criteria for anaphylaxis, with significantly more tree nut (70%) and peanut challenges (20%) causing anaphylaxis than milk (7%) or egg (5%) (*p*=0.0003). There were 5/8 (63%) failed cashew challenges, of which 4/5 (80%) required epinephrine; 3/5 (60%) of whom had no known prior exposure to cashew (skin tested 2°nut allergy). Cashew was significantly more likely to cause a reaction at oral challenge than the other tree nuts (24%; *p*=0.05) and other allergens (17%; *p*=0.0004).

**Conclusions**

Most challenge failures were to peanut and most severe reactions were to peanut and tree nut. Failures to peanut and tree nut occurred at low doses while most egg and milk reactions occurred at high doses. Those who failed a challenge had more atopic disease and were older. Cashew challenges commonly caused anaphylaxis even in children with no known prior exposure to cashew.

## A6 Effect of Morin in bleomycin-induced pulmonary fibrosis in rats: critical role of inflammatory, fibrotic and apoptotic biomarkers

### Amit Kandhare^1^, Subhash Bodhankar^1^

#### ^1^Poona College of Pharmacy, Bharati Vidyapeeth Deemed University, Department of Pharmacology, Erandwane, Paud Road, Pune 411038, Maharashtra, India

##### **Correspondence:** Amit Kandhare

**Background**

Idiopathic pulmonary fibrosis (IPF) is a chronic progressive multifactorial disease with limited successful treatment. Morin [2-(2,4-dihydroxyphenyl)-3,5,7-trihydroxy-4H-1-benzopyran-4-one] is a flavonoid possesses potent anti-inflammatory and anti-oxidant property.

**Objective**

To evaluate the effect of morin against bleomycin (BLM) induced pulmonary fibrosis by assessing various behavioral, biochemical, molecular and ultrastructural changes in the laboratory rats.

**Methods**

IPF was induced in male Sprague-Dawley rats by single intratracheal BLM (6 IU/kg) injection followed by Morin (10, 30 and 100 mg/kg, p.o.) or Methylprednisolone (10 mg/kg, p.o.) treatment for 28 days. Sham control rats received saline instead of BLM. The lung function test, biochemical, histopathological and molecular changes were analyzed in lung and bronchoalveolar lavage fluid (BALF).

**Results**

Treatment with Morin significantly restored the BLM-induced alteration in body weight, lung index, lung function test and hematology. The altered total and differential cell count in BALF and blood were significantly restored by Morin treatment. The decreased peripheral blood oxygen content after BLM instillation was significantly increased by Morin treatment. Morin significantly enhanced the BALF and lung antioxidant status, through modulating the SOD, GSH, MDA, NO level and Nrf2, HO-1 mRNA expression. Morin treatment significantly restored the altered mRNA expression of lung inflammatory markers (TNF-α, IL-1β, IL-6, and IL-8), fibrotic markers (TGF-β, collagen-1, Muc5ac, NF-kB and Smad-3) and apoptotic markers (Bax, Bcl-2, and Caspase-3). BLM-induced histological inflammatory, fibrotic insult and ultrastructural changes in the lung was reduced by Morin treatment.

**Conclusion**

Morin has potential anti-fibrotic efficacy through induction of Nrf2, which in turn modulated inflammatory, fibrotic and apoptotic molecules to reduce pathogenesis of BLM-induced pulmonary fibrosis.

References:

1. Kandhare AD, Ghosh P, Bodhankar SL. Morin attenuates airway hyperresponsiveness of allergic asthma via down regulation of immune-inflammatory biomarkers. Paper presented at: In Proceedings of the 15th International Congress of Immunology (ICI). 22 Aug - 27 Aug, 2013., 2013; Milan, Italy.

2. Kandhare AD, Bodhankar SL, Mohan V, Thakurdesai PA. Effect of glycosides based standardized fenugreek seed extract in bleomycin-induced pulmonary fibrosis in rats: Decisive role of Bax, Nrf2, NF-kappaB, Muc5ac, TNF-alpha and IL-1beta. Chem Biol Interact. 2015;237(0):151-165.

## A7 Susceptibility to exacerbations in asthma

### Nicole Grossman ^1^, Gheorghe Doros ^2^, Francine Laden ^3^, Anne Fuhlbrigge^4^, Michael Wechsler ^5^, Wilson Pace ^6^, Barbara Yawn ^7^, Elliot Israel ^4^

#### ^1^Brigham and Women Pulmonary Critical Care, Boston, MA, United States ; ^2^Harvard University, Harvard Clinical Research Institute, Boston, MA, United States ; ^3^Harvard TH Chan School of Public Health, Environmental Health, Boston, MA, United States ; ^4^Brigham and Women’s Hospital, Pulmonary Critical Care, Boston, MA, United States ; ^5^National Jewish Health, Pulmonary Critical Care, Denver, CO, United States ; ^6^American Academy of Family Physicians, Family Medicine, Denver, CO, United States ; ^7^University of Minnesota, Family and Community Health, Minneapolis, Minnesota, MN, United States

##### **Correspondence:** Nicole Grossman

**Background**

Asthma exacerbations requiring hospital admission account for more than 50% of the estimated 56 billion dollars spent each year on asthma care in the United States. Fifty percent of asthmatics report a history of an exacerbation in the prior year even though the majority of such patients have “mild” disease. The characteristics and determinants of exacerbations are incomplete.

**Objective**

In a large intervention study, we tested the hypothesis that an exacerbation prone phenotype exists in adult asthma independent of asthma control.

**Methods**

We analyzed exacerbations (requirement for corticosteroids) and their associations in a cohort of 1,070 self-identified adult Black Americans with asthma eligible for, or on, Step 3 NAEPP asthma therapy who participated in a randomized 6-18 month open-label trial of tiotropium vs. long acting beta agonist as add-on therapy to inhaled corticosteroids. Asthma control at entry was assessed by ACQ5. Severity of prior exacerbations was graded as requiring oral corticosteroids or ED visit or hospitalization. Phenotypic characteristics included age, gender, BMI, smoking history and environment, environmental allergies, years from first asthma diagnosis, baseline/study medications, short acting beta agonist (SABA) usage, spirometric indices, and additional patient-reported asthma measures (AQLQ, ASUI).

**Results**

The likelihood of a future exacerbation did increase with worsening asthma control. Exacerbations occurred in 21% of subjects with baseline ACQ≤0.75, 29% of subjects with 0.75<ACQ<1.5, and 39% of those with ACQ≥1.5 (OR per 1 SD change (1.18 units): 1.811; *P*<0.001). The strongest predictor of exacerbations was a history of exacerbations in the preceding year (OR 3.802; *P*<0.001). The severity of past exacerbations did not correlate with the likelihood of a future exacerbation. Lower baseline FEV1/FVC was also associated with increased exacerbations.

Conclusions

While exacerbations were more likely with worsening ACQ scores, there appears to be an exacerbation susceptibility phenotype independent of asthma control. This phenotype may need precision therapeutic targeting.

## A8 The macrophage response to respiratory viral infection in normal and asthmatic conditions: mathematical model

### Junehyuk Lee^1^, Frederick Adler ^2^, Peter Kim^3^

#### ^1^Soonchynhyang University Respiratory-Allergy, Bucheon, South Korea ; ^2^Utah University, Biology, SLC, UT, United States ; ^3^Sydney University, Mathematics, Sydney, Australia

##### **Correspondence:** Junehyuk Lee

**Background**

Respiratory viral infection is an important cause of aggravation and exacerbation of asthma. Despite many studies, it is not well understood how viral infections cause an exacerbation of asthma and provoke more severe symptoms in asthmatics than normal people. We constructed a mathematical model and simulate the changes of response after viral stimulation.

**Methods**

The mathematical model included two types of macrophages, classically and alternatively activated macrophages, and their related cytokines and enzymes. The normal response model was adjusted the parameters from published data and three asthma-like conditions were built with control the production rates of key-cytokines. The changes over time after viral stimulation were observed.

**Results**

A higher viral load or longer duration of infection provokes a stronger immune response from the macrophage system in the model. The model predicts that asthma-like conditions will respond differently to respiratory viral infection than normal conditions with an increased duration and magnitude of inflammatory responses.

**Conclusions**

This model explain the differences in response to respiratory viral infection in normal and asthmatic subjects, and show how this skews the system toward a response that generates more severe symptoms in asthmatic patients.

## A9 Nosocomial infections in pediatric cardiovascular surgery: how should we add anti-biofilm antibiotic?

### Yung Feng Huang^1^, Ying Yao Chen^1^, Chiun Yen Pan^2^, Herng Sheng Lee^3^

#### ^1^Kaohsiung Veterans General Hospital, Pediatrics, Kaohsiung, Taiwan ; ^2^Kaohsiung Veterans General Hospital, Cardiac Surgery, Kaohsiung, Taiwan ; ^3^Kaohsiung Veterans General Hospital, Pathology, Kaohsiung, Taiwan

##### **Correspondence:** Yung Feng Huang

**Background**

Ventilator associated pneumonia (VAP) is a frequent cause of nosocomial infection (NI) after cardiac surgery in pediatric patients, and results in a significantly longer stay in the intensive care unit. Azithromycin has been shown to retard Pseudomonas aeruginosa biofilm formation. Staphylococcus epidermidis and Staphylococcus aureus are the most frequent causes of nosocomial infections and infections on indwelling medical devices, which characteristically involve biofilms

**Objective**

This study was to determine whether an anti-biofilm antibiotic (azithromycin) reduced NI and VAP for patients undergoing pediatric cardiac surgery.

**Methods**

We enrolled 207 patients (<20 years) who underwent cardiovascular surgery for congenital heart disease. Data on postoperative courses were compared between children with and without intravenous azithromycin treatment. We administered perioperative conventional antimicrobial prophylaxis (cefazolin and gentamicin) for 3 days, with (AZI group) and without (previous group) intravenous azithromycin for 3 days. Furthermore, 78 patients from the medical record retrieval system of KVGH (Kaohsiung Veterans General Hospital) from 2012 to 2015 were recorded (NOW group).

**Results**

The previous group had higher rates of VAP infection, longer periods of ventilator dependence and length of post-operative stay in hospital than AZI Group. There was a significantly higher rate of NI in the previous group compared to the AZI group (*P* < 0.05). We also found the same trend with higher rate of NI in NOW group compared with AZI group (*P* < 0.05).

**Conclusions**

We suggest that patients only receiving conventional antimicrobial prophylaxis are more likely to have NI than those receiving additional anti-biofilm azithromycin treatment.

## A10 Emergency response community effectiveness: an analysis of anaphylaxis-related EMS events in the USA

### Michael Khalemsky^1^, David G. Schwartz^1^

#### ^1^Bar-Ilan University, Graduate School of Business Administration, Ramat Gan, Israel

##### **Correspondence:** Michael Khalemsky

**Background**

Adherence by chronic patients to medical regimens is dismally low, with under 30% of patients carrying their Adrenaline Auto Injectors (AAI) at all times (Song et. al, 2014). Rapid administration of Adrenaline is the widely accepted initial treatment for anaphylaxis. The Emergency Response Communities (ERC) approach uses a smartphone-based regulated social network of patients who are required to carry life-saving medications and can help each other in case of absence of the medication when a sudden attack occurs (Schwartz et. al, 2014). While traditional EMS response is always preferred, ERCs have the potential to augment emergency response by providing the AAI before EMS arrival.

**Objective**

To assess the applicability and potential of the ERC approach for anaphylaxis events in the USA.

**Methods**

We developed a Monte Carlo simulation-based software tool, the Emergency Response Community Effectiveness Modeler, which accepts parameters such as population density, medical condition prevalence, adherence levels, smartphones penetration, community adoption etc., to create a detailed comparison of potential smartphone-initiated Samaritan/member response to traditional EMS response. We used EMS data from the U.S. National EMS Information System (NEMSIS) and analyzed geographies based on urbanicity classifications using RUCA (Rural-Urban Commuting Area) and ERS urban influence codes. Our experiments, based on a full year (2013) of NEMSIS data explored 14,366 calls to 911 involving anaphylaxis.

**Results**

The average probability across all geographies that the ERC will be faster than EMS varies from about 3% for the worst-case scenario, to about 13% percent for the most likely scenario and up to 34% for the most optimistic scenario. There is a strong positive correlation between the population density and the expected probability that ERC will be faster than the EMS. Simulations show the ERC to be most effective in heavily populated metropolitan areas. For example, in areas with population density above 6,460 people per km2 (e.g. New York City, San Francisco etc.), the average probability that ERC will be faster is nearly 59%. The expected time savings vary from 3.72 minutes in metropolitan areas and up to 5.55 minutes in small towns and rural areas.

**Conclusions**

Our findings support that ERC is able to provide an effective addition to traditional EMS response in the USA, especially in metropolitan areas in which most anaphylaxis-related EMS calls occur (77%). For anaphylaxis patients these few minutes of faster response can be lifesaving and can improve prognosis.

## A11 About 3% of chronic spontaneous urticaria patients have vitiligo and thyroiditis (autoimmune polyglandular syndrome IIIC)

### Pavel Kolkhir^1^, Dmitry Pogorelov^1^, Nikolay Kochergin^1^

#### ^1^Sechenov First Moscow State Medical University, Dermatology and Venereology, Moscow, Russia

##### **Correspondence:** Pavel Kolkhir

**Background and objective**

Autoimmune polyglandular syndromes (APS) are a group of rare disorders characterized by autoimmune activity against endocrine and non-endocrine organs and classified into 4 types. APS type IIIC includes autoimmune thyroiditis (AIT), vitiligo and/or alopecia and/or other autoimmune disease such as chronic spontaneous urticaria (CSU). We report for the first time the prevalence of APSIIIC in CSU and make an attempt to characterize this subgroup of patients.

**Methods**

We retrospectively evaluated medical records from 234 adult CSU patients over a 3-year period. Urticaria activity score 7 (UAS7) and Chronic Urticaria Quality of Life Questionnaire (CU-Q2oL) results were obtained and autologous serum skin test (ASST) was performed. Vitiligo was diagnosed on clinical grounds, AIT – on anti-thyroid antibody tests. The thyroid status was determined by measuring TSH, T3, T4 blood levels. The patients were treated in accordance with EAACI/GA(2)LEN/EDF/WAO guidelines. Comparison between different groups of patients was carried out using the chi-square test.

**Results**

Twenty three percent (55/234) of patients had the combination of CSU and AIT, 1.3% (3/234) – had CSU and vitiligo and 2.6% (6/234) were diagnosed with APSIIIC. APSIIIC patients comprised 66.7% (6/9) of the total number of patients who had CSU and vitiligo. In contrast, APSIIIC was seen in only 9.8% (6/61) of patients with CSU and AIT (χ2=17.833, *p*<0.001, V=0.505). Five APSIIIC patients were female and 1 was male; the age varied from 34 to 67 years. The duration of CSU was from 6 months to 13 years (mean: 5 years 3 months). All patients had generalized vitiligo for 5-35 years (mean: 19 years). The duration of AIT was from 6 months to 21 years (mean: 12 years). ASST was strongly positive in all cases. APSIIIC patients were ASST positive significantly more often than CSU patients without AIT and/or vitiligo (100% vs 58.3%, χ2=4.062, *p*=0.044, V=0.228). The patients were followed for a period from 1 to 38 months. We observed the efficacy of cyclosporine A (CsA) in 3 patients with poor response to updosed antihistamines. In our male patient, adding CsA to high doses of antihistamines led to improvement in CSU symptoms, induction of remission and marked reduction of vitiligo lesions.

**Conclusions**

We found that APSIIIC occurs in 2.6% of CSU patients. Vitiligo preceded CSU development in all cases and may be a prognostic factor for APSIIIC in CSU patients. Genetic and autoimmune mechanisms may explain the presence of different components of APS.

## A12 Mast cell degranulation in physical urticaria-dissociation of histamine and tryptase

### Hirsh Komarow^1^, Michael Young^2^, Robin Eisch^1^, Linda Scott^1^, Dean Metcalfe^1^

#### ^1^National Institute of Allergy and Infectious Diseases, National Institutes of Health, Mast Cell Biology Section, Laboratory of Allergic Diseases, Bethesda, MD, United States ; ^2^ Leidos Biomedical Research, Inc., Frederick National Laboratory for Cancer Research Clinical Research Directorate/Clinical Monitoring Research Program, Frederick, MD, United States

##### **Correspondence:** Hirsh Komarow

**Background**

The urticarial lesions of physical urticaria are thought to be the result of mast cell activation and degranulation, which is supported by the finding of increased serum levels of mast cell mediators during some urticarial flares.

**Objective**

To determine serum levels of histamine and tryptase, mast cell markers of degranulation, following challenge testing in patients with physical urticaria.

**Methods**

An IV catheter was placed in 10 patients with documented physical urticaria and 8 control subjects. Serial blood draws were obtained at baseline and at specific time points during and after urticarial challenge testing. Challenge testing for cold urticaria consisted of cold hand water submersion (10° C) to 2 inches above the wrist for 5 minutes; for vibratory urticaria, vortex vibratory stimulation for 4 minutes at 2400 rpm; and for cholinergic urticaria, 15-25 minute treadmill exercise until there was profuse sweating with continued exercise for >10 minutes. Samples were assayed in patients and control subjects for histamine and tryptase to determine the onset, peak and drop in serum mediator levels.

**Results**

Evidence for mast cell degranulation was established by examination of skin biopsies and documentation of a significant increase in serum histamine level with challenge testing. In subjects with cold urticaria (*n*=7) the mean serum peak for histamine was 45.5 nM and occurred at 5 min post challenge, for patients with vibratory urticaria (*n*=2) the peak was 110 nM at 2 min post challenge and for one patient with cholinergic urticaria after 20 min of exercise, the peak reached 300 nM. There were no significant increases in histamine levels in control patients. Evidence of mediator release resolved in all patients by 30 minutes except for the one patient with cholinergic urticara where serum histamine was still elevated 120 minutes after challenge. Corresponding tryptase levels did not change significantly (range 2-10 ng/mL) when assayed through 60-120 minutes in all subjects despite histochemical evidence of local release of mast cell tryptase.

**Conclusions**

Challenge testing in patients with cold, vibratory and cholinergic urticaria induces an early onset and significant peak in serum histamine thus implicating mast cell degranulation in the pathogenesis, yet increased tryptase was not detectable. The discordant ability of histamine and tryptase to reach the systemic circulation may reflect upon the unique characteristics of these mediators and their release in physical urticaria.

## A13 Retrospective review of beta lactam allergy prevalence in a referral population

### Alexander Singer^1^, Andrew Wakeman, Thomas Gerstner^3^, Elissa Abrams^3^

#### ^1^University of Manitoba, Family Medicine, Winnipeg, Canada ; ^2^UCD Medical School, Dublin, Ireland ; ^3^University of Manitoba, Paediatric Allergy, Winnipeg, Canada

##### **Correspondence:** Alexander Singer

**Background**

Penicillin allergies are over diagnosed. About 90% of those avoiding penicillin class antibiotics are tolerant when allergy tested. In addition, most patients with true penicillin allergy will lose their sensitivity over time. The label ‘penicillin allergic’ is linked with poorer health outcomes and higher healthcare costs.

**Methods**

A retrospective chart review was performed for all patients evaluated for beta lactam allergy from January 2010 to June 2015 in a community allergy clinic. Evaluation was by intradermal testing and/or oral drug provocation test when deemed appropriate. Final outcomes were decided by a consensus of consultant investigators based on reaction history and results of skin and/or oral drug provocation testing.

**Results**

Our sample includes 306 referred patients with a recent history of a reaction to a beta lactam antibiotic. Mean age was 11.6 (SD 17.3); 49.5% of patients were male. Most reactions were to amoxicillin, and most reactions were reported as mild non-urticarial skin reactions. There were only 1/106 (0.009%) positive intradermal tests. Only 2/191 (0.01%) patients reacted with an oral drug provocation test with symptoms consistent with IgE-mediated hypersensitivity. Six patients were deemed unfit candidates for testing based on elements in reaction history suggestive of a serious delayed reaction. Four patients had delayed non-urticarial exanthems after oral drug provocation testing. Overall, 294 patients (96.1%) of those evaluated were determined not to require future avoidance of any beta lactam antibiotic. Increased risk of anaphylaxis was ruled out in 99.3% of cases.

**Conclusions**

Patients with documented beta lactam allergy were rarely allergic upon evaluation. This supports arguments for strategic widespread testing and delabelling of ‘penicillin allergic’ outpatient populations, particularly for pediatric patients.

## A14 Diagnostic accuracy of fractional exhaled nitric oxide measurement for cough variant asthma and eosinophilic bronchitis in adult patients with chronic cough: a meta-analysis

### Woo-Jung Song^1^, Ji-Su Shim^1^, Ha-Kyeong Won^1^, Sung-Yoon Kang^1^, Kyoung-Hee Sohn^1^, Byung-Keun Kim^1^, Eun-Jung Jo^2^, Min-Hye Kim^3^, Sang-Heon Kim^4^, Heung-Woo Park^1^, Sun-Sin Kim^1^, Yoon-Seok Chang^1^, Alyn H Morice^5^, Byung-Jae Lee^6^, Sang-Heon Cho^1^, Kyung-Up Min^1^

#### ^1^Seoul National University, College of Medicine, Department of Internal Medicine, Seoul, South Korea ; ^2^Pusan National University, College of Medicine, Department of Internal Medicine, Busan, South Korea ; ^3^Ewha Woman’s University, School of Medicine, Department of Internal Medicine, Seoul, South Korea ; ^4^ Hanyang University, College of Medicine, Department of Internal Medicine, Seoul, South Korea ; ^5^Hull York Medical School, Castle Hill Hospital, University of Hull, Cottingham Centre for Cardiovascular and Metabolic Research, Cottingham, United Kingdom ; ^6^Sungkyunkwan University, School of Medicine, Department of Medicine, Seoul, South Korea

##### **Correspondence:** Kyung-Up Min

**Background**

Cough variant asthma (CVA) and eosinophilic bronchitis (EB) are two major conditions presenting as chronic cough; however, conventional methacholine challenge and induced sputum tests are technically demanding and have been restricted to specialized centers. Fractional exhaled nitric oxide (FeNO) measurement is a simple, rapid and non-invasive test, and could be a point-of-care alternative to the conventional diagnostics in chronic cough.

**Objective**

To obtain summary estimates of diagnostic test accuracy of FeNO in predicting CVA and/or EB in adult patients with chronic cough

Methods

Electronic databases were searched for studies published until January 2016, without language restriction. Cross-sectional studies which reported the diagnostic accuracy of FeNO for detecting CVA or EB were included. Risk of bias was assessed with QUADAS-2. Random effects meta-analyses were performed to obtain summary estimates of the diagnostic accuracy of FeNO.

**Results**

A total of 15 studies involving 2,187 adult patients with chronic cough were identified. FeNO had a moderate diagnostic accuracy in predicting CVA in patients with chronic cough, showing the summary area under curve (AUC) to be 0.87 (95% CI 0.83-0.89). Specificity was higher and more consistent than sensitivity. However, in non-asthmatic chronic cough population, the diagnostic accuracy to predict EB was found to be relatively lower (the summary AUC 0.81 [95% CI 0.77-0.84]) and specificity was inconsistent.

**Conclusions**

The present meta-analyses indicated the diagnostic potential of FeNO as a ‘rule in’ test for detecting CVA in adult patients with chronic cough. However, FeNO may not be useful to predict EB in non-asthmatic chronic cough. These findings warrant further studies to validate the roles of FeNO in clinical practice of chronic cough patients.

## A15 Evaluation of oral desensitization with nickel (Tionickel) in patients suffering from SNAS

### Maria Assunta Boscolo ^1^, Giulio Brivio ^1^, Sergio Bosisio ^1^, Nicoletta Manzocchi ^1^, Edoardo Pulixi ^1^, Giulia Grignani ^1^, Eloisia D’Andrea ^1^, Massimo Ricci ^1^, Elena Passini ^1^, Maurizio Italia ^1^

#### ^1^Merate Hospita,l Allergology, Osnago, Italy

##### **Correspondence:** Maria Assunta Boscolo

**Background**

The SNAS (Systemic Nickel Allergy Syndrome) appears with symptoms in various organs (oculorhinitis, asthma, eczema, urticaria, abdominal pain, diarrhoea, epigastric pain) in a fair percentage of patients suffering from proven ACD (Allergic Contact Dermatitis), caused by hypersensivity to nickel.

**Objective**

The object of the research is the evaluation of the effectiveness of the desensitization with Tionickel (Lofarma) associated with a low-nickel diet, in patients suffering from SNAS, in comparison with the mere low-nickel diet.

**Methods**

The research was carried out on 66 patients over a period of 3 years.

62 males and 4 females aged between 18 and 68 (average age 36).

26 patients opted to follow the mere low-nickel diet.

40 patients chose to follow a low-nickel diet + desensitization with Tionickel.

**Results**

The 26 patients treated with the mere diet did not suffer from any symptoms during the period in which they were following the diet. The symptoms appeared again, much more slightly, with the interruption of the diet. During check challenges, the symptoms showed up with higher doses of nickel.

Out of 40 patients treated with Tionickel + diet:

7 interrupted the therapy due to considerable side effects.

2 interrupted the treatment due to pregnancy.

10 follow a free diet with no symptoms after the end of therapy.

21 suffer from occasional and slight symptoms when they eat food containing a quantity of nickel, which is higher than 500 mg/kg

**Conclusions**

Desensitization with Tionickel associated with a low-nickel diet has shown a clear effectiveness in terms of reduction of the symptoms, in comparison with patients following the mere diet.

The course of desensitization with Tionickel could not be completed on a considerable number of patients (7 over 40), due to serious side effects.

## A16 Is smoking an enabler to experiment with other drugs for teenagers living in a small city in the South of Brazil?

### Marilyn Urrutia-Pereira^1^, Stefani Fagundes^2^, Vinicius Jardim Oliano^3^, Dirceu Solé ^4^

#### ^1^Federal University of Pampas (Unipampa), Children Pediatric, Uruguaiana, Brazil ; ^2^Federal University of Pampas (Unipampa), Pediatrics, Uruguaiana, Brazil ; ^3^University of Campanha Region (URCAMP), Pediatrics, Uruguaiana, Brazil ; ^4^Federal University of São Paulo Allergy, Clinical Immunology and Rheumatology, São Paulo, Brazil

##### **Correspondence:** Marilyn Urrutia-Pereira

**Background**

Despite anti-smoking prevention programs, many teens start smoking at school age.

**Objective**

Our objectives were to determine the prevalence and risk factors associated with smoking in adolescents living in Uruguaiana, RS, Brazil.

**Methods**

A thousand public school teenagers living in the city of Uruguaiana RS, Brazil, participated in this study, answering the modified California Smoking Survey, where questions on use of alcohol and cannabis were added.

**Results**

According to the fact of experimenting smoking at least once they were considered experimenters (Ex; 35,7%) the ones who did not, non-experimenters (NEx; 64,3%). Both groups were similar as to vender. Among Ex adolescents 22.7% have chronic respiratory problems, 79.8% reported having ease in getting cigarettes, 39.7% having been affected by a smoker friend. 13,7% of them referred to use electronic cigarettes, 39.2% try hookah and 56% live with mother’s smokers. The frequency of having used an alcoholic beverage and/or smoked cannabis, were significantly high among Ex in comparison to NEx. The beginning of smoking coincided with the beginning of ingestion of alcohol and they were significantly relevant before 12 years of age. The knowledge and the use of other forms of cigarette (electronic and hookah) were significantly higher among Ex. The teenagers do not associate them to health hazards.

**Conclusions**

The prevalence of tobacco experimentation is high, starting early on life and is associated with the increased use of other drugs: alcohol and cannabis.

## A17 Comprehensive evaluation before thermoplasty treatment: The University of Cincinnati experience

### Sadia Benzaquen^1^, Alejandro Aragaki^1^, Ricardo Balestra, Dawn Harden^1^, Danielle Caudell-Stamper^1^

#### ^1^University of Cincinnati, Pulmonary, Cincinnati, OH, United States

##### **Correspondence:** Sadia Benzaquen

Bronchial thermoplasty (BT) is a new endoscopic treatment for severe persistent asthma which applies heat temperature (65 degrees Celsius) to the small airway for 10 seconds causing atrophy of the small muscle. The treatment is performed as an outpatient procedure on 3 different occasions, each 3 weeks apart. Unfortunately, the cost and insurance approval remain major issues in the US. In order to obtain insurance approval, we have created a comprehensive work-up for people with severe persistent asthma who are referred to the IP (interventional pulmonology) clinic at the University of Cincinnati.

All of the patients attend an office visit with complete PFTs with and without BD response. If the PFTs do not show obstruction or BD response, the patients get a methacholine challenge test to rule out the diagnosis of asthma.

Before consideration for BT, all of the patients get a dynamic bronchoscopy to rule out VCD, laryngopharyngeal reflux disease or EDAC (excessive dynamic airway collapse). All of the patients get allergy testing, total IGE and IGG/IGE anti aspergillus. Finally, we order a HRCT to rule out bronchiectasis. Depending on the findings of the initial work up, they may need other procedures, such as an extensive work up for GERD.

Between January 2011 and January 2015, we evaluated a total of 50 patients who were referred for thermoplasty. The mean age was 49 years. The sample was 68% female and 32% male. Seventy-four percent of the patients had obesity or morbid obesity. Medication use was as follows: 80% on oral corticosteroids, 90% on ICS, 95% on a LABA, 96% on SABA, 2% on short anticholinergic, 18% on long acting anticholinergic, 56% on antileukotriene receptor antaganoist, 20% on Xolair, and 4% on amynophiline. The mean FeVi1 1.99 liters. Interestingly, we found that 56% of the patients had another diagnosis in addition to asthma. Specifically, 30% had excessive dynamic airway collapse, 8% had VCD, and 18% had LPR. Of all the screened patients, only 44% completed the thermoplasty treatment. Forty-two percent were good candidates for thermoplasty, but never got the approval by their insurance, and 14 percent were not appropriate candidates for thermoplasty.

In conclusion, we believe that thermoplasty is another tool that may benefit our patients with severe persistent asthma. It is important to select the appropriate patients in order to get insurance approval and have a high treatment success rate.

## A18 An oral immunotherapy for treatment of asthma and other allergic airway diseases by using microRNAs

### Gilbert Glady

#### EBMA clinical research, Colmar, France

**Background**

Rhinitis and asthma are highly prevalent chronic diseases prevalent in both developed and developing countries, where a lot of people of all ages and ethnic backgrounds are affected. Evidences indicate that etiology of asthma and allergic diseases is complex and has strong genetic and environmental components.

**Objective**

All these data explain the large number of treatments available today, but not always satisfying related to long term efficacy and safety. It may therefore be of interest to have a biomimetic treatment globally recovering the different molecular and cellular mechanisms involved in these allergic diseases.

**Methods**

The inflammatory process in the airways displays several common characteristics in asthma and allergic rhinitis, e.g. IgE-dependent activation of mast cells, infiltration of eosinophils, and an increase in the number of T4-lymphocytes and Th-2 type cytokine concentrations. In addition, cytokines associated with regulatory T-cells and Th1 and Th17 cells have been found to be essential.

microRNAs (miRNAs) are short, single-stranded RNA molecules, that cause degradation of target mRNAs or inhibit their translation. Each miRNA can have one or more target transcripts, while each transcript may be regulated by one or more microRNAs; therefore, miRNAs cumulatively influence the expression of a large proportion of genes.

Current knowledge shows different specific mechanisms by which miRNAs impact allergic inflammation in tissues: - polarization of Th2 cells, - development and functions of T8-cells and innate immune cells found in or recruited into the inflamed tissue, - chronic inflammation through effects in epithelial cells.

As miRNAs can be either inhibited or overexpressed, there is an obvious potential for miRNAs as novel target molecules for the development of biological therapeutics.

**Results**

To avoid any side effects, our team prepare microRNAs on a nanobiological level by using high dilutions of them; it follows a sublingual administration to reach quickly the main immune cells and molecules.

A clinical study with 61 patients showed that this kind of sub-lingual immunotherapy could allow to reduce a corticotherapy significantly and improve several clinical respiratory symptoms by a majority of these asthma patients.

**Conclusions**

So it becomes possible to regulate chronic allergic diseases like asthma and rhinitis by using the own regulatory means of the immune system without penalizing it otherwhere.

## A19 New onset soy-related exercise induced anaplylaxis (EIA) in adolesences with peanut allergy

### Mark Holbreich

#### Allergy Consultants, Indianapolis, IN, United States

**Background**

The incidence of peanut allergy in the United States is rising dramatically. We have recognized a number of peanut allergic patients who have previously tolerated soy who developed soy-related exercise induced anaphylaxis (EIA) in adolescence.

**Objective**

We hope to make allergists aware of the risk of soy-related EIA in their peanut allergic patients as they reach adolescence.

**Methods**

The charts of 3 patients who presented with soy-related EIA were reviewed for clinical data related to peanut allergy and soy-related EIA.

**Results**

Case #1 is a 17 year old male diagnosed with peanut allergy at age 1 year. At age 13 he ingested a soy butter sandwich at school. Then during physical activity that followed immediately after eating the meal he developed EIA. He was treated with antihistamines and albuterol and symptoms resolved. His prick skin test (PST) was positive to soy butter. He had previously been tolerating soy. Case #2 is a 15 year old male diagnosed with peanut allergy at age 1 year. At age 12 years he developed EIA after eating a lunch patty at school. The patty contained soy protein concentrate. His PST was positive to soy as well as to the lunch patty. He has previously tolerated soy. Case #3 is a 14 year old female diagnosed with peanut allergy at age 1 year. Following ingestion of a portion of soybean (edamame) she developed EIA during a swim practiced that followed the meal. She required albuterol and injected epinephrine as well as an emergency room visit. Her PST was positive to edamame.

**Conclusion**

We suspect that allergists will be seeing more soy-related EIA as their young patients with severe peanut allergy approach adolescence. The mechanism of this is not well understood. Soy is a frequent additive to foods and the diagnosis of soy-related EIA places new burdens on these families and children.

**Consent**

The author received informed consent from the patients’ parents to publish.

## A20 The role of gene polymorphism of Toll-like receptors 2,4 and Clara cell protein in the development of asthma in adults

### Nataliya Lyakhovska, Igor Kaidashev

#### Supreme State educational institution of Ukraine “Ukrainian Medical Stomatological Academy”, Ministry of Health, Poltava, Ukraine

##### **Correspondence:** Nataliya Lyakhovska

**Background**

Genetic aspects of asthma and atopy have been widely studied. Candidate genes and loci of chromosomes probably responsible for the occurrence of bronchial asthma (BA) are defined by a large amount.

**Objective**

The aim of our work was to study polymorphisms 2258G / A gene TLR2 (rs5743708) and 896A / G genaTLR4 (rs4986791), Clara cell protein gene (A38G), with a specific weight of 16 kDa (CC16) in the adult population. We examined 45 patients with asthma in the period without exacerbation.

**Methods**

Diagnosis of asthma and severity was approved accordance with the criteria GINA. To determine the sensitivity to allergens used skin prick test. Determination of polymorphisms TLR2 gene and gene TLR4, gene CC16 carried by polymerase chain reaction with special primers.

**Results**

Patients with BA significantly more common had genotype GA (11,1%) gene TLR2 (*p* = 0.04) compared with the control group. In patients who are carriers of a mutant allele of a gene TLR2 A history pneumonia frequently observed (*p* = 0.046) and there were signs of candidiasis (*p* = 0.034) compared with patients with no polymorphism. In the study of polymorphism of TLR 4 found that genotype AG statistically more likely (*p* = 0.04) is found in the BA group (15.6%) than in the control group. Patients with polymorphism 896A/G TLR4 gene disease begins in childhood (*p* = 0.03) in the spectrum of sensitization were dietary factors (*p* = 0.02) and there were other manifestations of allergic diseases (*p* = 0.045). Polymorphic variant gene 38G CC16 significantly more common in patients with BA than in the control population (*p* = 0.019). Clinical manifestations in patients with BA who are carriers of the gene allele 38G CC16 are fungal sensitization, atopic dermatitis and history of tuberculosis, the need for frequent doses of glucocorticoids.

**Conclusions**

The study of polymorphisms 896A / G gene TLR4 and 2258G/A TLR2 gene, A38G gene CC16 gene demonstrated their direct impact on the pathogenetic features of bronchial asthma. And is the basis for our next research of biomarkers of allergic inflammation (IgE, Il-4, Il-10), T-reg cell in patients who are carriers of polymorphisms of genes studied.

## A21 The effect of anti-IgE monoclonal antibody on other allergic comorbidities of severe asthma patients

### Jaromir Bystron, Beata Hutyrova

#### University Hospital Allergology and Clin. Immunology, Olomouc, Czech Republic

##### **Correspondence:** Jaromir Bystron

**Background**

Omalizumab is approved for the treatment of severe persistent allergic asthma and chronic spontaneous urticaria, although a number of studies confirmed the effectiveness of this therapy also on other IgE-mediated diseased.

**Objective**

The aim of this study was to evaluate the effect of anti-IgE treatment on other allergic diseases in patients treated with omalizumab for severe allergic asthma enrolled in the CAR (Czech Anti-IgE Registry).

**Results**

In 209 patients included in the CAR the effect of treatment with omalizumab on clinical manifestations of allergic rhinitis (*n* = 185), atopic dermatitis (*n* = 63), food allergy (*n* = 55), and chronic urticaria (*n* = 12) after 12 months of therapy was also evaluated. The positive effect of treatment with omalizumab was observed in 83.3% of patients with allergic rhinitis (improvement in 64.9% of patients, complete remission of symptoms in 18.4% of patients). Improvement of chronic urticaria was reported in 41.7% of patients and complete remission also observed in 41.7% of subjects. In 46.0% of patients the improvement of atopic dermatitis was observed and 34.9% of patients had no symptoms of skin disease. Food allergy was improved in 25.5% of patients and in 38.2% of patients the symptoms have subsided. In food allergies, compared to other monitored diseases, there was the highest percentage of patients (32.7%) with no change in symptoms during treatment with omalizumab.

**Conclusions**

In the CAR registry of patients with severe allergic asthma treated with omalizumab the positive effect of anti-IgE therapy on other allergic comorbidities was found. Majority of patients with allergic rhinitis, atopic dermatitis, chronic urticaria and food allergies experienced the improvement or complete remission of the symptoms of particular diseases.

## A22 Anaphylaxis caused by chicory might be associated with sensitization to cannabis

### Galina Balakirski^1^, Luk Vanstreels^1^, Gerda Wurpts^1^, Hans F Merk^1^, Jens Malte Baron^1^, Johanna Plange^1^, Hans-Peter Rihs^2^, Monika Raulf^2^, Stefani Roeseler^1^

#### ^1^University Hospital of RWTH Aachen, Department of Dermatology and Allergology, Aachen, Germany ; ^2^Ruhr-University Bochum, Institute for Prevention and Occupational Medicine of the German Social Accident Insurance, Bochum, Germany

##### **Correspondence:** Galina Balakirski

**Background**

A 31 years old female patient developed severe facial angioedema shortly after a dinner with turkey, potatoes, chicory and a ready-made sauce. She also had some Schweppes® together with a pill out of acetylsalicylic acid, paracetamol and caffeine during the meal due to headache. In our department she recovered rapidly after intravenous administration of prednisolone, dimetindene and ranitidine.

**Objective**

In order to explore the cause of the anaphylactic reaction the patient consumed turkey, potatoes, a ready-made sauce and Schweppes® once more, however tolerated the meal without any problems. She also took a pill containing paracetamol and caffeine and experienced no reaction after this self-experiment.

Anamnestically the patient reported to suffer from signs and symptoms of allergic rhinoconjunctivitis due to the known sensitization to tree and grass pollen, and to house dust mites. Furthermore she experienced signs and symptoms of an oral allergy syndrome by nuts. She also reported to smoke marijuana frequently. During preparation of the joints the patient described symptoms of allergic rhinoconjunctivitis with itchy eyes, sneezing, and runny nose, but not during marijuana smoking.

**Methods**

We performed a skin prick test with analgesics including acetylsalicylic acid with a negative result. The prick to prick test with chicory (>10 mm) and cannabis (7 mm) were positive. Specific (s) IgE was detected with the ImmunoCAP system: sIgE to rPru p 3 (lipid transfer protein (LTP) of peach) was CAP class 3, sIgE to rCan s 3 (LTP of Cannabis sativa) was CAP class 2.

**Results**

Only few cases of anaphylactic reactions caused by chicory are reported in the literature. The most common allergen (in over 90% of cases) is LTP of lettuce Lac s 1, which shows cross-reactivity to Pru p 3. The LTP of marijuana Can s 3 is also described with cross-reactivity to Pru p 3 (1).

**Conclusions**

In the literature case reports about patients with symptoms of allergic rhinoconjunctivitis due to marijuana exist, who developed allergic symptoms including anaphylaxis after consuming tomato or peach months later [2]. The suggested mechanism is a cross-reaction of LTP Can s 3 to peach (Pru p 3) and tomato (Lyc e 3) [1,2]. Also in our case we assumed a primary sensitization to Can s 3 with cross-reaction to other LTPs (i.e. Pru p 3, Lac s 1, Cor a 8), that could explain an anaphylactic reaction after consuming chicory.

**Consent**

The authors received informed consent from the patient to publish.

References

1. Armentia A et al.J Allergy Clin Immunol Pract 2014;2(3):351-2

2. Gamboa P et al. J Allergy Clin Immunol 2007;120(6):1459-60

## A23 A comprehensive environmental and building pathology assessment of asthmatic children in Buenos Aires, Argentina

### Alberto Tolcachier^1^, Armando Chamorro, Ruth Otero^2^

#### ^1^Durand Hospital, Allergy, Buenos Aires, Argentina ; ^2^Soluciones Ambientales CIH, Buenos Aires, Argentina

##### **Correspondence:** Alberto Tolcachier

**Background**

Indoor air quality in a child’s dwelling is a matter of concern because its deterioration often leads to development of allergies and asthma. Numerous studies justify the application of interventions to reduce respiratory symptoms associated with environmental burden of allergens, especially in children. Additinally, several meta-analyzes and guidelines warn about the importance of avoiding the potential environmental pollutants that may trigger or exacerbate respiratory symptoms, and state recommendations concerning measures of hygiene, temperature control, humidity exposures and maintenance of building structures.

**Objective**

Given the lack of accurate environmental investigations in homes of asthmatic children in Argentina, a pilot study of 10 selected homes in the City of Buenos Aires and its surroundings, inhabited by asthmatic children was conducted.

**Methods**

The home environmental evaluation included the potential asthma-triggering contaminants, allergens, airborne particulate characterization, thermal comfort, Infrared thermography and building envelope moisture levels and air pollutants to which the child might be exposed, utilizing resources from the Centers for Disease Control and Prevention (CDC), US Environmental Protection Agency (EPA), and various Healthy Homes Initiatives. Building forensic pathology elements, including home envelope analysis and cooling and heating units, that may contribute to poor indoor air quality have also been incorporated into the evaluation.

**Results**

The results of the study show an overwhelming evidence of building failure causes coupled with in some cases a significant level of measured allergens in indoor samples that can potentially trigger allergenic and asthma related symptoms to the youth.

**Conclusions**

This study focuses on finding potential environmental contaminants that may trigger or exacerbate respiratory challenges within the youths, in order to provide residents of those homes precise guidelines for building repairs, renovations, remediation and mitigation of building related failures and allergen control, based on real-life scenario. To date, no study is known to have specifically addressed this issue in Argentina.

## A24 Hypersensitivity pneumonitis in a farmer

### Joel Brooks^1^, Michael Hess^2^, Jared Benz^2^, Joseph MacDonald^2^

#### ^1^Yale University, School of Medicine, Allergy and Immunology, New Haven, CT, United States ; ^2^Heart of Lancaster Regional Medical Center, Internal Medicine, Lititz, PA, United States

##### **Correspondence:** Joel Brooks

**Background**

Hypersensitivity pneumonitis (HP) is a complex syndrome resulting from repeated exposures to a variety of organic particles. These antigens provoke an immune response in lung parenchyma and small airways. It presents as an acute, sub-acute, or chronic condition and occurs in occupational, home, and recreational settings. There are several proposed diagnostic criteria for HP including exposure to an offending antigen, respiratory signs and symptoms, radiographic findings (such as reticular, ground glass, or nodular opacities), altered spirometry and lung volumes, bronchoalveolar lavage with lymphocytosis, histopathology showing mononuclear cell infiltrate or noncaseating granulomas, or positive inhalation testing to the antigen or environment.

**Objective**

Recognize the clinical features of hypersensitivity pneumonitis and assess and treat patients presenting with this condition.

**Methods**

A 53 year old male with history of asthma presented to his primary care physician (PCP) after experiencing several days of fevers, wheezing, and shortness of breath. He was working in his grain silo two days prior with possible mold and pesticide exposures. His PCP prescribed a short course of corticosteroids. There was no improvement in symptoms and the patient presented to the emergency department. His initial oxygen saturation was in the low 80s requiring five liters of oxygen via nasal cannula. On exam, the patient had extensive crackles and rales diffusely throughout all lung fields. His labs were unremarkable except for an elevated CRP. His chest x-ray showed bilateral alveolar infiltrates. His constellation of signs and symptoms were consistent with hypersensitivity pneumonitis. The patient was placed on Solumedrol 40 MG IV twice daily for 48 hours and albuterol/ipratropium nebulized treatments with clinical improvement before transitioning to an oral prednisone taper.

**Results**

Farmer’s lung affects 0.4%-7% of the farming population and is one of the most common forms of HP. The prevalence varies by region, with approximately 9% of famers affected in the humid zones and 2% in drier zones Treatment consists of antigen avoidance and corticosteroid therapy with the recommended dose of prednisone 0.5-1mg/kg daily for 1-2 weeks, with a 2-4 week taper. Approximately 50% of farmers with this condition develop minor lung functional abnormalities. The recovery process may take years after the antigen exposure ceases.

**Conclusions**

Hypersensitivity pneumonitis must be considered in populations such as farmers who are commonly exposed to inciting antigens. The mainstay of treatment is atigen avoidance with a corticosteroids course. Maintenance steroids are not usually necessary and the majority of patients recover with minimal functional lung deficits.

**Consent**

The authors received informed consent from the patient to publish.

## A25 Respiratory distress as the initial manifestation of microscopic polyangiitis

### Joel Brooks^1^, Jared Benz^2^, Usma Chatha^2^, Dale Lent^2^

#### ^1^Yale University, School of Medicine, Allergy and Immunology, New Haven, CT, United States ; ^2^Heart of Lancaster Regional Medical Center Internal Medicine, Lititz, PA, United States

##### **Correspondence:** Joel Brooks

**Background**

Microscopic polyangiitis (MPA) is a systemic necrotizing vasculitis that affects small vessels in multiple organs, including the kidneys and lungs. Initial presentation includes fatigue, fevers, arthralgias, and weight loss. With progression, patients develop glomerulonephritis. Pulmonary involvement, commonly alveolar hemorrhage, is frequent and manifests with dyspnea, cough, or hemoptysis. The detection of anti-neutrophil cytoplasmic antibody (ANCA) and anti-myeloperoxidase (MPO) antibody are useful serologic markers for diagnosis.

**Objective**

Recognize the clinical features of MPA and assess and treat patients presenting with this condition.

**Methods**

An 87-year-old Caucasian female with history of giant cell arteritis (GCA) and stage III chronic kidney disease was hospitalized with respiratory failure, fatigue, dry cough, and lower extremity edema. A CT revealed bilateral infiltrates. Laboratory studies included an ESR >100, creatinine of 4.7 (baseline: 1.4), hemoglobin of 6.1, proteinuria, and hematuria with fine granular casts. Concern was raised for vasculitis with alveolar hemorrhage and steroids were initiated. A thorough rheumatologic workup was performed including ANCA studies. She declined a renal biopsy. Her condition deteriorated and she was transitioned to comfort measures and expired. Posthumously, her anti-MPO antibody returned elevated (605.7), supporting the diagnosis of MPA.

**Results**

MPA, granulomatosis with polyangiitis (GPA), and eosinophilic granulomatosis with polyangiitis (EGPA) are the small vessel vasculitis that are known as the ANCA-associated vasculitis. There are shared features between these conditions including renal involvement with pauci immune focal segmental necrotizing glomerulonephritis. GPA is most commonly associated with a positive anti-PR3 (c-ANCA), while EGPA and MPA are more commonly associated with a positive p-ANCA. The anti-MPO (p-ANCA) is most commonly associated with MPA, but it can appear positive in any ANCA-associated vasculitis. Therefore, diagnosis must be made by combing the clinical features with the diagnostic findings. Therapy includes high dose corticosteroids with immunosuppressive agents. Plasma exchange is beneficial in severe renal disease and alveolar hemorrhage. A unique feature of this case was the patient’s history of GCA, which is linked to several inflammatory conditions.

**Conclusions**

MPA is diagnosed by combining clinical suspicion with laboratory findings. A broad differential must be maintained when treating patients with suspected vasculitis. Vasculitic conditions are associated with poor prognoses without timely treatment with glucocorticoids and immunosuppressive agents. As there is a variable prognosis, prompt treatment must be initiated to decrease morbidity and mortality.

**Consent**

The authors received informed consent from the patient to publish.

## A26 Relationship between nasal polyposis and serum level of vitamin D

### Şükran Köse ^1^, Bengü Gireniz Tatar ^2^, Gülgün Akkoçlu ^2^, İbrahim Çukurova ^3^, İlker Ödemiş ^2^, Ayşin Kılınç Toker ^2^

#### ^1^Tepecik Training and Research Hospital, Infectious Diseases and Clinical Microbiology and Immunology, izmir, Turkey ; ^2^Tepecik Training and Research Hospital, Infectious Diseases and Clinical Microbiology, izmir, Turkey ; ^3^Tepecik Training and Research Hospital, Department of Otorhinolaryngology, izmir, Turkey

##### **Correspondence:** Şükran Köse

**Background**

Nasal polyps are one of the most common mass lesions of the nose. Nasal Polyposis (NP) is an inflammatory condition of unknown etiology. Treatment of NP is a significant challenge to the physicians. Also some genetic factors are implicated in the pathogenesis of NP, but gene expressions required for its development are still unclear. The understanding of the pathophysiology underlying cause in NP is necessary to treatment options.

**Objective**

In this study we aimed to invastigate relationship between nasal polyposis and serum level of vitamin d has been proven to have immunomodulatory effects.

**Methods**

A total of 41 NP patients and 40 healthy controls were included in the study, from January 2013 to December 2015 at the Izmir Tepecik Training and Research Hospital. The study was performed retrospectively. In study patients and control groups, serum 25-hydroxyvitamin D[25(OH)D] levels were studied by chemiluminescence immunoassay.

**Results**

The average age of NP patients is 48,4 and control group is 46,2. 50% of the control group and 44% of NP patients are female gender. Serum 25(OH)D levels were detected in NP patients and healthy controls below normal range. Serum 25(OH)D level was 13.6 ng/ml in NP patients and 12.9 ng/ml in the control group. There was no significant statistical difference between groups (p> 0.05). In both groups, serum 25(OH)D levels were lower in women than men.

**Conclusions**

The etiology of NP is unclear and it is known to have associations with allergy, asthma, cystic fibrosis and aspirin sensitivity. This situation shows that systemic rather than local involvement. Treatment of NP involves a combination of medical therapy and surgery. Different studies have shown that vitamin d have the immunomodulatory effects. In our study found low serum levels of 25(OH)D in patients both study and control groups. In comparison with serum levels of 25(OH)D between study and control group, there is no significant difference. We believe that additional studies have more number of patients, are useful.

## A27 IgE levels among non Ahmadu Bello University staff attending the sick bay of the University in Samaru, Zaria, Nigeria

### Abdullahi Hasssan^1^, Abdulrazaq Abdullahi Gobir^2^

#### ^1^Federal medical Centre Scientific Research, Keffi, Nasarawa state, Nigeria ; ^2^Faculty of Medicine, Ahmadu Bello University Community Medicine, Zaria, Kaduna State, Nigeria

##### **Correspondence:** Abdullahi Hasssan

**Background**

IgE is a monomeric unique homocytotropic molecule that protects individual in Falciparum malaria and Nematodes infection. It is responsible for immune response in hypersensitivity reaction type1 and anaphylaxis.

**Objective**

The aim of this study is to know the levels of IgE antibody in the population under reference.

**Methodology**

The study is a prospective cross sectional studies. IgE serum concentration was determined using double antibody (sandwich) ELISA Technique. Blood collected at the study Centre from the study population and separated to obtained serum following standard protocol. The study subjects recruited were General outpatients, Nonsmokers, non-asthmatic, non-pregnant (if females) and non-staff of the university selected via stratified random sampling technique to obtained 89 clients being the study population.

**Results**

IgE range in the study was found to be between 21iu/ml to 450iu/ml, while normal range was between 21iu/ml to 150iu/ml. 35 (39.3%) of the study population were males of which 20 (57%) have IgE levels <150iu/ml, while 15 (43%) have IgE levels> 150iu/ml. 54 (60.7%) were females, of these females’ clients, 44 (81.5%) have normal IgE levels, while 10 (18.5%) have IgE levels> 150iu/ml. The occupation of the clients under study, revealed that 20(22.5%) were employed and 15 (75%) have IgE levels <150iu/ml while 15 (25%) have IgE levels> 150iu/ml.10(11.2%) were unemployed amongst them, 4 (40%) have IgE levels <150iu/ml and 6 (60%) have IgE levels> 150iu/ml. 21 (23.6%) were self-employed from which 7 (33.3%) have IgE levels <150iu/ml and 14 (66.7%) have IgE levels> 150iu/ml. 38 (42.7%) clients were students, 18 (47.3%) of the students have IgE levels <150iu/ml, while 20 (52.7%) students have IgE levels> 150iu/ml. On clients’ residents, 55 (68.2%) leaves in Samaru where 40 (72.7%) clients amongst them have IgE levels <150iu/ml and 15 (27.3%) have IgE levels> 150iu/ml. 20 (22.5%) clients leave in Palladan where 12 (60%) have IgE levels <150iu/ml while 8 (40%) have IgE levels > 150iu. 14(15.7%) were Giwa residents and 5 (36%) have IgE levels <150iu/ml while 9 (64%) have IgE levels > 150iu/ml.

**Conclusion**

It is being observed that IgE levels has correlation with gender, occupation and place of resident. Knowing IgE levels will help in reducing morbidity and mortality related to allergies.

## A28 Characteristics of serious adverse drug reactions: experience in a single university hospital

### Cheol-Woo Kim^1^, Young Hwa Choi^2^, Jeong Hye Lee^1^, Rae Jeong Cho^1^

#### ^1^Inha University School of Medicine, Incheon, South Korea ; ^2^Ajou University School of Medicine, Suwon, South Korea

##### **Correspondence:** Cheol-Woo Kim

**Background**

The incidence of adverse drug reactions (ADRs) may have been continuously increased as drug use increases. Development of ADRs, especially serious ADRs (SAEs) causes unnecessary patient suffering and may threaten the patient’s life in severe cases. Therefore, efforts should be made to prevent the occurrence of SAEs, which require correct recognition for the characteristics and nature of the SAEs.

**Objectives**

This study was performed for the characterization of SAEs based on spontaneously reported pharmacovigilance database in a single university hospital.

**Methods**

ADRs reported to Inha University Hospital Pharmacovigilance Center were collected from Jan 2012 to Dec 2015, and cases of SAEs were selected. Clinical information was collected from electronic medical records.

**Results**

A total of 495 SAEs (4.9%) among 10,064 ADRs were identified through spontaneous reporting system. Among them, 294 (59.4%) were developed in female patients, and 298 (60.2%) were reported from patients aged 50 and over. SAEs were reported by doctors (41.4%), nurses (24.4%), and pharmacists (34.1%). ADR related-hospitalization or prolongation of existing hospitalization was the most common cause of SAEs, and other medically important event was the second cause. Antineoplastic agents (31.7%), anti-infectives (19.2%), and agents for central nervous system (14.5%) were the drug class commonly involved, and cephalosporin antibiotics and NSAIDs were the leading subclass. Thirty-two cases of severe cutaneous adverse reaction such as Stevens-Johnson syndrome and toxic epidermal necrolysis were detected during study period.

**Conclusion**

Cephalosporin antibiotics and NSAIDs are the most important causative agents that cause SAEs. Hospitalization or extension of the hospital stay are the most common cause of SAEs. Patients with previous history of SAEs or those using high risk drugs need systemic management to control the development of SAEs.

## A29 Spirodela polyrhiza L. extract modulates activation of the atopic dermatitis-related ion channels, Orai1 and TRPV3 and inhibits mast cell degranulation

### Yu Ran Nam, Joo Hyun Nam, Woo Kyung Kim

#### Dongguk University, Physiology, Goyang-Korea, South Korea

##### **Correspondence:** Woo Kyung Kim

**Background**

Increased intracellular calcium in response to T cell receptor/IgE receptor stimulation is a key process in many CD4+ T cell and mast cell functions. In CD4+ T cells, an increase in intracellular Ca2+ concentration ([Ca2+]i) induces Th2 cell differentiation and proliferation and cytokine production through activation of calcium-dependent transcription factors, such as NFAT, and protein kinases, such as PKC. In mast cells, IgE receptor-induced increased [Ca2+]i stimulates exocytosis of histamine-containing granules, activates the production and secretion of leukotriene C4, and increases the synthesis and release of pro-inflammatory cytokines. Therefore, agents that inhibit the calcium channels Orai1 and TRPV3 have therapeutic potential for alleviating inflammatory diseases, including atopic dermatitis.

**Objective**

In this study, we investigated the effects of an extract of Spirodela polyrhiza (Spirodelae Herba, SH) on the modulation of calcium ion channels, Orai-1 and TRPV3 as a potential novel therapeutic for AD and confirmed the regulatory role of these ion channels in mast cell degranulation.

**Methods**

A methanol extract of SH was prepared, and HEK293T cells overexpressing human Orai1 AND TRPV3 were treated with the extract. Modulation of the ion channels in RBL-2H3 cells was measured using a conventional whole-cell patch-clamp technique. IgE-antigen complex-stimulated mast cell degranulation was measured using a beta-hexosaminidase activity assay along with morphological observation.

**Results**

SH extract (100 μg/mL) significantly inhibited Orai1 activity in Orai1-STIM1 co-overexpressing HEK293T cells. In addition, HS extract significantly increased TRPV3 activity compared to that in the presence of 2-APB (100 μM), which induces full activation. SH extract (20, 50, and 100 μg/mL) inhibited degranulation in IgE-antigen complex-stimulated RBL-2H3 cells as measured by a decrease in β-hexosaminidase activity.

**Conclusions**

Our results suggest that SH extract has potential for the treatment of abnormal skin barrier pathologies in atopic dermatitis by modulating the activities of the calcium ion channels Orai1 and TRPV3 and inhibiting mast cell degranulation. To the best of our knowledge, this is the first report on an herbal effect on the modulation of ion channels associated with skin barrier disruption in atopic dermatitis pathogenesis.

## A30 Allergen specific immunotherapy: effect on immunological markers and clinical outcomes in asthmatic children - a real life clinical trial

### Ivana Filipovic^1^, Zorica Zivkovic ^2^, Djordje Filipovic ^3^

#### ^1^Faculty of Medical Science Kragujevac-Immunology, Kragujevac, Serbia ; ^2^Children’s Hospital for Lung Diseases and Tuberculosis- Medical Center Dr Dragisa Misovic-Pediatric, Belgrade, Serbia ; ^3^Institution for Emergency Medical Care Anesthesiology, Belgrade, Serbia

##### **Correspondence:** Ivana Filipovic

**Background**

Allergen-specific immunotherapy (AIT) holds a great promise in the management of allergic conditions, as the only with the capability to change the natural causes of allergic diseases. Most studies evaluated clinical scores as the main parameters, whereas immunological and-or inflammatory factors were studied only occasionally.

The aim of this study was to investigate the efficacy of SLIT on FeNO, as well as to compare the relationship to asthma symptom and medication score and parameters of lung function.

**Methods**

59 asthmatic children were included in the study. 34 patients were received sublingual allergen specific immunotherapy (SLIT) plus standard pharmacotherapy while 29 children received only standard pharmacotherapy according to the GINA guideline. Efficacy was evaluated using FeNo, asthma and medication symptom score and lung function tests. The results of the test were compared at baseline, during the first year of follow up period and at the end of the observational period.

**Results**

FeNO values decreased significantly in SLIT group (X2=52,220; *p*<0,001) compared to baseline during the first and the second year of follow up period, whereas control group values remained similar. The SLIT group experienced significant improvement in asthma symptoms and medication score, whereas the control group did not. Lung function tests were also changed significantly but only in the SLIT group.

**Conclusions**

SLIT improves biological and clinical parameters in asthmatic children during the first year of follow up period with a sustainable improvement during the second year. This is one of the first studies found positive correlation between subjective parameters such as asthma and medication score and objective parameter FeNO and lung function.

## A31 Induction of IL-9-producing Mucosal Mast Cells (MMC9s) contributes to the comorbidity of allergic diseases

### Dana Shik, Andrew Smith, Wang Yui Hsi

#### CCHMC Allergy and Immunology, Cincinnati, OH, United States

##### **Correspondence:** Dana Shik

**Background**

Clinical studies reveal that certain individuals with atopic dermatitis (AD) in early life are prone to develop food allergy and asthma, also known as atopic march. However, the underlying mechanisms that contribute to the comorbidity of allergic disorders remain unclear.

**Objective**

Determine the role of MMC9s in co-morbid allergic diseases and the effect of IL-33 on MMC9 accumulation and function.

**Methods**

To establish an experimental atopic march model, mice that develop AD after vitamin D3 analog treatments were inoculated with ingested antigens before subjecting to intranasal allergen challenges. The roles of IL-33/ST2 signals in regulating the progression of allergic diseases were examined using genetically-modified mice. Duodenal biopsies from atopic patients who were tested negative (control) or positive for food allergy (FA) and additional allergic disorders were procured to compare their IL-9 and IL-13 production after IL-33 in vitro stimulation.

**Results**

We have recently identified the novel IL-9-producing mucosal mast cells (MMC9s) which can secrete prodigious amounts of IL-9 and mast cell (MC) mediators to promote IgE-mediated food allergy. Epidermal thymic stroma lymphopoietin (TSLP) induction after skin sensitizations induce atopic dermatitis in mice (AD-like mice). Repeated intragastric inoculations with ovalbumin (OVA), but not saline, induced MMC9 accumulation in the gut of AD-like mice which eventually developed symptoms of experimental food allergy. Notably, intranasal OVA challenge triggered significant leukocyte infiltrations and IL-4 and TARC production in the bronchoalveolar lavage fluid in AD-like mice inoculated intragastrically with OVA but not saline. Flow cytometric analysis reveals that intranasal OVA challenge induced an increase of pulmonary MMC9s, but a decrease of intestinal MMC9s in AD-like mice that had developed experimental food allergy. Interestingly, after skin sensitization, mice deficient of IL-33 receptor (St2) failed to produce intestinal MMC9s, resulting in the resistance to develop experimental food allergy and airway inflammation after intranasal OVA challenge. In duodenal biopsies from comorbid allergic disease (CAD) patients, MMC9 occurrence and IL-33-stimulated IL-9 and IL-13 production were significantly higher than in control individuals.

**Conclusions**

Our results suggest that MMC9 induction in the gut of AD-like mice may perpetuate the progression of allergic diseases and that IL-33/ST2 signals may amplify MMC9 function and airway MMC9 accumulation to promote airway inflammation. Thus, MMC9 may serve as a key cellular checkpoint that bridges epidermal TSLP production to asthma development and contributes to the co-morbidity and severity of allergic disorders.

## A32 Another case of acquired angioedema secondary to a lymphoproliferative disease successfully treated with Rituximab

### Stuart Friedman^1^, Yonatan Gizaw^2^, Rima Bakhda^2^, Kumail Mohammed^2^

#### ^1^Schmidt College of Medicine Florida Atlantic University Clinical Integrative Medicine, Boca Raton, FL, United States ; ^2^Boca Raton Regional Hospital Medicine, Boca Raton, FL, United States

##### **Correspondence:** Stuart Friedman

**Background**

There are fewer than 150 reported cases of acquired angioedema. There are only a few cases with documented successful treatment with rituximab. We have added to the literature another case with documentation of the clinical presentation as well as documented response to the treatment with rituximab.

**Objective**

To report the presentation, evaluation and management of an 84 year old female with recurrent severe oropharyngeal angioedema. To add to the literature that demonstrates that rituximab, by mediating the lysis of B-cells can be effective as a treatment for lymphoproliferative disorder associated acquired angioedema.

**Methods**

The patient is cared for by SF. Medical records were reviewed and laboratory tests were obtained at our hospital. Treatment with rituximab was rendered at our hospital’s cancer center.

**Results**

C1 esterase inhibitor functional and quantitative were reduced as were C1q and C4. Evaluation revealed an enlarged spleen and enlarged peri-portal and portcaval lymph nodes. Flow cytometry was compatible with a low grade splenic mantle cell lymphoma. Treatment was rendered with rituximab. C1 esterase inhibitor values improved and there has not been any recurrence of angioedema.

**Conclusions**

Patients presenting with angioedema accompanied by low/absent C1 esterase levels should be evaluated for lymphoproliferative autoimmune or disorders. If these types of disorders are found, rituximab treatment should be considered. We have added to the literature with an additional case of acquired angioedema responding to treatment with rituximab.

## A33 Single practice eight-year experience treating food allergy with oral immunotherapy

### Richard Wasserman^1^, Angela Hague^1^, Deanna Pence^1^, Joanna Rolen^1^, Robert Sugerman^1^, Stacy Silvers^1^, Qurat Kamili^2^

#### ^1^Medical City Dallas Hospital Pediatrics, Dallas, TX, United States ; ^2^Medical City Dallas Hospital Medicine, Dallas, TX, United States

##### **Correspondence:** Richard Wasserman

**Background**

The prevalence of food allergy, a socially debilitating and potentially life-threatening disorder, has increased significantly over the past ten years. Patients and families are avidly seeking treatment but there are few options. Consequently, interest in FOIT (food oral immunotherapy) in the private practice setting has risen commensurately.

**Objective**

To assess the experiences and treatment outcomes of 481 FOIT treated patients, 343 who reached their target dose and 75 who did not. Partial data on 63 patients who were dose escalating when the data collection was closed is included as well.

Methods

A retrospective record review of patients initiating FOIT from 6/10/08 to 6/1/16 was approved by the North Texas IRB. Patients received FOIT doses beginning with <5mcg of nut or 100mcg of other food protein with target protein doses of 3600mg (egg white) to 8000mg (wheat) and 10,826mg (four nuts).

**Results**

Patients were treated with FOIT if they had a clear history of allergic reaction and supportive skin prick testing or specific IgE. Most patients with an equivocal history or testing were challenged to prove food allergy. Patients whose specific IgE suggested that food challenge would be inappropriately unsafe were treated without a prior confirmatory challenge. No patient was excluded because of a history of a serious reaction or a high specific IgE.

Eighty percent of patients reached their target dose and began maintenance. Egg treated patients were more likely to reach the target (94%) than milk (78%) or peanut (79%) treated patients. Patients discontinued peanut FOIT primarily because of EoE-like OIT related gastrointestinal syndrome (ELOGS) or systemic reactions; 6.3% and 4.7% respectively. Among peanut treated patients who reached maintenance, 18 (9.4%) later discontinued treatment, 7 because of taste aversion. Of the milk treated patients who didn’t reach or couldn’t be confirmed to have reached the target, 8 (44%) were lost to follow up, 3 (17%) discontinued because of reactions and 4 (22%) because of ELOGS.

**Conclusions**

Consistently, over the eight-year experience with FOIT, approximately 80% of patients reach their target dose. FOIT can be successfully performed in an appropriately prepared allergy office.

## A34 Evaluation of PD-1 expression on T-lymphocytes in allergic rhinitis

### Nadezhda Knauer^1^, Alexandr Zazernyi^2^, Elena Blinova^1^, Daria Demina^3^, Vladimir Kozlov^1^

#### ^1^Research Institute of Fundamental and Clinical Immunology, Laboratory of Clinical Immunopathology, Novosibirsk, Russia ; ^2^ Novosibirsk State Medical University Pediatric Faculty, Novosibirsk, Russia ; ^3^ Research Institute of Fundamental and Clinical Immunology, Allergology Department of the Clinic of Immunopathology, Novosibirsk, Russia

##### **Correspondence:** Nadezhda Knauer

**Background**

According to the recent reports, pathogenesis of allergic diseases includes either increasing of the number of the activated effector cells or decreasing of the number of T-regulatory cells (Tregs) and the reduction of their functional activity.

**Objective**

The evaluation of the expression of the activation markers on T-lymphocytes, such as PD-1 and CD25, as well as evaluation the number of Tregs, in healthy volunteers and patients with allergy is important for understanding some aspects of allergy pathogenesis.

**Methods**

10 healthy volunteers (age 23±4.8) and 11 patients with allergic rhinitis (AR) with sensitization to birch pollen allergens or house dust allergens (age 28±7.8) were included in the study. The sensitization was confirmed by skin prick tests. Peripheral blood mononuclear cells (PBMCs) were extracted from heparinized blood, then stained by fluorescently labelled monoclonal antibodies. The number of cells in each population (CD4+CD25+, CD8+CD25+, CD4+CD25high, CD8+CD25high, CD4+PD-1+, CD8+PD-1+, CD4+CD25highPD-1+, CD8+CD25highPD-1+) were evaluated by flow cytometry. We defined CD4+CD25high as Tregs, and cells expressing markers PD-1 or CD25 as activated cells. Statistical analysis was made using Mann-Whitney criterion, the difference was considered significant if p<0.05.

**Results**

We have found the significant decreasing of Tregs (CD4+CD25high) number in patients group. Moreover the density of CD25-expression on Tregs was significantly lower in this group. The number of CD4+PD-1+ lymphocytes in volunteers group was significantly lower than in patients with AR. We have found the same tendency for activated T-lymphocytes CD8+PD-1+. The significant difference of PD-1-expression on Tregs between group of donors and patients group was not found.

**Conclusions**

We have found that number of lymphocytes with PD-1, which can be considered to be the marker of activation, is increased in patients with AR in comparison with healthy donors – significantly in case of CD4+-T-lymphocytes and as a tendency in case of CD8+-T-lymphocytes. In the same time, the number of Tregs decreased in patients group. Moreover, the expression of CD25 on Tregs was lower than in donors group. These results can confirm the theory that allergic diseases can be associated not only with increasing the number of activated effector cells, especially, T-helpers, but also with the reduction of Tregs-subpopulation and, probably, their functional dysfunctions.

## A35 Per a 10 favours Th2 responses by PAR-2 activation and increased p-STAT 3 levels

### Komal Agrawal, Sagar Kale, Naveen Arora

#### CSIR-Institute of Genomics and Integrative Biology Allergy and Immunology section, Delhi, India

##### **Correspondence:** Komal Agrawal

**Background**

Per a 10, a major serine protease allergen from Periplaneta americana favours Th2 responses by activating dendritic cells to higher IL-23 and secrete lower IL-12p70.

**Objective**

The present study is aimed to elucidate the role of proteases in regulating the balance between members of IL-12 cytokine family.

**Methods**

Female Balb/c mice were administered Per a 10, heat-inactivated Per a 10 (Δ Per a 10) or PBS on days 0,2,4,10,12,14 and sacrificed on 15th day. PAR-2 activation in Per a 10 sensitized mice was blocked by administering PAR-2 cleavage blocking antibody via intranasal route 30 min before sensitization. Cytokines, total cell count in BALF and specific IgE and IgG1 levels in sera were analysed. The mRNA levels of cytokine subunits and p-STAT3 were analysed in lungs by RT PCR and Western blot. PAR-2 activation on BMDCs was blocked using antibody and stimulated with Per a 10. p-STAT3 and cytokines level were measured using flow cytometry and ELISA respectively.

**Results**

Per a 10 administration leads to higher levels of p-STAT3 in lungs of mice as compared to proteolytically inactive counterpart. IL-4, IL-17A, TSLP, specific IgE and IgG1 levels, eosinophil peroxidise activity and total cell count were lower in SAM-11 administered group as compared to isotype control indicating role of PAR-2 signalling in Th2 responses. IL-12p35 and IL-12p70 levels were higher and IL-23p19 and IL-23 levels were lower in SAM11 administered group as compared to isotype control pointing a role of PAR-2 activation in transcriptional regulation of cytokine subunits. There was no change in IL-12/23p40 transcript levels. p-STAT3 levels were lower in the lungs of SAM11 administered mice. SAM-11 treated BMDCs lead to lower p-STAT3 levels as compared to isotype treated BMDCs. Further, Per a 10 stimulation in SAM-11 treated BMDCs showed higher IL-12p35 and lower IL-23p19 at transcript level as compared to isotype control. Cytokine analysis in supernatant revealed higher IL-12p70 and lower IL-23 levels indicating role of PAR2 mediated signalling in the secretion of IL-23 and IL-12p70 by dendritic cells.

**Conclusions**

Per a 10 regulates the balance between IL-12p70 and IL-23 by activating PAR-2 and STAT3 in dendritic cells.

## A36 Association of C-reactive protein and interleukin-6 with chronic kidney disease in patients with diabetes type 2

### Volha Vasilkova^1^, Tatiana Mokhort^2^

#### ^1^Gomel State Medical University Endocrinology, Gomel, Belarus ; ^2^ Belarusian State Medical University Endocrinology, Minsk, Belarus

##### **Correspondence:** Volha Vasilkova

**Background**

Background: Chronic kidney disease (CKD) is a serious complication of diabetes associated with increased risk of mortality, cardiovascular and renal outcomes [1]. Systemic inflammation has been defined as a marker of cardiovascular risk in patients with CKD [2].

**Objective**

Aim of our study was to evaluate the association of inflammatory biomarkers including C-reactive protein (CRP) and interleukin-6 (IL-6) with CKD in patients with diabetes type 2 (DT2).

**Methods**

Seventy eight patients both sexes with type 2 diabetes aged 54.69±11.07 years were studied. Control group included 37 subjects the same age. CKD was defined as eGFR <60 ml/min/1.73 m2 or the presence of albuminuria (≥30 mg/24-h) for three or more months. Multivariate regression analysis was used to examine associations between the inflammatory biomarkers and CKD adjusting for CKD risk factors.

**Results**

Patients with CKD and DT2 had a mean eGFR of 46.2 mL/min/1.73 m2 compared to 95.3 mL/min/1.73 m2 among controls without CKD and DT2. The median serum levels of CRP and IL-6 were significantly higher in patients with CKD and DT2 compared to controls (*p*<0.05). After adjusting for important covariables, the median of IL-6, but not CRP, remained significantly higher. In multivariate linear regression analyses, serum level of IL-6 was inversely and significantly associated with eGFR (−4.89; −7.75 to −1.63; *p*<0.001) and positively related to urinary albumin excretion (0.24; 0.04 to 0.43; *p*=0.0004).

**Conclusions**

Our data suggest that IL-6 is associated with the prevalence of CKD in patients with DT2, independent from established CKD risk factors.

References

1. Levey AS, Coresh J. Chronic kidney disease. Lancet. 2012; 379:165–80.

2. Belinda T. Lee, Faheemuddin A. Ahmed, L. Lee Hamm, Federico J. Teran et al. Association of C-reactive protein, tumor necrosis factor-alpha, and interleukin-6 with chronic kidney disease. BMC Nephrology. 2015; 16:77.

## A37 Legalized marijuana- medical and recreational- in the USA: concerns beyond cannabis sativa?

### William Silvers

#### University of Colorado School of Medicine Medicine GWV, Aurora, CO, United States

**Background**

Since the passage of legalized medical marijuana (MJ) in the USA (now 26 states plus District of Columbia) and recreational marijuana (now 4 states, initially Colorado in 2014), an increasing number of allergic patients are presenting due to MJ exposure. The majority have significant exposure in the grow industry or heavy consumers, indicating that cannabis sativa is a mild allergen. Some highly atopic patients have reactions with less exposure. However, the techniques for growth and extraction are changing, and factors other than the weed allergen itself may be responsible for adverse reactions.

**Objective**

Describe a spectrum of reactions.

**Case reports**

Case 1. A 28 y/o white male presented with rhinitis worsening around “pot” in 2010. When he began working as a “trimmer” at a growth house in 2014, symptoms flared and included wheezing and chronic cough. He improved with nasal steroids and bronchodilator use.

Case 2. A 30 y/o white male presented with AR, conjunctivitis, contact dermatitis, and asthma symptoms after MJ exposure, having no previous asthma history. He moved to Colorado to work in the MJ industry, initially as a grower and then retail. He grew his own MJ plants. FEV1 was 52% predicted with 38% bronchodilator improvement. FeNO was elevated at 89 ppb, skin tests had multiple positives to pollen, and “puddle” tests were positive to MJ. After treatment, his FEV1 improved to 80% predicted, FeNO decreased to 30 ppb, and symptoms improved.

Case 3. A 26 y/o white male was referred by an emergency physician with suspected anaphylaxis after smoking MJ, and subsequently with passive exposure. He described using a “concentrator wax” on top of the MJ flower. Immunocap tests were negative to pollens, but positive to danders, and dust mites. With a negative MJ flower “puddle” test, consideration was given to other potential triggers, such as pesticides (now outlawed for inhaled and edible MJ production in Colorado), mold, dust mite, psychophysiologic reactions, or other unknown factors in the waxes. Anecdotes: During a marijuana “bust” in September, 2015, 2 Drug Enforcement Agency agents presented with asthma exacerbations to the MJ brought into their offices, despite one being on omalizumab. The other had a history of reactions to “strain specific” marijuana exposure.

**Conclusion**

Although cannabis sativa may be a mild allergen for most, increasing workplace exposure and

**Consent**

The author received informed consent from the patients to publish.

## A38 Management of refractory recurrent pericarditis and Familial Mediterranean Fever (FMF) related attacks with IVIG and interleukin 1 inhibition

### Rachel Eisenberg, Rushita Mehta, Arye Rubinstein

#### Montefiore Medical Center Allergy and Immunology, Bronx, New York, United States

##### **Correspondence:** Rachel Eisenberg

**Background**

Familial Mediterranean fever (FMF) is an autosomal recessive autoinflammatory disorder characterized by episodes of fever, gastrointestinal (GI) serositis, and other findings including arthritis, pericarditis, skin lesions, and myalgias. Recommended treatment for both FMF and recurrent pericarditis is colchicine. We present a 32 year old female with uncontrolled FMF and chronic pericarditis on colchicine who clinically improved with intravenous gammaglobulin (IVIG) along with abortive and preventive anti IL1-beta biologicals.

**Case report**

A 32 year old female presented with nausea, vomiting, abdominal cramps, and chills since adolescence, generally occurring once every 2-8 weeks. She also had intermittent knee and wrist pain, as well as brown skin lesions overlying her ankles. During her first pregnancy her abdominal symptoms worsened leading to her diagnosis of FMF by genetic testing (V726A mutation). Fat pad biopsy was negative for amyloidosis. Despite treatment with colchicine she continued to have nausea and GI serositis 1-3 times monthly. Additionally, the patient had a 12-year history of refractory recurrent constrictive pericarditis unresponsive to colchicine and steroids. She required cardiocentesis yielding 450ml of pericardial fluid. Immunologic workup was notable for elevated Serum IL-1β levels at 69.6 (normal <3.99 pg/ml) and hypogammaglobulinemia. High dose IVIG and abortive anakinra therapy was initiated. Over the course of a year on IVIG she reported decreased frequency and severity of recurrences. She had two mild recurrences of pericarditis and one episode of GI serositis both of which responded promptly to 3 doses of subcutaneous anakinra. She then developed a hypersensitivity reaction to anakinra with hives and pruritus. Skin prick and intradermal testing to anakinra was positive. Anakinra was discontinued with plan to treat recurrences with abortive rilonacept. She had one episode of GI serositis and chest pain since then, treated and responsive to one dose of rilonacept. She was subsequently maintained on rilonacept prophylaxis every other week, without recurrences of abdominal attacks or chest pain for the past five months.

**Conclusion**

Currently, colchicine is the first line therapy and only proven effective treatment for FMF flares and recurrent pericarditis. We present a case refractory to these drugs, whose symptoms of FMF and pericarditis responded to high-dose IVIG and to anti IL1 abortive and prophylactic treatments. The combination of IVIG and IL1-beta receptor inhibition may be a superior therapy for FMF and associated pericarditis. Whether it will have a benefit in prevention of amyloidosis has yet to be determined.

**Consent**

The authors received informed consent from the patient to publish.

## A39 Assessing severity of peanut-allergic reactions during research food challenges

### Antony Aston^1^, Paul Turner^1^, Monica Ruiz-Garcia^1^, Robert Boyle^1^, Simon Brown^2^

#### ^1^Imperial College London Paediatric Allergy & Immunology, London, United Kingdom ; ^2^University of Western Australia Emergency Medicine, Perth, Australia

##### **Correspondence:** Antony Aston

**Background**

Determining the severity of allergic reactions with a valid and discriminatory scoring system has important implications for research. Existing grading systems do not offer sufficient discrimination to facilitate research into the mechanisms of severity, immunotherapy outcomes where only partial tolerance is induced, and allergen risk management.

**Objective**

To develop and validate a severity score for food-allergic reactions.

**Methods**

We developed an in-house scoring system, utilising PRACTALL criteria, with validation against inflammatory mediator measurements of patients experiencing anaphylaxis in a cohort of patients with anaphylaxis presenting to Emergency Departments in Australia (Brown et al., JACI 2013;132:1141-1149). We then sought to refine and further validate the score using symptom data collected prospectively in two cohorts of peanut-allergic individuals undergoing DBPCFC to peanut. The severity of reaction was also graded according to several existing schemata in the literature (Ewan & Clark (2001), Sampson (2003), Hourihane (2005), Niggemann & Beyer (2016)). Clinicaltrials.gov registration NCT02149719 and NCT02665793; informed consent was obtained prior to challenge.

**Results**

One hundred fift gao 105 participants (adults, *n*=60; children, *n*=45) underwent DBPCFC, resulting in anaphylaxis (NIAID criteria) in 29 (27%) cases and less severe symptoms in the remainder. The scoring system resulted in a normal distribution of reaction severity in the cohort, by D’Agostino & Pearson normality test, confirming good discrimination between reactions of differing severity compared to traditional grading systems, and allowing a better assessment of the relationship between inflammatory mediators such as mast cell tryptase and reaction severity.

**Conclusion**

The proposed severity score provides good discrimination and normal distribution of food-allergic reactions for the research setting. The tool is currently being applied to data from ongoing studies of anaphylaxis pathophysiology and oral immunotherapy.

## A40 Hematopoietic stem cell transplantation in combined immunodeficiency associated with RelB deficiency

### Yael Dinur Schejter, Adi Ovadia, Vy Kim, Brenda Reid, Chaim Roifman

#### Hospital for Sick Children, Allergy and Immunology, Toronto, Canada

##### **Correspondence:** Yael Dinur Schejter

**Background**

We report for the first experience with hematopoietic stem cell transplantation (HSCT) for RelB deficiency, a new form of combined immunodeficiency (CID). RelB is a key mediator of the alternative pathway of the NFĸB family of transcription factors.

**Methods**

Two patients previously described (Merico et al. 2015; Sharfe et al. 2015) with CID associated with RelB deficiency underwent an HSCT from a 10/10 matched unrelated donors, using busulfan and cyclophosphamide conditioning regimen, and graft versus host (GVHD) prophylaxis with methylprednisone and cyclosporin A (CSA). Patient data was compiled retrospectively from medical records and entered into the Canadian Centre for Primary Immunodeficiency Registry. Follow up period was 2.5-4 years.

**Results**

Patient 1 suffered from recurrent otitis media (OM) and pneumoniae, ecthyma gangrenosum, disseminated HSV infection, adenoviral infection and intermittent bilateral knee swelling. Patient 2, first cousin of patient 1, suffered from recurrent OM, perinatal pneumothorax, pneumonia, a urinary tract infection, oral thrush, chronic cough and failure to thrive. Both patients had a profound B and T cell impairment with low in vitro T cell responses to mitogen stimulation, poor response to specific antibodies, dysplastic thymus and low T cell excision circles (TREC) values.

Patient 1 underwent HSCT at the age of two years. Engraftment was rapid and post transplant course was unremarkable except for one episode of Staphylococcus aureus infection as well as HSV1 skin infections. CSA prophylaxis was switched to mycophenolate mofetil due to nephrotoxicity, which subsequently resolved. He developed Grade I skin GVHD on day +13 and was treated with a pulse of methylprednisolone with resolution of symptoms, but subsequently developed chronic skin GVHD 18 months after transplant. He was initially treated topically but was later on started on methotrexate therapy due to worsening of his skin GVHD, with good response. He is currently 4 years post transplant and has full donor chimerism, but continues to suffer chronic skin GVHD. Patient 2 was transplanted at the age of 3 years. Engraftment was rapid and his post transplant course was uneventful initially, but later on he suffered 1 episode of OM and pneumonia. He did not develop GVHD. He is currently well 2.5 years post transplant. Chimerism studies show a stable donor T cell engraftment at 60% of total T cells. T cell mitogen responses are present and immunoglobulin levels are normal, off intravenous immunoglobulin therapy.

**Conclusion**

HSCT can reverse combined immune deficiency in RelB deficient patients.

**Consent**

The authors received informed consent from the patients’ guardian/parent to publish.

## A41 Dining out: a survey of experiences in Canadians with food allergies

### Lana Rosenfield^1^, Ernie Avilla^2^, Laurie Harada^3^, Marilyn Allen^3^, Susan Waserman^1^

#### ^1^McMaster University Division of Clinical Immunology and Allergy, Hamilton, Canada ; ^2^McMaster University Department of Medicine, Hamilton, Canada ; ^3^Food Allergy Canada, Toronto, Canada

##### **Correspondence:** Lana Rosenfield

**Background**

Dining out poses a risk for food allergic individuals. Potential explanations include cross contamination with food allergens or inadequate information on ingredients. This risk is increased when individuals do not carry an Epinephrine Auto-injector (EAI) when dining-out, or fail to use it when indicated. We evaluated the experience of food allergic individuals in Canada when dining out and their perceptions of risk reduction strategies such as stock EAI.

**Methods**

We conducted a cross sectional study to evaluate the dining-out experiences of food allergic individuals. Online surveys were administered to adults (>18 years of age) with food allergy and parents/caregivers of children (<18 years of age) with food allergy from Food Allergy Canada’s database in February 2015.

**Results**

Population: Of 1,580 respondents, we included 1165 fully completed surveys in the analysis. 23.9% were food allergic, 66.1% had a child with food allergy and 10% had another family member with food allergy.

Carriage of EAIs: When dining out, 87.8% of respondents stated that they ‘always’ carried, 2.3% ‘never’ carried, 4.4% ‘usually’ carried, and 1.5% ‘sometimes’ carried an EAI (4% did not provide an answer to this question).

Dining out experience: 1096 of 1165 (94%) respondents indicated that they dine out, while 3% said they do not. 53% who dine out would check a restaurant website to assess for potential allergens, 46% will call ahead, 95% will speak to restaurant staff. Only 2% will not tell restaurant staff about their allergies. Of 1096 who dine out, 33% have experienced an allergic reaction while dining-out (43% adults; 46% children, 11% other family member). Prior to the reaction, 22.3% informed staff of food allergy, 10% spoke to the chef, 4.3% called the restaurant in advance, and 3% presented a list of allergies.

Perception of stock EAI: Overall, 87.5% respondents would continue to carry an EAI if there was stock EAI at a restaurant. 44.5% would have increased comfort dining out if EAI was present on the premises.

**Conclusion**

The survey demonstrates a significant rate (1 in 3) of allergic reactions in food allergic individuals while dining out, this is spite of asking about relevant food allergens. It also emphasizes the importance of always carrying an EAI, not practiced by 12% of respondents. While the majority would carry EAI even with stock EAI in a restaurant, half would feel more comfortable with it present.

## A42 Association of serum triglyceride and cholesterol with asthma in adults

### Ho Joo Yoon ^1^, Gun Woo Koo^1^, Suk-Il Chang^2^, Hye-Ran Yoon ^3^, Dong Won Park ^1^, Tai Sun Park^1^, Ji Yong Moon ^1^, Sang-Heon Kim ^1^, Tae Hyung Kim ^1^, Jang Won Sohn ^1^, Dong Ho Shin ^1^

#### ^1^Hanyang University College of Medicine Department of Internal Medicine, Seoul, South Korea; ^2^Sungae General Hospital Department of Internal Medicine, Seoul, South Korea; ^3^Duksung Women’s University College of Pharmacy, Seoul, South Korea

##### **Correspondence:** Ho Joo Yoon

**Background**

Dyslipidemia is a risk factor for cardiovascular diseases by affecting immune and inflammatory reactions. Previous studies suggested that dyslipidemia is closely associated with asthma and atopy, while the results are not consistent.

**Objective**

In this study, we aimed to examine the possible association of dyslipidemia with asthma by analyzing data from Korean National Health and Nutrition Examination Survey (KNHANES) in Korean adults.

**Methods**

We performed cross-sectional study of adults aged ≥19 from KNHANES 2010. Asthma outcome was obtained by standardized questionnaire from the participants. Serum levels of triglycerides (TG), total cholesterol (TC), LDL and HDL cholesterols were compared between asthma and non-asthma. Multivariate logistic regression analysis was performed to explore the association of dyslipidemia and asthma.

**Results**

Serum level of TG was higher in subjects with asthma than non-asthma, while there were no significant difference between levels of TC, LDL and HDL cholesterol. In multivariate analysis adjusting for age, sex, body mass index, education level and household income, serum level of TG showed significant inverse relationship with asthma outcomes: asthma ever (adjusted OR 0.76, 95% CI 0.62-0.91, *P* = 0.004) and diagnosed asthma (adjusted OR 0.67, 95% CI 0.52-0.86, *P* = 0.002).

**Conclusions**

In adult population of Korea, serum TG is inversely related with asthma. There is no close relationship between serum level of cholesterol (TC, LDL and HDL cholesterol) with asthma.

## A43 Complex evaluation of the health status of primary-school aged children (Adjara region)

### Tsici Jorjoliani^1^, Lia Jorjoliani^2^, Nino Adamia^3^, ^1^

#### ^1^D.aghmashenebeli University Pediatric, Tbilisi, Georgia; ^2^I.Javakhishvili Tbilisi Steit University Pediatric, Tbilisi, Georgia; ^3^Tbilisi State Medical University Pediatric, Tbilisi, Georgia

##### **Correspondence:** Tsici Jorjoliani

Goal of the research was complex evaluation of the health status of the primary-school aged children residing in various regions (urban, rural) of Adjaria.

Cross-section, one-stage research was conducted in the City of Batumi and village Tsikhisdziri. In the process of survey health status of children of 4 public schools, from 6 to 9 years old was studied. Observations covered up to 800 school children in total. Screening included consultations of the multidisciplinary group of specialists, additional laboratory and instrumental studies intended for the purpose of accurate diagnostics.

Performed studied showed that 28.3% of the studied population was actually healthy, 55% had functional disorders and 16.7% - chronic diseases. In both, urban and rural areas the share of the digestion system, blood and blood-generating organs, nervous system, ophthalmological pathologies and locomotion system diseases prevailed.

## A44 Study on hand washing practices and occurrence of Microbes on the hands of food handlers.

### Deepika Ramachandra

#### BMCRI Immunology, Bangalore, India

**Background**

Food safety has emerged as an important global issue with international trade and public health implications. Studies revealed that food borne diseases are a serious health hazard and an important cause of morbidity and mortality in developing countries. Food can transmit a wide range of diseases in a condition termed food infection where food serves as a vehicle for transfer of pathogens to the consumer in whom the pathogen grow and cause disease. Data on risk factors for food borne diseases indicate that the majority of outbreaks result from inappropriate food handling practices. Food handlers with poor hygiene, working in food serving establishments could be potential source of infections of many enteric pathogenic bacteria. There is evidence from the food industry showing that microorganism are transferred to the hands in the process of handling food and through poor personal hygiene, resulting in the hands being heavily contaminated with enteric pathogens. Hand washing, a simple and effective way to cut down on cross contamination is all too often forgotten. In addition, if individuals do not wash their hands before putting on the gloves, both the interior and exterior gets contaminated and microorganisms on the hand could multiply rapidly in the moist and warm environment

**Objective**

1. To isolate and identify the organism from hands of food handlers in hospital and private eateries before and after the hand wash

2.To demonstrate appropriate hand washing technique to the food handlers.

3.To evaluate the effect of hand washing technique which is a simple and cost effective method of disinfection.

4.To study and compare any difference in the isolated microbes from the two groups

of food handlers i.e. hospital and private eateries.

**Methods**

Sample size: *n*1-50; staff working in hospital kitchen, hostel kitchen and canteen: *n*2-50; staff working in private eateries around the hospital. Informed consent shall be obtained from each subject included in the study, samples will be taken from the palm of the food handlers from index fingers and thumb of both right and left hands using a sterile swab wetted with normal saline. A simple 13 seconds hand wash whereby the hands were soaped lathered and during lathering rinsed underneath a faucet of flowing water. Aerobic cultures will be done on Mac Conkey’s Agar and Blood Agar, and shall be incubated at 370C, for 18 hours. If there is growth, the total viable count shall be determined. Antibiotic sensitivity testing will be done using Standard Kirby Bauer’s method. The results obtained will be statistically analyzed and evaluated.

## A45 Food Allergy

### Liana Jorjoliani, Rusudan Karseladze, Lali Saginadze, Nino Adamia, Natalia Chkuaseli

#### Tbilisi State Medical University, Department of Pediatrics, Tbilisi, Georgia

##### **Correspondence:** Liana Jorjoliani

**Objective**

To investigate the prevalence of food allergies in the population of preschool children from the city of Batumi and to identify the most common causes of the allergies in the study population

**Methods**

The study was conducted in children population, in Batumi, according to the random and representative sample, by the cross section method of epidemiological survey. On the first stage of the survey 840 children aged from 6 months to 7 years, (50.6% girls and 49.4% - boys) were questioned by random selection. Analyzed using SPSS Statistical Package for Windows, version 13.0

**Results**

According to study results, for last 12 months 6.1% of the studied children had signs of allergies. According to clinical-allergic study, allergic rash – in kind of dermatitis and urticaria (76%) was significantly (p<0.05) higher than manifestation rate of gastrointestinal symptoms (24%). The most common causes of food allergies in the study population were eggs (22.3%), fish (22.6%), milk (18.6%), honey (13.7%;), nut (4.3%) and different food additives.

**Conclusions**

Therefore, in childhood allergic skin manifestation of food allergy is high. According to the obtained data risk factor control could provide basis for the purposeful and effective preventive measures in future. There is a need to work on educating of parents and caregivers.

## A46 Micro-array component-resolved study of Felis domestica major allergen molecules in cat allergic patients in Moscow region, Russia

### Anna Dolgova^1^, Olga Stukolova^1^, Anna Sudina^1^, Anna Cherkashina^1^, German Shipulin ^1^

#### ^1^Central Research Institute for Epidemiology, Federal Service on Consumers’ Rights Protection and Human Well-Being Surveillance Molecular Diagnostics and Epidemiology, Moscow, Russia

##### **Correspondence:** Anna Dolgova

**Background**

In Russia, 37% of households have cats and cat allergy frequently occurs. However there is still no information about IgE reactivity to individual cat allergens in this region.

**Objective**

We aimed to determine the profile of IgE reactivity to three major cat allergens Fel d1, Fel d 2 and Fel d 4 in cat allergic patients in Moscow region, Russia.

**Methods**

The study was conducted using sera from 230 anonymous subjects: 174 patients with ≥ 0.35 IU/mL serum levels of IgE to cat dander extract (e1, ImmunoCAP) and 56 negative controls. Allergens were expressed as recombinant proteins in Escherichia coli (Fel d 1 and Fel d 4) or purified from cat serum (Fel d 2). IgE levels were measured using microarray method.

**Results**

Fel d 1 was found to bind IgE from 91.4%, Fel d 2 from 33.3% and Fel d 4 from 49.4% of the patients’ sera tested. Mean IgE reactivity to Fel d 1 was significantly higher than to Fel d 2 and Fel d 4. We found a moderate positive correlation between total IgE and specific IgE to Fel d 1 and low positive correlation between total IgE and Fel d 2 and Fel d 4 specific IgE. Only one sample classified by ImmunoCAP as negative, showed slight positive signal to Fel d 4. About 98% of the patients could be diagnosed as cat allergic using the combination of these three allergens (correlation coefficient to ImmunoCAP is 0.94 with PPV=1 and NPV=0.93).

**Conclusions**

Sensitization to cat individual allergens in Russia is comparable to previous studies in other regions. Three investigated proteins together (Fel d 1, Fel d 2 and Fel d 4) are suitable for use as a sensitization markers equally as well as cat dander extract in in vitro molecular (serological) diagnostics. Moreover, component resolved approach provides an opportunity to predict course and outcome of disease, helps to prescribe adequate AIT treatment and may also be used for the monitoring of patients undergoing immunotherapy.

## A47 Topical nasal bacterial therapy associated with pre-seasonal allergoid SCIT in patients with grass allergy

### Giulio Brivio, Maria Assunta Boscolo

#### Ospedale Civile San Leopoldo Mandic Allergology Merate, Lecco, Italy

##### **Correspondence:** Giulio Brivio

**Background**

Pre-seasonal allergoid vaccine, associated with preventive bacterial nasal therapy, may be more effective in treating allergic symptoms.

**Objective**

The purpose of the study was to evaluate the effectiveness and possible side effects of preventive topical nasal therapy with a suspension of inactivated bacteria followed by a pre-seasonal allergoid subcutaneous immunotherapy (SCIT) in patients with grass allergy, compared with a group of patients treated only with an allergoid SCIT.

**Methods**

The two groups, consisting of 15 patients each, are consistent in age and sex and both monosensitised to grasses. To each patient was assigned a score card of symptoms and a possible anti-allergic drug therapy. Patients in group A were treated with a bacterial topical nasal therapy (from Anallergo SpA, Florence, Italy) during months of November and December followed by a pre-seasonal allergoid SCIT (Anallergo) during the months of February and March. The group B was treated only with the allergoid SCIT (Anallergo) during the months of February and March.

**Results**

Conjunctivitis Gr. A 993 vs Gr. B 1211, Nasal Blockage Gr.A 1315 vs Gr. B, Sneezing Gr. A 1436 vs Gr. B 1724, Rhinorrhea Gr. A 1163 vs Gr. B 1419, Cough Gr. A 798 vs Gr. B 843, Dyspnea Gr. A 611 vs Gr. B 717, Drugs Gr. A 576 vs Gr. B 759.

**Conclusions**

The results show that the total symptoms were significantly lower during and after the SCIT in patients who had taken advantage of the preventive therapy with the bacterial suspension (group A), compared to those treated only with the pre-seasonal allergoid SCIT (group B). Likewise, the use of symptomatic drugs in group A was lower than those in group B, confirming the efficacy of the protocol under study.

## A48 Simplified method for Eucapnic Voluntary Hyperventilation (EVH) challenge to diagnose Exercise Induced Bronchospasm (EIB)

### Richard Rosenthal^1, 2^, Harvey Howe^3^, Paul Knause^4^

#### ^1^Johns Hopkins School of Medicine Medicine, Baltimore, Maryland, United States; ^2^Inova Fairfax Hospital, Fairfax, VA, United States; ^3^Self, Vienna, VA, United States; ^4^Self, Arlington, VA, United States

##### **Correspondence:** Richard Rosenthal

**Background**

A vastly simplified method for the conduct of EVH challenges is presented.

**Objective**

The long established “gold standard” method for EVH challenge (Phillips Y.Y.et al, 1985) need have been simplified using more readily acquired and assembled equipment. This lends itself to greater ease of use, precision, reproducibility and interpretive value.

**Methods**

A compressed gas mixture of 5% CO2, 21% O2 and 74% N2 is required to maintain eucapnea when patients voluntarily (without exercise) hyperventilate in order to simulate the hyperpnea of exercise. The resultant dehydration of airway surface liquid is considered to osmotically cause the release of mediators to which bronchial smooth muscle responds in patients who have EIB. (Anderson, S.D., 2000). Originally, in order to reduce the gas tank pressure to ambient and regulate the volume of gas delivered, the gas was manually valved into an unwieldy weather balloon which patients had to keep at constant inflation. There was no data collection, written record or report. The simplified method, described here, employs a patient demand valve which reduces the tank pressure to ambient and a pressure gage fitted in parallel to the gas tank. The volume of gas delivered to the patient is computed from the decrease in the tank pressure after 6 minutes of voluntary hyperventilation. Using a computer loaded with the correct algorithms, patients are coached to ventilate a target volume or the volume delivered determined afterwards by reading the pressure drop from the gage. A program computes the percent of estimated Maximum Voluntary Ventilation and a record and interpretation is produced after serial pulmonary functions are compared to pre-challenge values.

**Results**

This simplified method has been successfully used to screen patients for the presence of EIB. Numerous applications include determination of suitability for military duty, respiratory health certification for SCUBA diving, evaluation of dyspnea of unknown origin, workup of asthma for more individualized treatment, evaluation of disability, evaluation of cold weather athletes, therapeutic use exemptions for otherwise prohibited drugs in IOC and other sanctioned athletic events and pharmaceutical studies of drugs designed to treat the EIB component of asthma.

**Conclusions**

The universally accepted, well documented EVH challenge for EIB was unwieldy, not generally available and therefore too rarely used. The easily conducted simplified method described here makes the established technique more available and will expand the applications so that patients with dyspnea will have their illnesses better characterized and therefore better treated.

## A49 An EDA-ID patient with autoimmune C3 Nephritic Factor managed with Rituximab

### Rony Greemberg, Jean Jacques De Bruycker, Isabel Fernandez, Françoise Le Deist, Elie Haddad

#### CHU Sainte-Justine, Allergy & Clinical Immunology, Montreal, Canada

##### **Correspondence:** Rony Greemberg

**Background**

Hypomorphic IKBKG/NEMO mutations in males lead to hypohidrotic ectodermal dysplasia with immunodeficiency (EDA-ID) as well as immunodeficiency without EDA. IKBKG mutations may also present with autoimmune manifestations, including inflammatory bowel disease, arthritis and autoimmune hemolytic anemia.

We report a NEMO patient with autoimmune underlying process. Specifically, the presence of Nephritic Factor, C3Nef, an autoantibody that binds to the alternative complement pathway C3 convertase, C3bBb, preventing its decay, and leading to the consumption of C3 complement.

**Case Report**

A 5 years old male presented with multiple pyogenic bacterial infections, recurrent febrile episodes, sparse hair, hypodontia and conical shaped teeth. Genetic Testing identified a deletion of 6 nucleotides in exon 4, 487_492delGCCACT, in the IKBKG gene, resulting in the frame deletion of 2 amino acids. Subsequently, the patient was diagnosed with EDA-ID.

While on Immunoglobulin replacement therapy, he developed sepsis with Streptococcus Pneumoniae bacteremia. The patient also had a chronic EBV infection. Further investigative workup was significant for severe decrease of both CH50 and complement C3, and for the presence C3 Nephritic Factor- C3Nef. Interestingly, the patient had no hematuria, no proteinuria and renal function was normal.

Rituximab, was chosen as a therapy option, with the purpose to eliminate both the B cell reservoir of EBV and the production source for the autoantibody C3Nef.

Rituximab treatment induced a transient and moderate increase of CH50 levels and reduced EBV viremia.

**Conclusions**

To our knowledge, this is the first NEMO patient described with autoantibody C3Nef.

We encourage to investigate NEMO patients with low complement C3 and CH50 for the presence of the possibly overlooked autoantibody C3Nef. One could hypothesize that this low complement level could be the reason for increased susceptibility to multiple pyogenic infections in this patient. Our case also describes Rituximab as a safe therapeutic option although the level of CH50 were not normalized.

**Consent**

The authors received informed consent from the patient’s guardian/parent to publish.

## A50 Sociodemographic and socioeconomic risk factors for adolescent atopic dermatitis in South Korea

### Yeong Ho Rha^1^, Kyung Suk Lee^2^, Sun Hee Choi^1^

#### ^1^Kyung Hee University School of Medicine Pediatrics, Seoul, South Korea; ^2^CHA Unitersity Scool of Medicine Pediatircs, Bungdang, South Korea

##### **Correspondence:** Yeong Ho Rha

**Background**

There have been many suggestions for medical, socioeconomic and sociodemographic risk factors for atopic dermatitis (AD) from the studies in an attempt to prevent and treat AD.

**Objective**

We conducted this study specifically focusing on the relationship between various socioeconomic and demographic variables, and AD in Korean adolescents.

**Methods**

We used data of Korea Youth Risk Behavior Web-Based Survey (KYRBWS) which includes 79,202 Korean adolescents aged 12 to 18 years. Data was collected by the self-answering surveying method in 2010. Dependent variable was atopic dermatitis; independent variables were gender, parents’ education level, family affluence scale (FAS), low subjective family economic status, and obesity. Multivariate analysis was conducted in order to analyze the relationship between socioeconomic and sociodemographic risk factors and AD.

**Results**

The prevalence of AD based on KYRBWS 2010 in Korean adolescents was 23.1%. In univariate analysis, female, urban, high parental education level, FAS was strongly correlated with AD. In multivariate analysis, female, urban, college or higher parental education level, obesity, high FAS, low subjective family economic status were correlated with AD.

**Conclusions**

We found that Korean adolescents’ AD was strongly correlated with socioeconomic and sociodemographic risk factors. Thus, it is important to modulate the socioeconomic and sociodemographic risk factors to control AD systematically in adolescent in Korea.

## A51 Prescription patterns of epinephrine auto-injectors for treatment of anaphylaxis in Manitoba children

### Herman Tam, Estelle Simons, Elinor Simons

#### Department of Pediatrics and Child Health, University of Manitoba, Winnipeg, Canada

##### **Correspondence:** Herman Tam

**Background**

Epinephrine is the first-line medication for treatment of anaphylaxis. Recent data pertaining to dispensing patterns of epinephrine auto-injectors (EAIs) are limited.

**Methods**

Using the Drug Programs Information Network (DPIN) Manitoba administrative pharmaceutical claims database, we analyzed dispensing data for EAIs among 0- to 16-year-olds from January 1, 2011 to March 31, 2015. We identified the number and percentage of children and teenagers for whom at least one EAI was dispensed. We evaluated the appropriateness of the EAI dose (0.15 mg or 0.3 mg) for the child’s age. We also conducted adjusted multivariable logistic regression to determine if concomitant prescription of an asthma medication, family income quintile, and rural versus urban region of residence affected the odds of dispensing a higher-than-expected or lower-than-expected dose.

**Results**

EAIs were dispensed 25,562 times for 8,998 children (2.5% of the pediatric population). EpiPen and Allerject were the two most common EAIs dispensed (98.2%); 53.6% of doses were 0.15 mg and 46.4% were 0.3 mg. Three percent of prescriptions may have been inappropriately dosed based on age (1.62% of children at or below age 5 years were dispensed a 0.3 mg EAI, 95% CI: 1.36%-1.94% and 1.44% of children at or above age 12 years were dispensed a 0.15 mg EAI, 95% CI: 1.13%-1.83%). Children at or below age 5 years with a higher-than-expected dose had higher odds of concomitant asthma medication prescription (odds ratio [OR] 1.36, 95% CI: 1.08-1.72), family income in the lowest 20% (OR 2.90, 95% CI: 2.02-4.14), and rural residence (OR 1.43, 95% CI: 1.12-1.82). Children at or above age 12 years with a lower-than-expected dose had higher odds of having family income in the lowest 20% (OR 2.36, 95% CI: 1.33-4.20) but not of concomitant asthma medication prescription (OR 0.72, 95% CI: 0.49-1.05) or rural residence (OR 1.39, 95% CI: 0.94-2.04).

**Conclusions**

Most EAIs were dosed appropriately based on the age of the child. Anaphylaxis comorbidity and barriers to accessing emergency anaphylaxis treatment were associated with unexpectedly high EAI doses prescribed for younger children. Older children prescribed the lower dose may have had poor access to healthcare for prescription updates. Factors accounting for dosing preferences may be important for improving the proportion of children for whom the correct dose of EAI prescription is dispensed.

## A52 The complexity of immune diagnosis child’s with recurrent inflammation of the respiratory tract, suspected food allergy and impaired immunity – case report

### Maria Golebiowska-Wawrzyniak, Katarzyna Markiewicz

#### Institute of Mother and Child, Clinical Immunology, Warszawa, Poland

##### **Correspondence:** Maria Golebiowska-Wawrzyniak

**Background**

5-year-old girl referred to the Clinic Immune Department due to recurrent pharyngitis, bronchitis and pneumonia running with plenty of mucous secretions, was examined. Symptoms above were accompanied by recurrent abdominal pain and abnormal abundant stools - constipation or diarrhea. Written informed consent in accordance with the Declaration of Helsinki was obtained from the parents of the child before the examination, and the parents and the child were informed about the objective of the study, as well as the course of the examination and tests.

**Objective**

The purpose of the study is to diagnose the reasons of recurrent inflammation of the respiratory tract.

**Methods**

For evaluation of child’s immune status the immunological profile was prepared : concentration of IgG, IgA, IgM made by nephelometry; lymphocyte blastic transformation test (LBTT) of peripheral blood with phytohemagglutinin, milk antigens, flour antigens and autotransformation as a control – morphological method; spontaneous reduction of Nitrotetrazolium Blue Test (NBT) by neutrophils, and phagocytosis assay. For allergy evaluation the allergology profile was made: total IgE concentration; IgE specific for inhaled and food allergens (Euroimmun panels and Phadia ImmunoCAP – ELFA method) also manual LBTT test with milk and flour antigens and testing for celiac disease were made.

**Results**

IgG, IgA, IgM concentrations were not elevated. Impairment of T-cell function. Food allergy IgE-independent - increased LBTT with milk and flour antigens. Stimulation index for flour in the normal range, but the percentage value is increased (35%). Autotransformation (LBTT) increased (32%; norm -15%) which affects the value of the index, suggesting the correct result. Allergy IgE-dependent, the presence of sIgE for d1 and t3 (0.38 KU/L; 0.55 KU/L; ImmunoCAP). Confirmed intolerance to gluten (tTG - IgA 394.4 ELIA U/ml; tTG - IgG 33.0 ELIA U/ml, norm <7 ELIA U/ml).

**Conclusions**

Recurrent inflammation of the respiratory tract in children require performance of the enhanced diagnostics covering testing of the immunological and allergological profile. Frequently, gastrological diagnostics is added.

## A53 The role of skin prick test and oral challenge in the diagnosis of amoxicillin and amoxicillin/clavulanate allergy in children

### Yoram Faitelson^1^, Miguel Stein^1^, Avigdor Mandelberg^2^, Ilan Dalal^1^

#### ^1^Sackler Faculty of Medicine, Tel Aviv University, Tel Aviv, Israel Allergy and Immunology Unit, Wolfson Medical Center, Holon, Israel; ^2^Sackler Faculty of Medicine, Tel Aviv University, Tel Aviv, Israel The Pulmonary Unit, Department of Pediatrics, Wolfson Medical Center, Holon, Israel

##### **Correspondence:** Yoram Faitelson

**Background**

Suspected adverse reactions to amoxicillin in children are common but only rarely these reactions are truly allergic. There is no standardized test that establishes the diagnosis of amoxicillin or amoxicillin/clavulanate allergy in children and current methods that are used such as skin prick test (SPT) are painful and time consuming.

**Objective**

We assessed the utility of SPT followed by oral graded challenge in the diagnosis of amoxicillin and amoxicillin/clavulanate allergies in children.

**Methods**

Children with a history of immediate or non-immediate reactions to amoxicillin or amoxicillin/clavulanate, were tested by SPT with amoxicillin and penicillin G, followed by oral graded challenge with amoxicillin or amoxicillin/clavulanate, respectively. Those who had negative challenge were instructed to continue the oral challenge for 2 consecutive days at home. Data were collected on clinical characteristics of the adverse reaction, personal and relatives’ drug allergies, and history of personal and relatives’ atopy.

**Results**

One hundred and thirty three children were enrolled to this study. Median age at time of study - 4.1 years (range, 0.7-18 years); 70 (52%) were male. One hundred and twenty six children (95%) had suspected allergic reaction (mainly rash) to amoxicillin and 7 (5%) had suspected reaction to amoxicillin/clavulanate. One hundred and twenty eight children (96%) were tested by SPT with amoxicillin and penicillin G and none had a positive reaction. All the children underwent an oral graded challenge with amoxicillin or amoxicillin/clavulanate. There were 3 children (2%) who had mild immediate reaction and 7 children (5%) who had mild non-immediate reaction to amoxicillin. Statistical analysis revealed that family history of drug allergy (adjusted odds ratio = 7.9; 95% CI, 1.9-32.6) was associated with failure of the oral challenge. History of a reaction occurring after 3 days of exposure was associated with successful challenge (adjusted odds ratio = 6.8; 95% CI, 0.8-56.3). Age, gender and a history of atopy were not statistically significant different between those who passed and those who failed the oral challenge.

**Conclusion**

In our cohort, the true incidence of allergy was only 7%. Oral graded challenge was safe and SPT with amoxicillin and penicillin G did not contribute to the diagnosis of allergy. History of a reaction occurring after 3 days of exposure predicted successful challenge while family history of drug allergy was associated with challenge’s failure. Further studies with larger population are needed to confirm the usefulness of SPT in the diagnosis of allergic reactions to amoxicillin or amoxicillin/clavulanate in children.

## A54 Socio-economic status influences the prevalence of food sensitisation and food allergy in urban Cape Town children

### Michael Levin^1^, Lelani Hobane^2^, Wisdom Basera^2^, Maresa Botha^1^, Claudia Gray^1^, Heather Zar^3^

#### ^1^University of Cape Town, Paediatric Allergology, Cape Town, South Africa; ^2^University of Cape Town, Public health, Cape Town, South Africa; ^3^University of Cape Town, Paediatrics, Cape Town, South Africa

##### **Correspondence:** Michael Levin

**Background**

Health outcomes are known to be influenced by socio-economic status (SES), however, there is limited data exploring the relationship between SES and food sensitisation (FS) or food allergy (FA) in children.

**Objective**

To describe prevalence and explore associations of FS and FA to SES (measured using household size, parental education, household income and employment status) in urban Cape Town children.

**Methods**

Prevalence of FS and FA was assessed in the South African Food sensitisation and Food Allergy study in 739 of 764 eligible 1-3 year old children (96.7% participation rate) attending randomly selected crèches in the Cape Town metropole. Skin prick testing (SPT) was done to 7 commonly allergenic foods (egg, milk, peanut, wheat, soy, fish and hazelnut) and food allergy confirmed by open food challenge. Associations between SES and FS/FA were assessed using the Z-test, Chi-square/Fisher’s exact and Wilcoxon Ranksum tests.

**Results**

In 739 participants, 91 were sensitised at any degree of reactivity to 1 or more foods and 648 negative for all foods (87.7%). FS prevalence at SPT≥1mm to any food was 12.3%, at SPT≥3mm 9.6% and at SPT≥7mm 4.5%. Challenge proven IgE-mediated FA was 2.4%.

Statistically significantly higher prevalence of FS was seen in children with employed (or student) parents rather than unemployed parents (none unemployed 13.5%, one unemployed 8.2%, both unemployed 0%; *p*=0.03). Parents of children with sensitisation had significantly higher total monthly household income. The disparity in household income in those with and without sensitisation increased with increasing cut-off levels of sensitisation from R2000/month at SPT≥1mm to any food (*p*=0.06), to R3500/month at SPT≥3mm (*p*=0.02) and R6000/month at SPT≥7mm (*p*=0.02).

A higher prevalence of sensitisation was apparent in children with parents who attained tertiary education compared to parents who attained primary/secondary education however these results did not achieve statistical significance.

Total cumulative skin tests did not show any significant associations with any SES measure. Differences in FA patterns were evident but low numbers preclude meaningful assessment of significance. Household size showed no association with FS and FA. No significant differences in sensitisation patterns were noted between ethnic groups.

**Conclusions**

Certain markers of SES are associated with food sensitisation in young children in Cape Town. Enlargement of the cohort may allow the effect of SES on food allergy to be assessed.

## A55 Different therapeutic response in smoking and non-smoking asthmatics to inhaled corticosteroids

### Biserka Jovkovska Kjaeva^1^, Zoran Arsovski^2^

#### ^1^Clinic of Pulmonology and Allergy, Skopje, Macedonia; ^2^Clinic of Pulmonology and Allergy Functional Diagnostic, Skopje, Macedonia

##### **Correspondence:** Biserka Jovkovska Kjaeva

**Background**

Evidence suggest that smoking asthmatics have impared responce to asthma therapy compared to nonsmoking asthmatics.

**Objective**

The aim of the study was to determine if there is a difference in therapeutic response to the therapeutic dose of inhaled Fluticasone propionate in patients smokers and nonsmokers with mild asthma.

**Methods**

Thirty eight patients with mild asthma, between 18-50 years old, were included in a randomized parallel study. They were divided into two groups: smokers (16) and nonsmokers (22). They have received inhaled Fluticasone propionate 250 μg bid as a regular dose for 6 weeks. As a rescue medication a short acting ß2 agonist Salbutamol a 0,1 mg was prescribed on as needed base. Lung function was assessed with Power-Cube spirometer and asthma control was assessed with asthma control test (ACT-TM).

**Results**

There was no statistic difference in both groups according to age, asthma duration and FEV1 values before treatment. There was a statistic difference between the two groups concerning ACT before treatment (*p*<0,5). After 6 weeks of treatment we found a positive effect (*p*<0,01) concerning FEV1 in nonsmoker-asthmatics compared to smoker-asthmatics. Positive effect (*p*<0,5) was found in ACT values compared between two groups.

**Conclusions**

Although we have analyzed very small group of patients in a relatively short period of time, we can conclude that median dose of inhaled corticosteroids results in good therapeutic response in nonsmoker-asthmatics. On the other hand, these doses are insufficient to achieve the same result in smoker-asthmatics and maybe the higher dose or additional therapy is needed.

## A56 Factors for therapy adherence in Chronic Urticaria

### Vesna Grivcheva-Panovska

#### School of Medicine, University St. Cyril and Methodius University, Clinic of Dermatology, Skopje, Macedonia

**Background**

Adherence to self administered medications is a major challenge both for patients with Chronic Urticaria, as well as treating physicians.

**Objective**

To determine the adherence to self administered medications and to analyze underlying associated factors using Medication Adherence Scale (MARS), Brief Adherence Rating Scale (BARS), Urticaria Activity Score and Visual Analogue Scale (VAS) regarding Chronic Urticaria treatment.

**Methods**

Questionnaires were performed by 296 Urticaria patients treated from January 2015 to June 2016 at the University Hospital of Dermatology, Unit of Allergy and Clinical Immunology as outpatients (163 female, 133 male, age 42+-5), 151 of them were Chronic Urticaria patients (98 female, 53 male, age 39+-3.5).

Education for patients with Chronic Urticaria encouraging them to take active approach as well as regular checkups are necessary in order to achieve a potentially good therapeutic outcome by increasing the adherence.

The v2 -test for categorical variables or parametric analysis for continuous variables was used to evaluate the differences in the study variables among the adherence groups. Internal consistency was assessed using Cronbach’s alpha. An acceptable Cronbach’s alpha value is considered to be 0.7 or more. Known group validity was assessed through the association of items and MARS and BARS categories using correlation coefficient and covariance. The significance level was set at P < 0.05. Mean adherence scores by MARS and BARS were 4.5 for self-administered medication.

**Results**

The reliability scores (i.e. Cronbach’s alpha) were 0.783 for self administered drugs, 75 of all Urticaria patients (25.3%) admitted that they ignored doctors’ instructions, whereas 84 of Chronic Urticaria patients (55.6%) did so. 18.5% (28/151) of Chronic Urticaria patients with disease duration longer than 1 year discontinued medications because they had self assessed themselves as cured, versus 32.45.0% (49/151) with disease duration longer than 6 months but less than one year.

Patients’ gender, educational level, work position, marital status, disease activity, type of dietary regimen, patient’s anticipation regarding outcome, as well as patient’s estimate of treating physicians’ competence, availability and dedication did not have impact on the level of adherence to therapy.

Adherence to self-administered medications was influenced by the age of the patient and duration of the symptom free phase of the disease.

Adherence to self administered medications was significantly associated with the frequency of hospital visits.

**Conclusions**

Education for patients with Chronic Urticaria encouraging them to take active approach as well as regular checkups are necessary in order to achieve a good therapeutic outcome by increasing the adherence.

## A57 Molecular characterization, gene expression profile and histopathology of fungal spore causing allergies in Southwestern Nigeria

### Adeyinka Odebode^1^, Adedotun Adekunle^1^, Peter Adeonipekun^1^, Ebenezer Farombi^2^

#### ^1^University of Lagos, Botany, Akoka, Nigeria; ^2^University of Ibadan, Biochemistry, Ibadan, Nigeria

##### **Correspondence:** Adeyinka Odebode

**Background**

The application of molecular technique could solve the challenges associated with the treatment of fungi allergy therefore the study aimed at assessing the molecular diversity of fungal spores using culture dependent approach.

**Objective**

A comparative survey of airborne fungal spores in ten different environments in Lagos and Oyo States, Nigeria was carried out for a period of Eighteen months between Jan 2015 – June 2016.

**Methods**

Using sedimentation plates (Dichloran glycerol 18 and Potato Dextrose Agar culture plate). A total of 44 spore types were identified. Genotypic identifications were accomplished through sequencing of amplified ITS1 and 4 of rDNA gene.

**Results**

The fungal strains identified belong to Ascomycetes, Deuteromycetes and Basidiomycetes. The results revealed lowest count during summer and maximum during the rainy season. Aspergillus was quite abundant in all the environments surveyed. The predominance of Aspergillus, Curvularia, Alternaria, Cladosporium, Fusarium and Penicillium in all the surveyed environments has been attributed to their ability to grow in various substrata. The mean relative gene expression values ranged from 18.95 – 31.28 for Actin, 17.38 – 26.77 for β tubulin and 19.74 – 30.63 for P. oxalicum and 30.22 – 37.56 for P. citrinum. All genes were significantly correlated to the Bestkeeper index (*p*< 0.001). Histopathology showed that Aspergillus flavus on mice lung had mild thickening of the alveolar interstitium. Alveoli are clear and devoid of exudates while there was is however moderate haemorrhages and multiple foci of alveolar macrophages laden with dark pigment materials for Penicillium citrinum. There is accumulation of inflammatory cells around blood vessels suggestive of vasculitis for Cladosporium spp while Aspergillus tamari showed widespread haemorrhage and over-distension of the alveoli indicative of pulmonary emphysema. For Penicillium chrysogenum multiple foci of necrosis and accumulation of necrotic debris was observed. A correlation has been made between the volumetric composition of airspora and the incidence of seasonal fungi allergy.

**Conclusions**

Data on the abundance/prevalence of fungi species in the atmosphere of sub-Saharan Africa is limited which necessitated this study for forecasting the prevalence of allergenic fungi in the environment at various seasons.

## A58 Temporary henna tattoos: fashion or nightmare?

### Nadezhda Camacho-Ordoñez^1^, Alejandrina Josefina Martinez- Vázquez^2^, María de la Luz H García-Cruz^3^

#### ^1^Londres Clinic Allergy and Clinical Immunology, Mexico City, Mexico; ^2^ Dalinde Medical Center Allergy and Clinical Immunology, Mexico City, Mexico; ^3^National Institute of Respiratory Diseases Allergy and Immunogenetic Research Department, Mexico City, Mexico

##### **Correspondence:** María de la Luz H García-Cruz

**Background**

Nowadays temporary paint-on tattoos have become popular worldwide. A common dye for such tattoos is henna (Lawsonia inermis). Para-phenylendiamine (PPD) is a coloring agent and potent sensitizer commonly added to henna tattoos to darken tattoo color. Contact allergy to temporary henna it is progressively more common reported.

**Objective**

To describe three cases of allergic contact dermatitis (ACD) to temporary tattoos with positive PPD reactions. They were followed 2 years after treatment.

**Clinical cases**

Case 1: a 33-year-old male presented with a 6-month history of an erythematous, pruritic, papulovesicular rash on his right upper arm after daily contact with a henna tattoo of a friend. He had history of a similar reaction 2 years ago; 3 days after application of a temporary black henna tattoo while he was on holiday. Case 2: a 32- year-old female had two black henna tattoos on arms while holiday in Cancun Mexico in August 2013. 2 hours later tattoos became hot, red raised and itchy. Case 3: a 22-year-old girl with previous history of erythematous and pruritic plaques on the back 5 hours after application of a temporary tattoo one month ago. She presented with a severe ACD on the scalp an hour after application of hair dye. All three patients were patch tested and they were positive to PPD. They were treated with antihistamines, topical corticosteroids and emollients. An improvement was noted after 48h hours. After 2-year follow-up, we documented hyperpigmentation in 2 patients and hipopigmentary changes in one. We could not say if this is a prolonged postinflamatory reaction, or in the case of hypopigmentation, the henna dye is acting as a sunblock agent.

**Conclusions**

In most cases allergic reactions are caused by the mixtures, which contain not only natural henna but also many chemical coloring agents such as PPD. Patients who developed an allergic reaction to PPD should be advised by physicians to avoid any further exposure to PPD, which is contained in cosmetic products such as hair-dyes or semi permanent eyebrow make-up; as has been described with our third patient. Besides, skin tattoos with black henna should be avoided, especially during foreign travel, as this can make a health management challenge.

**Consent**

The authors received informed consent from the patients to publish.

## A59 Immunological and clinical effects of rhIL-2 therapy in patients with MDR-TB

### Qi Tan, Rui Min, Guan-qun Dai, Wei-Ping Xie, Huang Mao, Hong Wang

#### The First Affiliated Hospital of Nanjing Medical University, Department of Respiratory and Critical Care Medicine, Nanjing, China

##### **Correspondence:** Qi Tan

**Background**

Multidrug-resistant tuberculosis(MDR-TB) has emerged as a lethal global threat and the treatment of MDR-TB is a challenge worldwide. Thus, it is urgent to find an optimised therapy regimen for the control of MDR-TB globally.

**Objective**

We launched the study to investigate the clinical outcome and immunomodulation mechanisms induced by recombinant human interleukin-2 (rhIL-2) treatment in MDR-TB patients.

**Methods**

From 2009.1 to 2013.3, MDR-TB cases were enroled and followed up in this prospective, randomised, controlled multicentre study (2008zx100 03-014), study group were administered rhIL-2 with the standard chemotherapy of 18-month duration while control group under the standard chemotherapy of 24-months duration. Clinical and safety outcomes and immune function observation were compared between the two groups.

**Results**

Of 124 enroled MDR-TB patients, 64 in control group, 16 cured (25%), with 1 died(3%), 9 default(15%), while 60 in rhIL-2 group, 32 cured (53%), with 3 died(5%), 10 default(16%). Adverse events were generally those commonly associated with MDR-TB treatment. Rates for any of the adverse events(AE) in two groups were similar(*p*>0.05). Patients from study group under rhIL-2 treatment showed increasing Th1 expression and decreasing Th17 and Treg expressions while the relative levels of IL-17A mRNA, ROR-γt mRNA, and Foxp3 mRNA decreased and their level of IFN-γ mRNA increased in PBMCs, compared with control group.

**Conclusions**

The study revealed that adjunctive treatment with rIL-2 induced an outcome improvement with safty in MDR-TB patients and was associated with recovery of Th1/Th17 balance.

## A60 Bronchospasmoltyic evaluation of synthesized 8-pyraozle substituted xanthine derivatives

### Rakesh Yadav, Sneha Singh, Divya Yadav

#### Banasthali University, Pharmaceutical Chemistry, Banasthali, India

##### **Correspondence:** Rakesh Yadav

**Background**

Asthma and chronic obstructive pulmonary disease (COPD) are categorized as chronic disease all over the globe and its pervasiveness is increasing especially in the pediatric population. It is easily diagnosed by the accumulation of mucus in the lumen of the airways and is not a disease with a single etiology but a very complex syndrome. Several positions of xanthine skeleton with different substituents have been explored to obtain a variety of potent derivatives.

**Objective**

Present investigation has been made to evaluate the synthesized 8-pyraozle substituted xanthine derivatives for bronchospasmoltyic activity (in vivo)

**Methods**

Synthesis-Number of new 8-pyrazole substituted xanthine derivatives has been synthesized as per the reported literature.Substituted pyrazoles (1-6) were prepared by thermal fusion with appropriate amines like morpholine, pyrrolidine, piperidine, piperazine, 1-methylpiperazine, dimethylamine and diethylamine. The completion of the reaction was monitored by thin layer chromatography (TLC). The residue so obtained was washed with diethyl ether and the compound obtained was used as such for further reaction. These pyrazole derivatives (1-6) were further reacted with 5,6-diamino-1,3-dimethyluracil in the presence of 1-ethyl-3-(3-dimethylaminopropyl)carbodiimide (EDCI) and methanol at room temperature to yield the carboxamide derivatives (7-12)which on subsequent ring closure yielded the titled compounds (13-18).

Biological activity- The newly synthesized xanthine derivatives were evaluated for in vivo bronchospasmolytic activity against histamine aerosol induced bronchospasm in guinea pig as per Yadav et al. The morpholine and dimethylamine substituted derivatives showed pronounced bronchospasmolytic effect in comparison to the standard drug theophylline in guinea pig animal model.

**Results & Conclusions**

The chemical sturucture of synthesized 8-pyrazole substituted xanthines were characterized using various spectral techniques viz. FT-IR, NMR etc. The effects of varying substituents and their location on the 8-pyrazole ring are clearly visible in the pharmacological characteristics of these novel xanthine derivatives. Suitable introduction of 8-pyrazole substituents on the xanthine scaffold results an important trait for potent bronchospasmolytic effects.

References:

1. Rakesh Yadav, Ranju Bansal, Suman Rohilla, Sonja Kachler, K. N. Klotz. Synthesis and pharmacological characterization of novel xanthine carboxylate amides as A2A adenosine receptor ligands exhibiting bronchospasmolytic activity. Bioorganic Chemistry, 2016, 65, 26-37.

2. Rakesh Yadav, Ranju Bansal, Sonja Kachler, K. N. Klotz. Novel 8-(p-substituted-phenyl/benzyl)xanthines with selectivity for the A2A adenosine receptor possess bronchospasmolytic activity. European Journal of Medicinal Chemistry 03/2014; 75:327–335. DOI:10.1016/j.ejmech.2014.01.045

3. Rakesh Yadav, Divya Yadav, Ranju Bansal and Anurag Kuhad. Synthesis and pharmacological evaluation of 8-substituted phenyl xanthines for asthma therapy. Frontiers in Immunology 01/2013; 4. DOI:10.3389/conf.fimmu.2013.02.00645

## A61 Peri-anaesthetic anaphylaxis; comparisons between cardiovascular manifestations in children and adults

### Ekaterina Khaleva^1^, Henry T. Bahnson^2^, Amber Franz^3^, Lene Heise Garvey^4^, Nicola Jay^5^, Rubaiyat Haque^6^, Adam Fox^1^, Gideon Lack^1^, George du Toit^1^

#### ^1^Guy’s and St Thomas’ NHS Foundation Trust, Children’s Drug Allergy Clinic, London, UK; ^2^Benaroya Research Institute, Seattle, WA, USA; ^3^Seattle Children’s Hospital, Seattle, WA, USA; ^4^University Hospital Gentofte, Danish Anaesthesia Allergy Centre, Allergy Clinic, Department of Dermato-Allergology, Gentofte, Denmark; ^5^Sheffield Children, Sheffield, UK; ^6^Guy’s and St Thomas’ NHS Foundation Trust, Adult’s Drug Allergy Clinic, London, UK

##### **Correspondence:** Ekaterina Khaleva

**Background**

Anaphylaxis during anaesthesia is rare, sometimes difficult to diagnose, and may have life-threatening consequences if untreated. Relatively little is known of the cardiovascular sequence of anaphylaxis and how children and adults may be different.

**Objective**

The aim of this retrospective audit was to document time-dependent cardiovascular symptoms and signs that occur during anaphylaxis in the peri-anaesthetic environment.

**Methods**

We performed a retrospective study of all patients with suspected perioperative anaphylaxis in the GSTT during 2008-2014. The time sequence of cardiovascular symptoms including lower systolic and diastolic blood pressure (BP), heart rate (HR) and time from causative drug to treatment of anaphylaxis with epinephrine were noted.

**Results**

A total of 49 patients with confirmed peri-anaesthetic anaphylaxis were included. 16 were excluded due to missing or incomplete anaesthetic charts. Out of 49, 15 were children (6yo average age) and 34 adults (52yo average age); 22 males and 27 females. In children the lowest recorded systolic BP was 35 mm Hg (mean 53.1 mm Hg) and diastolic 15 mm Hg (mean 26.64 mm Hg), while in adults 30 mm Hg (mean 52.78 mm Hg) and 12 mm Hg (mean 30.08 mm Hg) respectively. The mean time of documented hypotention after induction of anaesthesia was 8.7 min (SD ±9.5) in children and 27.47 min (SD ±14.33) in adults and after administration of the causative drug (when known) was 4.67 min (SD ±6.43) in children and 11.45 min (SD ±17.33) in adults. Of the 49 cases of anaphylaxis, 24 (49%) were accompanied by tachycardia, the mean highest HR in children was 147.2 bpm and 125.06 bpm in adults. Recorded hypotension preceded recorded tachycardia in 3 adults and mean time of this to occur was 5 min. Arrhythmia was observed in 3 (9%) adults but not in children. One adult and one child experienced cardiac arrest and required cardiopulmonary resuscitation. Both adrenaline and metaraminol were administered in 12 cases. The overall median time from causative drug administration to adrenaline was 6 min (range, 1-30).

**Conclusions**

Our retrospective evaluation of cardiovascular manifestations during peri-anaesthetic anaphylaxis demonstrates severe hypotension (in both adults and children). Anaphylaxis and tachycardia can develop rapidly after induction, and may develop sooner in children as compared to adults.

## A62 Allergic rhinitis among university students in Belgrade

### Snezana Radic^1^, Branislava Milenkovic^2^, Ana Neskovic^1^, Ljiljana Danojevic^3^

#### ^1^Clinical Hospital Center Dr Dragisa Misovic Dedinje Children’s Hospital for Respiratory Diseases and TB, Belgrade, Serbia; ^2^Clinical Centre of Serbia,Faculty of Medicine, University of Belgrade Clinic for Pulmonary Diseases, Belgrade, Serbia; ^3^Gerontology Center Belzanijska kosa Medical practice, Belgrade, Serbia

##### **Correspondence:** Snezana Radic

**Background**

Allergic rhinitis is one of the most common chronic disease, and its prevalence has been increasing in many parts of the world in recent decades, especially in developed countries. It is a desease caused by IgE interference following contact with allergens. Allergic rhinits may be seasonal, perennial, or episodic. It is the condition which could strongly interfere with patients well being and quality of life. There were few studies about prevalence of allergic rhinitis among university students in the world.

**Objective**

To estimate the prevalence of of allergic rhinitis, respiratory symptoms and smoking habits among population of university students in Belgrade, Serbia.

**Methods**

Questionnaire based on the European Community Respiratory Health Survey (ERCHS) protocol was used to obtain data.

**Results**

We have analysed 14 questions about respiratory symptoms in 5045 university students in Belgrade, mean age 21.5±1.7 years. There were 2259 (44.8%) of male and 2786 (55.2%) of female studets. The prevalence of allergic rhinitis was 20.8% (N 1048). The majority of respondents were non-smokers (73.6%), 26.4% current and 3.3% were ex smokers. Allergic rhinitis was more prevalent in males (22.8% vs 19.1%, *p*<0.001). There were 71.4% non-smoking and 28.6% smoking studets with allergic rhinitis. There were 18.0% of subjects with allergic rhinitis who use asthma medication, which was sifnificantelly more than 5% who use asthma medications in general population of students.

**Conclusions**

There is a high prevalence of allergic rhinitis among university students in Belgrade, Serbia, which is in consistency with findings of other studies.

## A63 Safety and efficacy of first versus subsequent foods oral immunotherapy

### Liat Nachshon, Michael Goldberg, Michael Levy, Yitzhak Katz, Arnon Elizur

#### Assaf Harofeh Medical Center Allergy and Immunology, Zerifin, Israel

##### **Correspondence:** Liat Nachshon

**Background**

We previously described a treatment program of oral immunotherapy (OIT) for desensitization to milk (Levy et al. Ann Allergy Asthma Immunol. 2014 Jan;112(1):58–63), which has expanded to include peanut, egg and sesame OIT. In some patients with multiple food allergies, sequential OIT treatments for the different foods may be required.

**Objective**

We aimed to determine whether the clinical course and results of patients undergoing sequential OIT treatments differed from those who completed their first OIT treatment program.

**Methods**

Patients (*n*=21, ages 4-23 years) with multiple food allergies to a combination of milk, peanuts, egg and sesame, were enrolled into an OIT treatment program. A starting dose below the eliciting dose was determined and increased on a monthly basis while under medical supervision, until the goal of 3.0 gram food protein was achieved and consumed daily. While continuing to consume the initial desensitized food, patients were enrolled in sequential OIT treatments. Except for sesame OIT (only available since 2014), the decision for which food was to be treated first was individualized based on the patient’s respective difficulties in maintaining avoidance to a particular food.

**Results**

No significant differences were noted in achieving the final target dose between those in their sequential OIT treatments (6 each for milk and peanut, and 5 each for egg and sesame) and those during their first OIT treatment (10 to milk, 7 to egg and 4 to peanut) (77% (17/22) versus 85.7% (18/21), respectively). However, the average number of reactions during home treatment and overall treatment reactions was significantly lower during the sequential OIT programs compared to the first OIT desensitization (10 versus 27, *p*=0.04, and 17 versus 30, *p*=0.05, respectively). Furthermore, the treatment duration was significantly shorter during sequential OIT desensitizations as compared to the first OIT program (5 months versus 8.4 months (*p*<0.0001), perhaps related to the median starting dose achieved during the first induction (90mg versus 25mg, sequential versus first OIT, respectively, *p*=ns)

**Conclusions**

Although similarly efficacious, sequential OIT treatments to the subsequent food are of shorter duration and involve a lower risk for reactions than the first OIT treatment.

## A64 Hereditary Angiedema (HAE) in children and adolescents

### Cristine Rosario, Juliana Kasper, Herbeto Chong-Neto, Carlos Riedi, Nelson Rosario

#### Federal university of Parana Allergy and Immunology, Curitiba, Brazil

##### **Correspondence:** Cristine Rosario

**Background**

Hereditary Angioedema (HAE) is a difficult to treat disease, characterized by recurrent edema attacks and low serum complement levels.

**Objectives**

To report clinical and laboratory features of children and adolescents and their treatment in a specialized clinic in Brazil.

**Methods**

Retrospective analysis of 11 cases of HAE in children and adolescents by chart review and laboratory results.

**Results**

11 cases of HAE due to C1 inhibitor (C1-INH) deficiency in children and adolescents have been followed-up at the University Clinic. Symptoms first appeared between the age of 1 and 8 years (median 2.5 years-old). Eight children were male (73%). All patients had family history of angioedema. Duration of crisis varied from 0.25 to 7 days (average 2.4 days). Nine children had complained of abdominal pain, seven presented limb edema, five had facial edema and two had laryngeal edema. Serum C4 levels ranged from 5.4 to 7.3mg/dL (median 6.2mg/dL), quantitative C1-INH from 4 to 24.7mg/dL (median 8.6mg/dL) and CH50 from 8.4 to 89U/mL (median 29.5U/mL). Two patients had normal C1-INH levels, but both had family history of angioedema and one of them had low C4 levels. Four children had been on prophylactic danazol with partial control of the symptoms (less frequent attacks) and five had used tranexamic acid.

**Conclusion**

HAE manifestations may begin in childhood though less symptomatic in this age group. Patients had C1-INH deficiency and low serum levels of C4 and CH50. Early diagnosis and prompt initiation of treatment lower the burden of the disease. First-line medications are available in Brazil but hardly accessible to most patients.

## A65 Determination of multiple tree nut allergies among tree nut allergic patients

### Arnon Elizur, Michael B Levy, Ronly Har-Even, Liat Nachshon, Mor Carmel, Michael R. Goldberg

#### Assaf Harofeh Medical Center Allergy and Immunology, Beer Yaakov, Israel

##### **Correspondence:** Arnon Elizur

**Background**

Patients with a single tree-nut allergy are often instructed to avoid multiple tree-nuts for fear of cross-sensitization. However, the actual percentage of co-allergy between different tree-nuts has not been well studied. The goal of this study was to examine the frequency of allergies to other tree-nuts in single tree-nut allergic patients.

**Methods**

Patients (*n*=44, median age of 8.7 years (range, 2.3 – 22.2) referred for tree-nut oral immunotherapy (OIT), were evaluated for sensitization (by SPT) and allergy to six nuts: walnut, pecan, cashew, pistachio, hazelnut and almond. Determination of the clinical allergic status was determined by an oral food challenge (OFC) unless a significant recent reaction (within the previous 2 years) was noted.

**Results**

Walnut was the most frequent allergen with 28 allergic patients, 16 were allergic to pecan, 13 to cashew, 8 to hazelnut, 5 to pistachio. None were allergic to almond. Among those tested by OFC, anaphylaxis occurred in 8/21 (38%) of walnut (eliciting dose (ED) range, 5-4200 mg), 2/5 (40%) of hazelnut (ED range, 12-3840 mg), 7/14 (50%) of pecan (ED range 18-2500 mg), 8/13 (61.5%) of cashew (ED range, 9-4000 mg), and 1/4 (25%) of pistachio (ED range, 100-2200 mg), positive oral food challenges. Treatment with IM adrenaline was required in 33% (19/57) positive OFCs. Of the 23 patients who were challenged to all six tree-nuts, 35% (8/23) had single tree-nut allergy, while 65% (15/23) had multiple nut allergy (5 to two nuts, 6 to three nuts, 3 to four nuts, and 1 with proven allergy to five tree nuts). Among walnut-allergic patients, 60% were co-allergic to pecan, 38% to cashew, 19% to pistachio and 24% to hazelnut. All pecan-allergic patients were also allergic to walnut, while co-allergy with the other tree-nuts was lower (25-43%). Among cashew allergic patients, 69% were also allergic to walnut, 55% to pecan, 55% to pistachio and 24% to hazelnut. All pistachio allergic patients were co-allergic to cashew. Nineteen out of the 23 (83%) tree nut allergic patients were allergic to half or fewer of the six nuts tested.

**Conclusions**

While multiple tree nut allergy was common in our referred population, the majority of tree nut allergic patients tolerated at least half of the six nuts tested, suggesting the elimination of all nuts is unwarranted and should first be evaluated by OFC. Future studies will determine whether walnut and cashew OIT, will cross-desensitize for pecan and pistachio allergy, respectively.

## A66 Clinical symptoms and development of infants with cow’s milk allergy fed with hydrolyzed formula

### Maia Kherkheulidze, Nani Kavlashvili, Eka Kandelaki

#### State Medical Universitym, Pediatrics, Tbilisi, Georgia

##### **Correspondence:** Maia Kherkheulidze

**Background**

Between 5% and 15% of infants show symptoms suggesting adverse reactions to cow’s milk protein (CMP), while estimates of the prevalence of cow’s milk protein allergy (CMPA) vary from 2% to 7.5%.

**Objective**

Assessment of clinical symptoms physical development of infants with cow’s milk allergy, fed with extensively hydrolyzed formula.

**Methods**

Prospective study was conducted at outpatient department of Iashvili children’s central hospital. Cow’s milk related Symptom score (COMiss), full medical history was used for assessment and Specific IgE (RAST) for CMP was used for diagnosing CMPA. Assessment of development was based on Bayley test and physical development was evaluate based on WHO Z score growth charts. We followed until 1 year studied 34 infants with proved CMPA who was fed with exclusively hydrolyzed formula. We used SPSS 19 for statistical analyses.

**Results**

The hydrolyzed formula was well tolerated. In 14,5% (*n*=5) during the first week were seen feeding problems. The CoMiSS shows significantly decrease of clinical scores after 2 weeks starting hydrolyzed formula: total score decreases from 24.44 + 4,2 to 12.3 + 2,9. (T-= 11.46328. *p* < .00001); Crying scores (4.26+2.87) and regurgitation scores (2,56+1,41) significantly decreased by 1.9 ±0.6 (t= 8.24216. *p* < .00001). and 1.36 ± 0.5 (t= 3.08527, The *p*<. 001) respectively. The percentage of infants having normal stool consistency (soft or formed stools) significantly improved from 51.2% at inclusion to 92.0% after 14 days of feeding (*P*<0,05). The Bayley scores for development do not show any significant difference from same age population data and growth z-scores, negative at study inclusion, significantly improved after starting exclusively hydrolyzed formula. At 1-year age all infants’ anthropometric parameters was between +2 Z scores. No adverse event was related to the exclusively hydrolyzed formula.

**Conclusions**

Extensively hydrolyzed formula is well tolerated and improved clinical symptoms rapidly. The improvement of growth indices and absence of related adverse events confirmed its safety.

## A67 Prevalence and risk factors of allergic diseases

### Nino Adamai, Maia Kherkheulidze, Lia Jorjoliani, Irma Ubiria

#### Tbilisi State Medical University, Department of Pediatrics, Tbilisi, Georgia

##### **Correspondence:** Maia Kherkheulidze

**Background**

Allergic diseases have an increasing tendency.

**Objective**

Correlation between prevalence of atopic dermatitis, hives, herpes, diseases with angiodermatitis in children’s population.

**Methods**

Research was conducted in 2015, on the basis of questioning of random and representative population of children in Tbilisi and Kutaisi. Studied population included 2035 children aged from 6 to 15. Risk factors were studied through interviewing, with specially developed map-questionnaire, clinical-laboratory data; for assessment of the risk factors the case control method was applied. Statistical processing of the materials was provided by means of SPSS/v12 software. IgE study was performed as well, for the specific allergens, skin samples were taken for identification of the allergen (prink test).

**Results**

Based on the analysis of risk factors in the studied population - frequencies of presence of the dust collectors, moisture and mildew, active tobacco consumption. Frequencies of the identified variables were reliably higher (*p*<0.5) in the families of children with allergies, compared with the healthy population. Atopic dermatitis was diagnosed in 6.5% of cases, frequencies of hives (8.9%), angiodermatitis was identified in 3.2% of the adolescents, medication-caused allergy was indicated in 2.6% of children’s population, congenital herpes viral infection was indicated in 0.7% of the population. 41.3% of the studied population had positive skin prick-test. In 3.9% of the studied population IgE value was 6 times higher than norm (*p*=0.01).

**Conclusions**

Regarding the obtained results we can conclude that there is increased allergen load *p*=0.05 that could be managed based on the early and targeted prevention program, taking into consideration the regional characteristics.

## A68 Hamilton stock epinephrine pilot study: facilitators and barriers to implementation

### Andrea Burke^1^, Ernie Avilla^2^, Monika Kastner^3^, Susan Waserman^1^

#### ^1^McMaster University, Clinical Immunology & Allergy, Hamilton, Canada; ^2^McMaster University, Medicine, Hamilton, Canada; ^3^North York General Hospital, Knowledge Translation and Implementation, Toronto, Canada

##### **Correspondence:** Andrea Burke

**Background**

The scope of accidental exposure in food allergic individuals dining out is unknown. Risk for these individuals is accentuated when they forget to carry their epinephrine auto-injector (EAI) or do not have it at the time of a reaction. Our aim was to identify facilitators and barriers to implementing a stock epinephrine program at a shopping mall food court and stand-alone restaurants. This was part of the first pilot study of such a program in Canada.

**Methods**

We conducted a qualitative study to evaluate facilitators and barriers of implementing a stock epinephrine program at a shopping mall and two stand-alone restaurants. A purposive sampling strategy was used to recruit program implementation team members (security guards, food service workers, mall administration, representatives of Food Allergy Canada, and the City of Hamilton) to participate in one-on-one qualitative interviews. Analysis involved content analysis of qualitative data.

**Results**

A total of eight participants identified four key factors that contributed to organizational compatibility of providing stock epinephrine. These included (1) a shared passion for food allergy and anaphylaxis; (2) ease of program implementation (clear emergency protocols); (3) the fact that the program was neither resource nor time intensive and (4) low overhead costs. The majority of respondents were “very optimistic” about the program rolling out in other settings and identified program benefits such as providing access to emergency medication to those in need. Other benefits included the potential to reduce healthcare costs through early administration of epinephrine and improved health outcomes, and providing a template for others who may want to implement a stock epinephrine program. One of the key barriers to widespread acceptance of this pilot study, and for any future initiatives, was fear and lack of understanding of liability and how existing legislation applied to malls and food service establishments.

**Conclusions**

To our knowledge there are no studies that describe a stock epinephrine program in public settings. In spite of its positive attributes, food service establishments remain reluctant to participate because of fears related to liability. It is important to reinforce, that a stock epinephrine program is meant to complement and not replace a food allergic individual’s responsibility to self-manage and carry an EAI.

## A69 Dermatological toxicities after targeted anti-tumor therapy

### Denica Zheleva, Razvigor Darlenski

#### Tokuda Hospital Sofia, Dermatology and venereology, Sofia, Bulgaria

##### **Correspondence:** Denica Zheleva

**Background**

In the past decade a plethora of targeted anti-tumor therapies has been introduced. Herein we report two cases of dermatological adverse efects after treatment with epidermal growth factor inhibitors (EGFRIs).

**Clinical case 1:**

A 52-year-old female Caucasian with colorectal adenocarcinoma (pT3N2M1), developed severe skin rash 2 months after the start of therapy with the tyrosine kinase inhibitor- Erolitinib. The dermatological examination revealed generalized papulopustular reaction, accompanied by severe pruritus, xerosis, trichomegaly of the eyelashes and hypertrichosis of the face, fissures and hyperkeratosis of the palmes.

Therapy included topical emolients and high-potencycorticosteroid cream- Betamethasone dipropionate 0,05% with slight improvement in the dermatological status.

Clinical case 2:

A 32-year-old female from India was diagonsed with lung cancer and multi-organ metastases. She has started target therapy with the human IgG2 monoclonal antibody- Pantimumab. One month later she developed acneiform eruption of the face. The therapy has started with Doxycycilne 100mg BID and topical gel nadifloxacin 1%.2 weeks later a new flare up was observed and clindamycin 1% soutiion and topical cream with special Fluidactiv® patent were prescribed. Poor therapeutic response was observed.

**Results**

In both cases, the mild character of the adverse skin reactions requested no withdraw of EGFRI treatment. However, both patients decided to discontinue therapy.

**Conclusions**

The most common adverse reactions associated with EGFRIs, occurring in more than 50% of patients who receive treatment include dry skin, pruritus, papulo-pustular eruption, paronychia and fissuring, trichomegaly and curling of eyelashes and eyebrows and hypertrichosis of the face. There is a positive correlation between the occurrence and severity of cutaneous adverse effects and tumor response. Our cases corroborate these observations. To avoid therapy discontinuation in such cases it is worth to provide reactive or proactive management strategies.

**Consent**

The authors received informed consent from the patients to publish.

## A70 Anti-IgE therapy in Chronic Spontaneous Urticaria

### Konstantinos Bozinakis^1^, Anastasios Kriebardis^2^, Sofia Styliara^1^, Aikaterini Karastathi^1^, Nikolaos Farmakas^1^

#### ^1^General Hospital of Nikea-piraeus, Allergy and immunology, Nikea-Piraeus, Greece; ^2^Tei Tei, Aigaleo, Greece

##### **Correspondence:** Nikolaos Farmakas

**Background**

Urticaria is a relatively common phenomenon. Chronic Spotaneous Urticaria (CSU) lasts for longer than 6 weeks.The recommended first line treatment of CSU is new generation, nonsedating H1-Antihistamines. If standard dosing is not effective, increasing the dosage up to fourfold and after this there are a number of adjunct therapy options (e.g addition of an leucotriene receptor antagonist). If this therapy failures we can use anti-IgE therapy (omalizumab)

**Objective**

We want to evaluate the effectiveness and safety of the anti-IgE therapy in CSU patients who are refractory to fourfold anti-Histamines (+/- leucotrienes antagonist) for more than 2 months.

**Methods**

Fifty patients (14: men, 36: women) with CSU (age 12 -85 years old, mean age 52 years were observed. A dose of 2x150 PFS of Omalizumab administrated per month.

**Results**

The mean age of treatment (scorad 0) was 3,064 months. None of the patients had serious side effects.

Conclusions

Omalizumab appears to be a very safety and promising therapeutic alternative for refractory chronic spontaneous urticaria.

## A71 Anaphylaxis: a life-threatening emergency, but still improperly managed

### Maria Luiza Kraft Kohler Ribeiro^1^, Ana Carolina Barcellos^2^, Hannah Gabriele Ferreira Silva^2^, Luís Henrique Mattei Carletto^2^, Marcela Carolina Bet^2^, Nathalia Zorze Rossetto^2^, Nelson Augusto Rosario^2^, Herberto Jose Chong-Neto^2^

#### ^1^Federal University of Paraná, Healthy Community, Curitiba, Brazil; ^2^Federal University of Paraná, Pediatrics, Curitiba, Brazil

##### **Correspondence:** Herberto Jose Chong-Neto

**Background**

Anaphylaxis is a life-threatening disease, need a fast recognizing and properly management.

**Objective**

To verify the level of knowledge of physicians in emergency departments in Curitiba on the management of anaphylaxis.

**Methods**

Cross-sectional study applying written questionnaire (WQ) containing questions about clinical aspects, diagnosis and treatment of anaphylaxis to physicians in public and private emergency services in Curitiba from April to July / 2016.

**Results**

There were 199 physicians invited to participate and 104 (52.3%) answer the WQ. Fifty (48.1%) working in hospitals and 54 (51.9%) in Emergency Care Units (ECU). Regarding professional experience, 20.4% was represented by physicians who graduated more than 10 years, 21.4% between 5 and 10 years of graduation and 58.2% were graduated less than five years. Sixty-five (62.5%) reported ever seen at least one case of anaphylaxis. Forty-two physicians (40.4%) answered that anaphylaxis affects at least two systems simultaneously (skin and subcutaneous tissue, respiratory, cardiovascular and gastrointestinal). Forty-five (43.3%) responded that intramuscular epinephrine is the drug and route of first choice to treat anaphylaxis, 37 (35.6%) subcutaneous epinephrine and 22 (21.1%) choice other drugs, such as corticosteroids or antihistamines, oral or injectable. Eleven (10.6%) answered that glucagon is the drug of choice for treating anaphylaxis in patients using beta-blockers drugs.

**Conclusions**

Anaphylaxis is a life-threatening condition and remains underdiagnosed and improperly managed in emergency room.

## A72 Quality of life in patients with food allergy before and after resolution

### Fernanda Valença, Marina Novaes, Mariana Gomes, Carla Seifert, Alfredo Neto, Flavia Loyola, José Rios, Tatiana Silva

#### Policlinica Geral do Rio de Janeiro, Allergy, Rio de Janeiro, Brazil

##### **Correspondence:** Marina Novaes

**Background**

Some patients which have food allergy have a bad life quality.

**Objective**

To compare the quality of life (QOL) of patients with allergy to cow's milk proteins (CMPA) before and after oral challenge test (TPO) and / or oral desensitization.

**Methods**

A prospective study was donne, using validated QOL questionnaire analysis, developed by DunnGalvin. The questionnaire was responded by patients (or guardians) who underwent negative TPO (*n* = 47), before this procedure (pre group), and another group diagnosed with CMPA who had been submitted to the oral desensitization protocol (*n* = 19) (post group) in the period May-2013 to May-2016. The items were divided in three areas: Emotional impact, Food and Social Anxiety and Dietary Restrictions (, each one with 10 to 13 questions). The responses varied from 0 to 6 (Likert scale), according impact intensity. It was added to the each domain issues set points and divided by the number of issues thereby obtaining a score of symptoms of each individual. Mean scores of individuals in each group formed the average score of each domain.

**Results**

The mean scores were obtained for each field of pre and post groups and the results were compared. Regarding the Emotional Impact, the average score was 2.69 (pre) and 1.27 (post). In Food Anxiety domain mean scores were 3.16 and 0.97, respectively, and for Social and Dietary limitations was obtained pre mean score of 3.21 and to 0.71 post.

**Conclusions**

The quality of life measures are important to evaluate the daily impact on pacient allergy and their families. The CMPA and the consequent restrictive diet profoundly affect the quality of life of affected individuals (pre group). Overcoming this condition spontaneously measured by TPO, or procedure for oral desensibilization, significantly improves QOL, with the release of social and dietary restrictions, which reduce food anxiety and emotional impact (post group).

## A73 Evaluation of life quality in patients with cow milk allergy

### Aline Neves, Marina Novaes, Fernanda Valença, Mariana Gomes, Alfredo Neto, Flavia Loyola, José Rios

#### Policlínica Geral do Rio de Janeiro, Allergy, Rio de Janeiro, Brazil

##### **Correspondence:** Aline Neves

**Background**

Some patients which have cow milk allergy have problems in life quality.

**Objective**

To evaluate the life quality of patients with diagnosis of allergy to cow’s milk.

**Methods**

A retrospective study based on questionnaire validated QOL developed by DunnGalvin and employees responded by patients (or guardians) who are allergic to cow’s milk, candidates for oral food challenge or desensitization, from May-2013 to May-2016. The questionnaires were divided into seven areas: emotional impact, symptoms of the disease, adverse disease effects, social and dietary restrictions, personal problems by food allergy, personal expectations regarding the disease and future expectations regarding the improvement of the disease. For each domain, the responses were considered low, moderate or severe criteria based on Likert Scale.

**Results**

The data generated for the fields “emotional impact” and “symptoms of disease”, 24 respondents answered that food allergies affected your life in moderation (45.28%). To “negative repercussions of the disease”, 52.83% (*n* = 28) are affected lightly and 49.05% (*n* = 26) responded that the “impact on the social and dietary restrictions” is serious. As for “personal problems caused by food allergy”, 23 respondents (43.39%) reported being affected lightly. As for “personal expectations regarding the disease and its repercussions”, 54.71% (*n* = 29) are slightly affected. Among the 53 respondents, 38 (71.69%) have only discrete “future expectations regarding the improvement of the disease.”

**Conclusions**

Life quality measures are important to evaluate the daily impact on patient lives with food allergy and determine effects of diagnosis and treatment instituted. Monitoring the quality of life of patients and their parents or caregivers is an important role in the treatment of food allergy. Life quality questionnaires have become important tools to assess the impact of the disease and take measures to minimize them.

## A74 The effect of smoking on the course of disease in newly diagnosed asthma patients: results from multicenter observational study

### Oznur Abadoglu^1^, Bilun Gemicioglu^2^, Hasan Bayram^3^, Arif Cimrin^4^, Levent Akyildiz^5^, Aykut Cilli^6^, Hakan Gunen^7^, Tevfik Ozlu^8^, Mecit Suerdem^9^, Esra Uzaslan^10^, Zeynep Misirligil^11^

#### ^1^Cumhuriyet University, Faculty of Medicine, Department of Pulmonary Diseases, Sivas, Turkey; ^2^Istanbul University, Cerrahpasa Faculty of Medicine, Department of Pulmonary Diseases, Istanbul, Turkey; ^3^Gaziantep University, Faculty of Medicine, Department of Pulmonary Diseases, Gaziantep, Turkey; ^4^Dokuz Eylul University, Faculty of Medicine, Department of Pulmonary Diseases, Izmir, Turkey; ^5^Mardin Medical Park Hospital, Department of Pulmonary Diseases, Mardin, Turkey; ^6^Akdeniz University, Faculty of Medicine, Department of Pulmonary Diseases, Antalya, Turkey; ^7^Sureyyapasa Pulmonary Diseases Hospital and Research Centre, Department of Pulmonary Diseases, Istanbul, Turkey; ^8^Karadeniz Technical University, Faculty of Medicine, Department of Pulmonary Diseases, Trabzon, Turkey; ^9^Selcuk University, Faculty of Medicine, Department of Pulmonary Diseases, Konya, Turkey; ^10^Uludag University, Faculty of Medicine, Department of Pulmonary Diseases, Bursa, Turkey; ^11^Ankara University, Faculty of Medicine, Department of Pulmonary Diseases, Ankara, Turkey

##### **Correspondence:** Oznur Abadoglu

**Background**

Smoking is one of the most significant triggers for asthma patients. There are no sufficient data on the effects of smoking in newly diagnosed asthma patients in our country.

**Objective**

To evaluate the prevalence of active smoking and factors related to its effects on newly diagnosed asthma patients from different regions of Turkey.

**Methods**

A total of 1113 newly diagnosed adult asthma patients from 122 centers are registered to the study and a questionnaire is applied between July 2012 and March 2014. Patients are categorized in three groups: smoker (S), ex-smoking (ES) and nonsmoker (NS). Demographic data are compared by means of atopy, living in urban/rural area, effects of the region, pulmonary function tests, comorbid diseases, asthma medications and additional treatments.

**Result**s

The number of patients according to the groups was as follows: S; 316, ES; 168 and NS; 629. Percentages of smoker female and male patients were similar (50,3% vs 49,7%, respectively). The number of smoker patients living in Marmara region was significantly higher than other regions (26,1%, *p*=0.00). FEV1% before bronchodilator and FEV1/FVC rate were significantly lower in S group compared to NS group (70,9+/-1,1 vs 74,3+/-0,9, *p*=0,017; 74,8+/-0,7 vs 77,7+/-0,5, *p*=0,01, respectively). There found no significant difference, in first visit, by means of disease severity and control level between three groups. The number of patients taking inhaled corticosteroid (ICS)+long acting beta2 agonist (LAB2A) treatment was significantly higher in S group compared to NS group (88,6% vs 81,7%, *p*=0,014, respectively). However, percentage of regular medication use was significantly lower in S group compared to both NS and ES group (67,6%, 81,0% and 84,5%, p=0,003, respectively). FEV1/FVC rate in patients mostly lived in urban area and FEV1 and FVC rates in patients mostly lived in urban/rural area were lower in S group compared to NS (74,2+/-0,8% vs 78,0+/-0,6%, *p*=0.00, respectively and 64,3% vs 73,7%, *p*=0,013; 71,9% vs 80,8%, *p*=0,013, respectively). Frequency of both coronary artery disease and hypertension were higher in ES group compared to S (8,4% vs 2,6%, *p*=0,001; 18,6% vs 9,3%, *p*=0,010, respectively).

**Conclusions**

We reached three remarkable results: 28% of newly diagnosed asthma patients were active smokers. Percentage of noncompliance to the treatment was higher in S group compared to other groups and ICS+LAB2A combination was the most preferred treatment in this group.

## A75 Asthma prevalence among university students in Belgrade

### Branislava Milenkovic^1^, Snezana Radic^2^, Snezana Ristic-Stojanovic^3^, A Milicevic^3^, A. Milenkovic^3^, Jelena Cvejic^1^, Jelena Jankovic^1^, Sanja Dimic-Janjic^1^, Natasa Djurdjevic^1^

#### ^1^Clinical Centre of Serbia, Clinic for Pulmonary Diseases, Belgrade, Serbia; ^2^Clinical Hospital Center “Dr Dragisa Misovic-Dedinje”, Children’s Hospital for Respiratory Diseases and TB, Belgrade, Serbia; ^3^Institute Student’s Health Care Institute, Belgrade, Serbia

##### **Correspondence:** Branislava Milenkovic

**Background**

Over the last three decades the prevalence of respiratory disease has been increasing worldwide thus increasing economic burden on the healthcare system. Recent studies have been shown that the prevalence of asthma in West European ranges from 6 to 9%, while of chronic obstructive pulmonary diseases (COPD) is 8% worldwide. Despite the large number of epidemiological studies, there are only a few studies about the prevarence of chronic respiratory diseases among students.

**Objective**

The aim of the study was to estimate the prevalence of asthma among University Students in Belgrade, Serbia.

**Methods**

We used a questionnaire based on the European Community Respiratory Health Survey (ERCHS) protocol to collect data.

**Results**

We have analysed respiratory symptoms in 5045 university students in Belgrade. There were 44.8% male and 55.2% female participants; mean age: 21.6±1.7 years for males and 21.3±1.6 years for females. There were 26.4% smokers and 3.3% ex-smokers. The most frequent symptoms were longstanding cough (15.5%) and sputum production (11.4%). Asthma attacks were reported in 2.4% of subjects and 5.0% of subjects were using asthma medications. Women reported using asthma medication more frequently than men (6.2% vs 4.3%, *p*=0.02). Allergic rhinitis was reported in 71.3% participants with asthma.

**Conclusions**

There is a high prevalence of asthma among university students in Belgrade, Serbia.

## A76 Efficiency of the SIT to the house dust mite allergens in children with atopic dermatitis

### Vladyslava Barzylovych^1^, Tetiana Umanets^2^, Anastasia Barzylovych^3^

#### ^1^Bohomolets National Medical Univercity, Pediatrician departemen, Kiev, Ukraine; ^2^Institute of Pediatrics, Obstetrics and Gynecology, Drug Allergy Center, Kiev, Ukraine; ^3^Oberig clinic, Pediatrician department, Kiev, Ukraine

##### **Correspondence:** Vladyslava Barzylovych

**Background**

Nowadays allergen specific immunotherapy (SIT) is widely used in the treatment of asthma and allergic rhinitis. PubMed analysis shown that the role of SIT in the treatment of atopic dermatitis (AD) is still uncleare and need more reserch especially in childhood.

**Objective**

The aim of the study was to determine role of SIT using house dust mite (HDM) allergens in the treatment of AD in children.

**Methods**

Under our supervision were 23 3-years old children with moderate atopic dermatitis. All children received treatment according to the WAO Guideline including avoidance of causative food allergens and local therapy with insufficient results. All children had isolated sensitization to the Derp1 or/and Derp2 (ImmunoCAP). The children were divided in two groups. In the second group (12 children) the treatment was supplemented with SIT to the HDM. Children were under observation up to two years.

**Results**

During first 6 months there were no significant difference in the severity and the frequency of exacerbations of AD in two groups.

In the analysis of long-term results (after 1,5-2 year of treatment) there are significant different in severity of AD: (3 mild and 8 moderate in the 1-st group and 7 mild, 2 moderate and 3 children without clinical signs in the 2-nd group).

Also, in the 2-nd group the presentation of dermato respiratory syndrome was significantly lower (8 children with bronchial asthma in the 1-st and 3 children in the 2-nd group).

**Conclusions**

The role of SIT to the HDM in the treatment of AD need more investigation and could be considered like prophylactic of dermato respiratory syndrome realization.

## A77 Melatonin alters nitric oxide production by IFN-gamma/Vitamin D3 stimulated peripheral blood mononuclear cells (PBMC) from healthy adults and patients with allergy/asthma

### Karyn Winkler^1,2^, Jessica Margarinos^3^, Dylan Martin^3^, Maja Nowakowski^1,4^, Rauno Joks^1,5^

#### ^1^State University Of New York Downstate Medical Center, Center for Allergy and Asthma Research, Brooklyn, New York, NY, USA; ^2^State University Of New York Downstate Medical Center, Department of Pediatrics, Brooklyn, New York, NY, USA; ^3^State University Of New York Downstate Medical Center, College of Medicine, Brooklyn, New York, NY, USA; ^4^State University Of New York Downstate Medical Center, Department of Pathology, Brooklyn, New York, NY, USA; ^5^State University Of New York Downstate Medical Center, Department of Medicine, Brooklyn, New York, NY, USA

##### **Correspondence:** Karyn Winkler

**Background**

We have previously reported that low levels of melatonin increased the amounts of nitric oxide produced in vitro by peripheral blood mononuclear cells (PBMC) from healthy adults.We related these data to our earlier observation of a seasonal increase in angioedema in the summer months, peaking in June. Melatonin, a factor responsible for sleep-wake cycles, is decreased with long summer days. As melatonin is known to suppress eNOS, which is upregulated by bradykinin, we hypothesized that decreased melatonin is associated with increased nitric oxide production from stimulated PBMC.

**Methods**

Blood was drawn from healthy subjects (*n*=6) between 8 - 11 AM and PBMC (3x106/ml) were stimulated with bradykinin (100 nmol/ml), ± hIL-15 (1 μg/ml), ±IL-18 (1 ug/ml), +/-IFN-gamma (10 ng/ml)/ Vitamin D3 (20 pmol/ml) for five days, and then incubated for 4 hrs in the absence or presence of recombinant human melatonin(1 pmol/m). Nitric oxide levels in the culture supernatants were determined using Griess reaction.

**Results**

Stimulation with bradykinin, hIL-15 and hIL-18 did not increase NO production above baseline (base: 0.61±0.76 μM: bradykinin+hIL-15+hIL-18: 0.63±0.76 μM, *p*=0.62). Addition of melatonin did not alter NO production (0.59±0.76μM) (*p*=ns). However, addition of melatonin to PBMC cultures stimulated with IFN-gamma and Vitamin D3 resulted in significant increase in NO: (1.84±0.21 μM) (*p*<0.01). When PBMC were obtained from patients with allergy/asthma, the effects of melatonin depended on the time of blood collection and PBMC isolation. PBMC isolated in the morning (9-10 AM), after overnight period of sleep-associated elevation in endogenous melatonin levels, did not respond to added melatonin with any increase in NO production (below detection). In contrast, when PBMC were isolated from blood collected in the afternoon (5-6 PM), NO production was high in cultures stimulated with bradykinin and with melatonin (1.45± 0.16 μM and 1.43± 0.07 μM, respectively).

**Conclusions**

Prolonged, low daytime levels of melatonin increase nitric oxide production from Interferon-gamma/Vitamin D3 stimulated leukocytes and may contribute to seasonal angioedema. Adding melatonin to PBMC isolated from allergy/asthma subjects in the morning did not cause any increase in nitric oxide production above that observed in the presence of bradykinin, hIL-15 + hIL-18, and Interferon-gamma/Vitamin D3. This is likely to be an effect of increased overnight endogenous melatonin levels in vivo.

## A78 Eosinophilic Esophagitis: review of the Israeli experience in an allergy reference center

### Miguel Stein^1^, Tsili Zangen^2^, Olga Bernadsky^3^, Mona Boaz^4^, Gratiana Hermann^5^, Yoram Faitelson^1^, Ilan Dalal^1^, Rachel Aviv^1^, Olga Kuperboim^1^, Larisa Ramichanov^1^, Efrat Broide^6^, Raanan Shamir^7^, Noam Zevit^7^, Ron Shaoul^8^, Alex Fich^9^, Arie Levine^2^

#### ^1^E. Wolfson Medical Center, Allergy/Immunology Unit, Holon, Israel; ^2^E. Wolfson Medical Center, Pediatric Gastroenterology, Holon, Israel; ^3^E. Wolfson Medical Center, Pathology, Holon, Israel; ^4^E. Wolfson Medical Center, Biostatistics, Holon, Israel; ^5^Assaf Harofeh, Pathology, Zerifin, Israel; ^6^Assaf Harofeh Medical Center, Pediatric Gastroenterology, Zerifin, Israel; ^7^Schneider Children, Pediatric Gastroenterology, Petach Tikva, Israel; ^8^Rambam Medical Center, Pediatric Gastroenterology, Haifa, Israel; ^9^Soroka Medical Center, Gastroenterology, Beer Sheva, Israel

##### **Correspondence:** Miguel Stein

**Background**

Eosinophilic Esophagitis (EoE) is a clinicopathologic condition characterized by esophageal dysfunction and eosinophilic inflammation. Identification of food allergens is crucial for the management, and delaying diagnosis can lead to esophageal strictures and morbidity.

**Objective**

A descriptive analysis of EoE in Israel, to increase awareness for an early diagnosis, and the proper testing for a better management.

**Methods**

Eighty six consecutive patients derived for EoE allergic evaluation, were tested for at least 30 food allergens by skin prick (SPT) and Atopy Patch (APT) Testing. Clinical history was taken in each individual or their parents, for allergic and gastrointestinal complaints, and pathological data were revised. Patients with full EoE diagnostic criteria, tested and interviewed in the allergy clinic, were included in the analysis.

**Results**

Fourty five EoE patients were available for the analysis, 26 (57.8%) children’s and 19 (42.2%) adults, (age 2.5-17 and 18-67 years old, respectively). Patients were predominantly males 36 (80%) and atopics 34 (76%). The first EoE presenting symptom was at age 6 (1-16) and 23.4 (5-59) years old for children’s and adults, respectively, being principally vomiting (45%), abdominal pain (38%), food impaction (38%), dysphagia (24%) and failure to thrive (24%) for kids, and dysphagia (68%), partial or complete food impaction (68%) and vomiting (58%) for adults. Delay in the diagnosis was between 0.3-13 (average 3.5) years for children’s and 0.9-23 (average 8) years for adults.

Food related anaphylaxis was found in 7 (16%) individuals. Positive food SPT was observed in 25/44 (56.8%) most commonly to peanuts, tree nuts and soy. Positive APT observed in 24/38 (63%) individuals, most commonly to fish, peanuts wheat and chicken. The combination of SPT+APT could identify food allergens in 35/44 (79.5%) individuals. Interestingly all the non-atopic EoE patients had a positive APT.

**Conclusions**

•EoE is not uncommon in Israel and probably under-diagnosed.

•There is a big delay in the diagnosis of EoE, from 3 to 8 years in the pediatric and adult population.

•The most common presenting symptoms for pediatric EoE are vomiting, partial or complete food impaction and abdominal pain.

•The most common presenting symptoms for adult EoE are dysphagia, partial or complete food impaction and vomiting.

•Different food allergens are found by SPT and APT, and the best yield is obtained with the combination of both (IgE & Non-IgE mediated testing).

•All non-atopic EoE should have an APT examination.

## A79 STAT3 mutation presenting as autosomal dominant common variable immunodeficiency (CVID), a novel presentation

### Adi Ovadia, Yael Dinur Schejter, Chaim Roifman

#### Hospital for sick children, Immunology and Allergy, Toronto, Canada

##### **Correspondence:** Adi Ovadia

**Background**

STAT3 mutations have been traditionally associated with hyper-IgE syndrome. While loss of function mutations present in a typical fashion, STAT3 gain of function mutations reported recently lead to lymphoproliferation and early-onset autoimmune disease.

**Objective**

We report of a father and a son with STAT3 gain of function mutation presenting as a common variable immunodeficiency (CVID).

**Case report**

A 47-year-old man initially presented at 3.5 years with recurrent chest infections and normal immunoglobulin titers. At the age of 7.5 years, he developed ITP and lymphadenopathy. Immunoglobulin levels were once again normal including IgE. Lymph node biopsy demonstrated reduced plasma cells. At the age of 17 years, during an admission for complicated pneumonia, he was noted to have hypogammaglobinemia and antibody deficiency, and was subsequently started on IVIG replacement. From the age of 35 years, he gradually deteriorated and developed respiratory as well as liver failure ultimately requiring double lung and liver transplant, which have completely reversed his disease course. He is currently well. His son presented at the age of 15 months with lymphadenopathy without hepatosplenomegaly. His initial investigations revealed normal immunoglobulin titers with variable antibody response to vaccines. He was clinically well and suffered mainly from upper respiratory tract infection but no significant bacterial sinopulmonary infections. At the age of 7 years, he was noted for the first time to have hypogammaglobulinemia and a complete loss of specific antibodies, and was therefore given IVIG replacement therapy. Currently, he is 14 years old, and remains well with no autoimmunity.Whole-exome sequencing identified heterozygous STAT3 mutation c.2147 C>T predicting Threonine to Methionine amino acid change at position 716. This mutation has been previously reported as a gain of function mutation.

**Conclusions**

STAT3 gain of function mutations can present as autosomal dominant CVID.

**Consent**

The authors received informed consent from the first patient and second patient’s guardian/parent.

## A80 The role of C1 inhibitor in neuroimmune disease

### Isaac Melamed

#### ImmunoE Immunology, Centennial, CO, USA

**Background**

The term neuroimmune disease (NID) refers to a group of illnesses that are the result of acquired dysfunction cross talk between the immune system and the nervous system and often result in lifelong disease and disability. Symptoms may include cognitive changes, abdominal symptoms, and extreme fatigue. The diseases usually follow an infection or flu-like symptoms that never resolve. One of the pathogens linked to the neuroimmune presentation is Lyme disease. The link between Lyme disease and neurocognitive disease has been a focus of research and multiple therapeutic approaches, but no clear mechanism has been identified.

**Objective**

Here we present 3 case studies in patients with NID.

**Methods**

Our immune work up was divided into 3 layers: epigenetic infectious activation, immune dysregulation, and neuroimmune presentation. Epigenetic triggers included positive IgM western and PCR for Lyme. Immune dysregulation was characterized by IgG3 subclass deficiency, low response to T cell antigens, decrease activation of toll like receptor (TLR)-3, low memory B cells, and low C1 INH. Neuroimmune markers were anti-GAD and anti-68 kDa, as well positive ANA and phospholipid antibodies. Our patients presented with a complex of neurological symptoms, including low C1, low IgG3, a decrease in TLR 3, and evidence of neuroimmune auto-antibodies. All patients were treated with IVIG or SCIG 600-800 mg/kg for 3 week intervals.

**Results**

With this treatment plan, we were able to see a major reversal in the incidence of infections, but no major change in the neurological presentation or the neuroimmune response to the disease. In 2 patients, we added C1 inhibitor (C1 INH) therapy (100 units twice a week) to the treatment regimen. In these patients who received C1 INH and IVIG therapy, we were able to see some neurological improvement as well a reduction in neuroimmune markers.

**Conclusions**

Further studies are needed; however, the synergistic effect of IVIG and C1 therapy may provide an optimal therapy in the future.

## A81 CD48 on blood leukocytes and in serum of asthma patients varies with severity

### Roopesh Singh Gangwar^1^, Yael Minai-Fleminger^1^, Mansour Seaf^1^, Amichai Gutgold^1^, Aarti Shikotra^2^, Anoop Chauhan^3^, Stephen Holgate^4^, Peter Bradding^2^, Peter Howarth^4^, Ron Eliashar^5^, Neville Berkman^6^, Francesca Levi-Schaffer^1^

#### ^1^The Hebrew University of Jerusalem Institute For Drug Research, Portsmouth, Israel; ^2^University of Leicester Institute for Lung Health, Leicester, UK; ^3^Portsmouth Hospitals NHS Trust Respiratory and General Medicine, Portsmouth, UK; ^4^Southampton General Hospital NIHR Respiratory Biomedical Research Unit, Southampton, UK; ^5^Hadassah Hebrew University Medical Center Department of Otolaryngology/Head and Neck Surgery, Jerusalem, Israel; ^6^Hadassah Hebrew University Medical Center Institute of Pulmonary Medicine, Jerusalem, Israel

##### **Correspondence:** Roopesh Singh Gangwar

**Background**

CD48 is a membrane receptor (mCD48) on eosinophils and mast cells and exists in a soluble form (sCD48). CD48 has a pivotal role in murine asthma and in the proinflammatory interactions of mast cells with eosinophils via its ligand CD244. Thus CD48 might be important in human asthma.

**Objective**

To investigate the expression and role of mCD48 on peripheral blood leukocytes and sCD48 in serum in asthmatic patients with varying disease severity.

**Methods**

Two separate cohorts (IL and UK) comprising mild, moderate, and severe asthma and healthy volunteers were evaluated for blood leukocyte mCD48 expression and sCD48 in serum. Asthmatic bronchial biopsies were immunostained for CD48. sCD48 effect on CD244-dependent eosinophil activation was evaluated.

**Results**

Eosinophils mCD48 expression was significantly elevated in moderate while downregulated in severe asthma. mCD48 expression on B, T, NK cells and monocytes in severe asthma was significantly increased. sCD48 levels were significantly higher in mild while reduced in severe asthma. sCD48 optimal cut-off values for differentiating asthma from health were identified as >1482 pg/ml (IL) and >1619 pg/ml (UK). In asthmatic bronchial biopsies mCD48 was expressed predominantly by eosinophils. sCD48 inhibited anti-CD244 induced eosinophil activation.

**Conclusions**

mCD48 and sCD48 are differentially expressed in the peripheral blood of asthma patients of varying severity. sCD48 inhibits CD244-mediated eosinophil activation. These findings suggest that CD48 may play an important role in human asthma.

## A82 Prognostic value of exhaled nitric oxide monitoring for asthma prognosis in Korean children and asthma friendly school

### Sung-il Woo

#### Chung-buk National University Hospital Department of Pediatrics, Choeng-ju, South Korea

**Background**

The fractional exhaled nitric oxide (FeNO) measurements are useful method of diagnosing asthma and monitoring tool for airway inflammation.

**Objective**

We sought to evaluate the yield of FeNO monitoring on graft concept in asthma friendly school for asthma exacerbation and prognosis, so that could identify the usefulness of FeNO in children.

**Methods**

One hundred and seventy element school student aged 7-12 years with agreement of parents in elementary school were included. Children were evaluated using FeNO measurement, questionnaire of asthma symptom and control, history taking of asthma treatment, skin prick test, sprirometries.

**Results**

One hundred fifty nine student (93.5%) had completed follow up and monitoring of FeNO at each season over 1 year. Twenty five children had asthma exacerbation or uncontrolled asthma. These children had higher FeNO level than those without asthma exacerbation or uncontrolled asthma. (27.7 ppb vs 22.7 ppb, *P*<0.05) There was also significant difference of maximal level of FeNO. (*P*<0.05). Among these children with inhalant allergen sensitization, mean of maximal FeNO was 34ppb. Fifteen children had history of medical treatment for asthma exacerbation and seven children had absence of school associated with asthma exacerbation.

**Conclusions**

FeNO monitoring has prognostic value of asthma monitoring. It can predict asthma exacerbation of children who has higher level of FeNO, especially who has atopic asthma or aeroallergen sensitization.

## A83 Human Epidydim Protein 4 may be a target for gene therapy in patient with high grade serous carcinoma of the ovary

### Betul Celik^1^, Tangul Bulut^1^, Arzu Didem Yalcin^2^

#### ^1^Antalya Training Hospital Pathology, Antalya, Turkey; ^2^Antalya Training Hospital Allergy and Immunology, Antalya, Turkey

##### **Correspondence:** Arzu Didem Yalcin

**Background**

Human epididymis 4 (HE4) protein belongs to whey acidic 4-disulfide center protein family (ref-5). The protein shows characteristics of a secretory protein, with an acidic and cysteine-rich polypeptide. It is a protease inhibitor and involved in the innate immunity defense of the respiratory tract and nasal cavity. Formerly found in epididymis, it is now shown that serum level is the most predictive marker detecting adnexial malignancy.

**Objective**

The aim of this study was to evaluate the presence of human epididymis secretory protein 4 (HE4) in patient with high grade serous carcinoma of the ovary as to whether it may be a potential candidate for gene therapy in ovarian cancer treatment.

**Methods**

A total of 31 patients with a diagnosis of high grade serous ovarian carcinoma were enrolled. All patients underwent surgical resection. Final original diagnosis had been reached by histopathological feature of the tumor and by combined immunohistopathological stains using CA125, and p53. Concomitant tumor pathology blocks were examined after HE4 immunohistopathological staining.

**Results**

Of the tumor tissues studied, HE4 immunostaining was seen in majority of the cases (28 out of 31 cases) (90,32%). Moreover, neither CA125 nor p53 results were available in six cases based on pathology reports, in which HE4 expression was observed in 5 cases.

**Conclusions**

Immunohistochemical staining pattern of HE4 protein 4 is expressed in the majority of the high grade serous ovarian carcinoma and it may be a potential candidate for gene therapy as well as it is a good immune marker for detecting high grade serous ovarian carcinoma.

## A84 Is Human Epididymis Protein 4a (HE4) a reliable screening biomarker for detecting lung carcinoma patients.

### Betul Celik^1^, Arzu Didem Yalcin^2^, Tangul Bulut^1^

#### ^1^Antalya Training Hospital Pathology, Antalya, Turkey; ^2^Antalya Training Hospital Allergy and Immunology, Antalya, Turkey

##### **Correspondence:** Arzu Didem Yalcin

**Background**

Human epididymis 4 (HE4) protein belongs to whey acidic 4-disulfide center protein family. The protein shows characteristics of a secretory protein, with an acidic and cysteine-rich polypeptide. It is a protease inhibitor involved in the innate immunity defense of the respiratory tract and nasal cavity. HE4 has been detected in the malignant pleural effusions of patients with lung carcinoma. As a serum and pleural fluid biomarker, it has very high sensitivity (43.8-69.4%) and specificity (78.5-95.0%) for lung cancer.

**Objective**

The value of HE4 among lung carcinoma subtypes are limited. The aim of this study was to investigate the expression of human epididymis protein 4 (HE4) in small cell lung cancer (SCLC) and lung adenocarcinoma cases.

**Methods**

Fifty-four patients with a diagnosis of SCLC and twenty-one adenocarcinoma (AC) from a bronchoscopic biopsy were enrolled in the study. The tumors were classified as SCLC or AC based on histology and immunohistopathology for CD56 and/or synaptophysin, Chromogranin, thyroid transcription factor-1 (TTF-1), CK7 immunostains. Representative blocks were immunostained with HE4.

**Results**

Using HE4 immunostain, the majority (90.1%) of the SCLC cases were devoid of HE4. Only the 9.9% cases were positive for HE4 immunostain. HE4 positive staining was focal and moderate trough out the tumor tissue. On the other hand, fifteen AC cases (71.4%) were positive with HE4, whereas 6 cases (28.6%) were negative with HE4.

*Conclusions*

This study showed that HE4 is expressed in the majority of the AC but it is less frequently expressed in SCLC, that greatly limits the utility of HE4 as a screening tool.

## A85 Off-label and concurrent use of an anti-asthma agent in the treatment of a chronic kidney disease: a case report

### Luiz Querino Caldas

#### Federal Fluminense University Pathology, Niterói, Brazil

**Background**

Since 2004, a 37-year-old female has been referring to our clinic for multiple episodes of respiratory allergy including rhinitis, sinusitis, cough, epistaxis, dyspnea, amongst other symptoms. Ten years ago after severe episode of glomerulonephropathy postinfection she was reported with hematuria, hypertension, edema and renal insufficiency that led her to hemodialysis since then. Due to a lack of recovery of the renal function, her chronic kidney disease (CKD) progressed gradually to a Stage V, being her glomerular filtration rate lower than 15ml/min. In the meantime, her allergic episodes including pruritus have been treated with antihistamine drugs as well as immunotherapy was used for a while, although antileucotriene agents had never been under the scope of the treatment. In 2009, she had a severe bone mineral disorder developing a spontaneous and consecutive rupture of both Achilles tendons. In uremic patients and on the patient’s lack of corticosteroid or fluoroquinolone use, it seems that occurred to secondary hyperparathyroidism which was the predisposing factor in this patient. A year ago, she fractured her both femurs and seizure episodes outcome. The later may be related to the high parathyroid hormone (PTH) serum level, which resulted in osteolytic bone resorption and at the tendon insertion site. Additionally, she presented perforating dermatosis in both arms with hyperpigmented macules areas prior involvement that were related to elevating plasma level of β-melanocyte-stimulating hormone.

**Results**

Treatment and prevention of such tendon ruptures included early surgical repair and control of secondary hyperparathyroidism, by the use of vitamin D analogs, although she remained hypercalcemic and hyperphosphatemic occasionally. Objective: Lately, for allergic purposes, she was initially treated with sodium montelukast (5mg) and as soon as this dose increased to 10mg/day she had slight improvement but significant (p <0.05)in her creatinine clearance (Cockcroft-Gault Index -10.86) with the serum levels of glucose, calcium, potassium, urea post-dialysis, creatinine and phosphates a rather stable without significant changes from normal values during the last 6 months. Supposedly, CRD is associated to production of reactive oxygen species and cytokine release that may be prevented by the concurrent long term use of a CysLTd1 receptor antagonist montelukast.

**Conclusion**

This activity can be attributed to its ability to inhibit neutrophil and leucocyte apoptosis by balancing the cell oxidative status and regulating the proinflammatory mediators production. A renal transplantation was thus so far postponed as for an alternative treatment.

Consent to publish

The patient has signed the Term of Consent freely and due explained.

## A86 Rush venom immunotherapy in children

### Ronit Confino-Cohen, Yossi Rosman, Arnon Goldberg

#### Meir Medical Center Allergy and Clinical Immunology Unit, Kefar Saba, Israel

##### **Correspondence:** Yossi Rosman

**Background**

Rush venom immunotherapy (VIT) is highly effective in Hymenoptera venom allergy. Still, specific data regarding its safety and efficacy in children are rather sparse.

**Objective**

To better evaluate the safety and efficacy of rush VIT in this specific age group.

**Methods**

Children younger than 16 years with systemic reaction to insect sting involving, at least, one body system other than skin and children aged 16-18 years old with any kind of systemic reaction were offered conventional or rush VIT with a build-up phase that lasted 3 days.

**Results**

Eighty-four out of 127 children chose to receive rush VIT. Seventy of them were allergic to bee venom (BV) only. There was no difference between the children receiving rush or conventional VIT in the incidence of systemic reactions during the build-up phase (19% and 23.2%, respectively), nor was there any difference with regard to the severity of these reactions. Efficacy was improved with rush VIT, as reflected by higher number of patients achieving the 100 mcg maintenance dose with the primary protocol (83/84 patients, 98.8% and 39/43, 90.7%, respectively, P=0.04).

**Conclusions**

Rush VIT in children is as safe and more efficient when compared with conventional VIT.

## A87 CD48 as a potential biomarker for allergic and non-allergic asthma

### Oded Breuer^1^, Roopesh Singh^2^, Mansour Seaf^2^, Ahlam Barhoum^2^, Eitan Kerem^1^, Francesca Levi-Schaffer^2^

#### ^1^Hadassah Hebrew University Medical Center Pediatric Pulmonology, Jerusalem, Israel

^2^The Hebrew University of Jerusalem Department of Pharmacology and Experimental Therapeutics, Institute for Drug Research, Faculty of Medicine, Jerusalem, Israel

##### **Correspondence:** Oded Breuer

**Background**

CD48 is a member of the CD2 subfamily of immunoglobulin-like receptors, present as membrane receptor on hematopoietic cells and as a soluble (sCD48) form in serum. CD48 was found to be involved in allergic eosinophilic airway inflammation and shown to be a potential target for the suppression of asthma in mice. The full significance of CD48 in patients with asthma has not yet been evaluated.

**Objective**

To evaluate the expression of sCD48 as a possible biomarker for asthma and correlate its expression with other disease markers.

**Methods**

Volunteer patients completed an asthma and allergy questionnaire, performed spirometry, methacholine (Mch) challenge test, a common allergen skin prick test, a complete blood count. Moreover sCD48,IgE, IL17A and IL33 levels were measured in their serum. Asthma was defined as positive Mch challenge test or a 15% increase in FEV1 post bronchodilator administration in symptomatic individuals. Allergy was defined as positive skin test or IgE levels > 200 IU/L in symptomatic individuals.

**Results**

137 individuals participated in the study with a mean age 37.9 ± 19.3 years (range 5-79), 47 (34%) were male and 82 (60%) were diagnosed with asthma of which 53 (64%) was allergic asthma. Levels of sCD48 were significantly elevated in asthmatic patients but especially in the non-allergic asthmatic patients when compared to control (1468±360 pg/ml 1551±374 pg/ml vs. 1298± 315 pg/ml, p<0.01). IL17A and IL33 levels were significantly elevated in allergic and non-allergic asthmatics when compared to control: IL17A; 100±75 pg/ml and 132±86 pg/ml vs. 59±33 pg/ml, p<0.01, respectively; and IL33; 169±140 pg/ml and 175±35 pg/ml vs. 57±36 pg/ml, p<0.01, respectively). However, there was no correlation between sCD48 levels and, eosinophil count, IgE, IL17A or IL33 levels in asthmatic or non-asthmatic patients.

**Conclusions**

sCD48 is possibly an independent biomarker for asthma. The significance of elevated sCD48 especially in non-allergic asthma is incompletely understood but may represent nonspecific airway inflammation. Additional studies are required for understanding the role of sCD48 in airway disease and non-allergic asthma.

## A88 Immunological criteria for the activity of allergic inflammation and prognosis of infectious complications in children with atopic dermatitis

### Tatiana Slavyanskaya, Revaz Sepiashvili

#### People’s Friendship University of Russia Allergy & Immunology, Moscow, Russia

##### **Correspondence:** Revaz Sepiashvili

**Background**

The purpose of the study is to develop criteria for prognosis of infectious complications in children (Ch) with moderate to severe atopic dermatitis (AD).

**Objective**

The object of the study was 94 Ch aged 5-17 years with IgE-mediated AD in remission with duration of the disease of 1-15 years. Clinically, the AD had multiple and extensive inflammation with exudation or infiltration, lichenification, excoriation and severe itching, which led to the accession of secondary infection. A control group included 30 practically healthy Ch.

**Methods**

All patients underwent clinical and immunological and allergological studies. SCORAD was evaluated on a scale of severity of clinical symptoms. Particular attention was paid to the phagocytic immunity.

**Results**

The most distressing symptoms were pruritus (3.69±0.22), xerodermia (3.34±0.23), physical discomfort (3.13±0.14) and sleep disorders. The signs of immune imbalance in patients with AD: reductions of phagocytic activity of neutrophils (PhAN) - phagocytic index (PhI) and the phagocytic number (PhN). In moderate and severe AD serum cytokine levels and cytokine regulatory indices were respectively: IL-4: 55.2±4.2 pg/ml; IL-13: 39.4±4.9 pg/ml; IFNγ: 11.4± 1.6 pg/ml; RIFNγ/ IL-4: 0.21±0.06; RIFNγ/IL-13: 0.29±0.05 (P<0.001 vs. controls). Both systemic and local IL-13 levels depended more on disease severity, its extent and presence of secondary infection, rather than on IL-4 and IFNγ concentrations. Severe and complicated AD presented with serum IgE of 496.4+62.12 IU/ml and IL-13 of 1295.41±29.8 pg/ml during exacerbation, and IgE of 484.8+60.14 IU/ml during remission. IL-13 and IgE correlation index in saliva was r = +0.67, and in serum r = +0.76. The SCORAD index before treatment of moderate AD was 42,57±3.41 and severe AD - 48,2±5.7 points. It was found that immunocompromised Ch with AD and impaired function of the PhAN, the inclusion of allergen-specific immunotherapy (AIT) in the comprehensive treatment (CT) in 78% of cases led to the development of pyodermia and joining of viral infection. that complicates the course of the disease. In the group of Ch with AD, which in CT plan has been included AIT and immunomodulator that activates PhAN, there were no complications or worsening of the clinical course of AD.

**Conclusions**

Thus, monitoring of the blood levels of PhI and PhN in IgE-mediated AD predicts the development of infectious complications in Ch with AD. Monitoring of cytokine profile along with other parameters during treatment course has certain diagnostic value in determination of localization, severity, degree and activity of allergic inflammation.

## A89 Programmed death-1 expression on T subsets in patients with atopic dermatitis during autologous activated T cell immunotherapy

### Elena A. Blinova^1^, Ekaterina A. Pashkina^1^, Marina I. Leonova^2^, Vera M. Nepomnyaschikh^2^, Darya V. Demina^2^, Vladimir A. Kozlov^1^

#### ^1^RIFCI laboratory of clinical immunopathology, Novosibirsk, Russia; ^2^Clinic of Immunopathology RIFCI allergological department, Novosibirsk, Russia

##### **Correspondence:** Elena A. Blinova

Predisposition to allergic diseases has been associated with dysfunction of regulatory cells. PD-1/PD-L1 pathway is involved in the generation of regulatory T-cells. Induction of the anti-ergotypic response during immunotherapy with autologous cells allows to eliminate activated T cells, regardless of their antigenic specificity. This response acts against activation markers (ergotopes).

The purpose of this study was to determine the expression of PD-1 in different subsets of T cells in patients with atopic dermatitis during T-cell immunotherapy.

The study was approved by the ethical commission of RIFCI, Russia. During immunotherapy patients was administered subcutaneously with activated anti-CD3 antibody+IL-2 T cells (30*10^6 cells per dose) according to scheme: 1 injection once a week - 4 times, then 1 injection per month - 6 times. Efficiency of treatment was determined as a reduction of clinical symptoms, it was about 80%.

Patients with AD were characterized by significant increase (*p* <0,05, Mann-Whitney test) in amount of peripheral blood Treg and rise expression of PD-1 on it compared to donors. Significant differences in expression of PD-1 have been identified on CD4+ T-cells, but not on CD8+ cells. Thus, in the peripheral blood of patients a large number of CD4+ cells bearing activation marker PD-1 circulated, which is consistent with the pathogenesis of these diseases. The increased Treg population indicates the absence of defects in the Treg generation; however, it is not clear how to relate the increase in the PD-1 expression on Treg and their functional activity.

On 10th injection CD4+ cells did not show significant differences of PD-1 expression compared to the level before vaccination. There was a decrease in PD-1 expression on CD4+, CD8+ T-cells expressed CD25, and CD4+25high regulatory cells. The cell number in the investigated subpopulations before and after immunotherapy was not significantly change. Observed alterations possible indicate the reduction of the number of activated T-cells, which involved in allergic reaction, as a result of anti-ergotypic response. PD-1 pathway may negatively regulate its own functions: PD-1 signaling may result in reduced PD-L1 expression and to downregulate Treg development and/ or function.

The reported study was funded by RFBR, according to the research project No. 16-34-60167 mol_a_dk.

## A90 ROCK serve as negative regulators of degranulation of human and mouse eosinophils

### Revital Shamri^1^, Kristen M. Young^2^, Peter F. Weller^2^

#### ^1^Hebrew University of Jerusalem School of Pharmacy, Jerusalem, Israel; ^2^Beth Israel Deaconess Medical Center, Harvard Medical School Department of Medicine, Boston, MA, USA

##### **Correspondence:** Revital Shamri

**Background**

Eosinophils are innate immune granulocytes known for their cytotoxic effector functions against pathogens and their involvement in host tissues damage in allergic diseases. A unique character of eosinophils is their cytoplasmic granules which contain many preformed cationic cytotoxic proteins that are not found elsewhere. Among these proteins are eosinophil-associated RNases (EARs), which have been shown to be involved in host defense, tissue remodeling, immunity regulation and pathology in eosinophil-associated diseases. Rho-associated coiled-coil forming kinases (ROCK) are known downstream effectors of Rho A, and are involved in cytoskeletal reorganization, stress fiber and focal adhesion formation. ROCK inhibition was found to inhibit eosinophil chemotaxis and migration into lung during airway inflammation. However, ROCKs role in granule content deposition (i.e. degranulation) from eosinophils was not addressed.

**Objective**

To examine the role of ROCK in eosinophil degranulation.

**Methods**

ROCKI and II involvement in CCL11-stimulated degranulation was examined by treatment of human or mouse eosinophils with the highly potent and selective cell permeable inhibitor Y27632, that blocks ROCKI and II with similar potency. Degranulation was assessed by measuring RNase activity of secreted granule-associated RNases.

**Results**

Effective concentrations of Y27632, that blocked chemotaxis of murine and human eosinophils, did not inhibit CCL11-mediated degranulation and even increased it compared to vehicle. In addition, the inhibitor Y27632 also increased spontaneous eosinophil degranulation. Moreover, degranulation enhancement by Y27632 was not due to an effect on actin polymerization or surface expression of CCL11 receptor, CCR3. It was previously shown by our studies and others that beta-2 integrin mediated spreading is essential for CCL11-mediated degranulation of eosinophils. Along these lines, we found that Y27632 pretreatment of human eosinophils increased the surface expression of CD11b, as well as its active conformation in the presence and absence of CCL11. Similar results with CD11b surface expression were also found with mouse eosinophils.

**Conclusions**

Our results suggest that ROCK are playing a negative regulatory role in degranulation of human and mouse eosinophils and that ROCK’s inhibitory effect on degranulation is probably by increasing CD11b expression and activation state.

## A91 Purinergic signaling is involved in the beneficial effects of aerobic exercise in a model of HDM-induced asthma

### Rodolfo de Paula Vieira^1^, Manoel Carneiro Oliveira-Junior^1^, Nilsa Regina Damasceno-Rodrigues^2^, Fernanda Magalhães Arantes-Costa^3^, Milton Arruda Martins^3^, Ana Paula Ligeiro Oliveira^1^

#### ^1^Nove de Julho University Laboratory of Pulmonary and Exercise Immunology, Sao José dos Campos, Brazil; ^2^School of Medicine, University of Sao Paulo Pathology (LIM 59), Sao Paulo, Brazil; ^3^School of Medicine, University of Sao Paulo Clinical Medicine (LIM 20), Sao Paulo, Brazil

##### **Correspondence:** Rodolfo de Paula Vieira

**Background**

Purinergic signaling has emerged as a central pathway involved in the asthma pathophysiology, occurring mainly through the accumulation of nucleotides in the extracellular milieu, resulting in activation of purinergic receptors. On the other side, regular low to moderate intensity aerobic exercise is shown to reduces asthma phenotype.

**Objective**

Investigate if the beneficial effects of aerobic exercise occur due to modulation of purinergic signaling.

**Methods**

Forty C57Bl/6 mice were equally distributed in Control, Exercise, house dust mite (HDM) and HDM+Exercise groups. HDM animals received intra-tracheal (days 0, 7, 14, 21, 28, 35, 42) HDM (dermatophagoydes pteronyssinus - 100ug/mouse) and were subjected to low intensity aerobic treadmill physical training (AT) (for evaluation of therapeutic effect), from day 17 for 4 weeks, 5x/week, 1hour/session. Twenty-four hours after the last training session and challenge with HDM, pulmonary inflammation was assessed through bronchoalveolar lavage (BAL) and histomorphometrical analysis, airway remodeling (collagen and elastic fibers deposition, mucus production), airway hyperresponsiveness (AHR) to methacholine (MCh), cytokines levels in BAL and in re-estimulated lymphnodes, splenocytes and bone marrow cells. Adenosine triphosphate (ATP) levels in BAL was also measured. In addition, quantitative analysis of expression of P2X7 by epithelial cells and by peribronchial leukocytes were done.

**Results**

The results demonstrated that AT in HDM mice resulted in reduced number of total cells (*p*<0.001), eosinophils (*p*<0.001), neutrophils (*p*<0.05), lymphocytes (*p*<0.05), macrophages (*p*<0.05) in BAL, in the number of eosinophils (*p*<0.001), neutrophils (*p*<0.01), lymphocytes (*p*<0.001) and macrophages (*p*<0.01) in airways wall. AT also reduced HDM-induced airway remodeling, as demonstrated by reduced accumulation of collagen fibers (*p*<0.01), elastic fibers (*p*<0.01) and mucus production (*p*<0.01). AHR was also reduced in HDM+exercised mice for 6,25 mg/mL MCh (*p*<0.01), 12,5 mg/mL (*p*<0.01), 25 mg/mL (*p*<0.001) and 50 mg/mL (*p*<0.01). In addition, the levels of IL-1beta (*p*<0.001), IL-4 (<0.001), IL-5 (*p*<0.001), IL-6 (*p*<0.001), CXCL-1 (*p*<0.001), IL-13 (*p*<0.001), IL-17 (*p*<0.001), IL-23 (*p*<0.001), IL-33 (*p*<0.05) and TNF-alpha (p<0.001) were reduced in HDM+Exercise mice, while the levels of IL-2 (p<0.05) and IL-10 (p<0.001) were increased in BAL. AE also reduced HDM-induced IGF-1 (*p*<0.01) and VEGF (*p*<0.01) levels in lung homogenates. Furthermore, in re-stimulated mediastinal lymphnodes, splenocytes and in bone marrow cells, AE reduced the production of IL-4 (*p*<0.001), IL-5 (*p*<0.001) and IL-13 (*p*<0.001).

**Conclusions**

Aerobic exercise reduces asthma phenotype involving reduction of P2X7 receptor expression and reduction of Th2 cytokines production by lymphoid organs.

## A92 Nasal epithelium injury by chlorination products and other stressors predicts persistent sensitization to aeroallergens in young school children

### Alfred Bernard, Antonia Sardella, Catherine Voisin

#### Catholic University of Louvain Toxicology, Brussels, Belgium

##### **Correspondence:** Alfred Bernard

**Background**

Allergic sensitization during childhood is a highly dynamic process with a substantial rate of remission. Host or environmental factors influencing this process are largely unknown.

**Objective**

We tested the hypothesis of a link between aeroallergen sensitization and airway barrier dysfunction using epithelial biomarkers in nasal lavage fluid (NALF).

**Methods**

We conducted a two-year prospective study among 121 school children (mean age, 5.8 years; 64 boys). We measured club cell protein (CC16), β2-microglobulin (β2-m) and albumin in NALF and specific IgE to cat dander, pollen or house dust mite (HDM) in nasal mucosa.

**Results**

At follow-up, there were 26 children with new-onset, 24 with remitted and 16 with persistent sensitization to any aeroallergen. Odds of persistent sensitization to any aeroallergen increased across baseline ascending tertiles of urea-adjusted β2-m and albumin and descending tertiles of albumin-or β2-m-adjusted CC16 (P for trend = 0.006, 0.02, 0.044 and 0.006, respectively). Persistent sensitization to HDM also increased across baseline descending tertiles of raw or urea-adjusted CC16 (both P for trend = 0.007). Such associations were not seen with the new-onset or remitted aeroallergen sensitization or with the raw NALF concentrations of urea, albumin or β2-m. House cleaning with chlorine bleach and regular attendance of chlorinated pools emerged as the strongest and most consistent predictors of NALF biomarkers at baseline, being both associated with higher urea and lower CC16 in NALF.

**Conclusions**

Early childhood exposure to chlorination products at home or in swimming pools causes nasal epithelial barrier alterations predicting persistent aeroallergen sensitization.

## A93 Alterations in barrier integrity and inflammatory infiltrate are present and largely reversed with treatment in eosinophilic esophagitis

### Simon Royce^1^, Hamish Philpott^2^, Sanjay Nandurkar^3^, Francis Thien^4^, Peter Gibson^2^

#### ^1^Monash University Medicine, CCS, Melbourne, Australia; ^2^Monash University Gastroenterology, CCS, Melbourne, Australia; ^3^Monash University Gastroenterology, ECS, Melbourne, Australia; ^4^Monash University Respiratory Medicine, Melbourne, Australia

##### **Correspondence:** Simon Royce

**Background**

Loss of epithelial barrier integrity plays an important role in the process of allergic sensitization, as well as influencing subsequent pathological processes including local inflammation and fibrosis.

**Objective**

Our aim was characterize the inflammatory infiltrate (including IgG and IgE deposition) and changes in barrier integrity of patients with eosinophilic esophagitis (EoE) treated with a range of modalities, and normal controls.

**Methods**

Immunohistochemical analyses were performed on biopsies from 20 (>18y old) patients with EoE and then given 6wk dietary therapy with proton pump inhibition (diet-PPI), 18 with PPI-responsive esophageal eosinophilia (PPI-REE), 10 managed with Budesonide monotherapy (Bud) and 10 normal controls were analysed (recruited from the Box Hill and The Alfred Hospitals, Melbourne, Australia).

**Results**

Patients with EoE had a higher mean eosinophil count in all esophageal regions and the intensity of staining of mast cell tryptase was also increased except in the mid-esophagus. Expression of IgG and IgE was increased in all esophageal regions (*p*=<.05). The expression of caveolin was lower throughout the esophagus (*p*=<.05), but desmoglein was only reduced in the lower esophagus. The mean eosinophil count fell markedly in the patients treated successfully (diet-PPI, Bud, PPI-REE) in all esophageal regions (*p*<.05). Mast cell tryptase staining intensity decreased with the three treatments (*p* <.05) (limited to lower esophagus in Bud, PPI-REE). Tissue IgG fell in response to diet-PPI in the lower esophagus only (*p*<.05) but tissue IgE did not change significantly with any of the treatments. The expression of both measures of epithelial barrier integrity, caveolin and desmoglein, increased significantly with all treatments (*p* <.05 all areas, apart from diet-PPI, limited to the lower and middle esophagus). As per the definition of a positive food antigen challenge, food antigen reintroduction after the treatment period increased eosinophil and mast cell tryptase, and decreased barrier markers (all *p*<.05) but did not alter IgG or IgE.

**Conclusions**

In conclusion, an inflammatory infiltrate typical of a Th-2 mediated disease process is present in patients with EoE and those with PPI-REE. Treatment with budesonide, diet and PPI (for EoE) or PPI alone (PPI-REE) all largely resolve the changes associated with eosinophilia. The inflammatory infiltrate and impairment in barrier integrity as determined by immunohistochemistry is produced by food antigen exposure. The lower esophagus appears particularly important in initiating inflammation, whilst density of IgG and IgE deposition appear to relate to the duration of the inflammatory response.

## A94 AllergoOncology: the immune microenvironment, in allergy and cancer, is critically shaped by macrophages not only in humans, but also in dogs

### Rodolfo Bianchini^1^, Franziska Roth-Walter^1^, Anna Ohradanova-Repic^2^, Gerlinde Hofstetter^1^, Ina Herrmann^3^, Maria Isabel Carvalho^4^, Karin Hufnagl^1^, Erika Bajna^5^, Georg Roth^6^, Hannes Stockinger^2^, Erika Jensen-Jarolim^5^

#### ^1^The Interuniversity Messerli Research Institute; University of Veterinary Medicine; Medical University of Vienna; University of Vienna Department of Comparative Medicine, Vienna, Austria; ^2^Center for Pathophysiology, Infectiology and Immunology, Medical University of Vienna Institute for Hygiene and Applied Immunology, Vienna, Austria; ^3^University of Veterinary Medicine Clinical Department of Small Animal Internal Medicine, Vienna, Austria; ^4^University of Tràs-os-Montes and Alto Douro Department of Veterinary Science, Vila Real, Portugal; ^5^Center of Pathophysiology, Infectiology and Immunology, Medical University Vienna Department of Pathophysiology and Allergy Research, Vienna, Austria; ^6^General Intensive Care and Pain Medicine, Medical University Vienna Department of Anesthesiology, Vienna, Austria

##### **Correspondence:** Erika Jensen-Jarolim

**Background**

Almost 10 million people in the world die of cancer, and about 50 million suffer from IgE-mediated allergies every year. Likewise, cancer is a leading death cause in companion animals, - and they can also develop allergies. Whereas IgE immunoglobulins are pathognomic in allergies, the IgG4 class is typical in allergies that have been cured, and it has only recently been correlated with bad prognosis in cancer. In both diseases the immune system is thus critically involved: Whereas in allergies it is overreactive against harmless antigens, it is hyporeactive in cancer through immune tolerance.

**Methods**

In this project we concentrate on the immunoregulatory function of human and animal macrophages which in concert with immunoglobulins have a great plasticity of response. The in vitro polarization of macrophages into functional subtypes M1, M2a, -b or -c has been established for human and - for the first time - canine cell lines, as well as peripheral blood mononuclear cells from donors, allergic patients or cancer patients.

**Results**

When we investigated the immune cells surrounding the tumour mass in human colon cancer specimens, we found that macrophages were present not only at a highly density, but also in the vicinity of IgG4 expressing cells. When we investigated in vitro the capacity of macrophages to interact with IgG of different subclasses we found that only IgG4, but not IgG1 drives macrophages into a non-responsive tolerogenic phenotype. IgG4 also led to an expression of several cytokines and chemokines such as IL-10, IL-6, TNFa, or CCL1, and converted and reinforced the M2b-like tolerogenic macrophages.

**Conclusions**

Our results highlight the similarities between human and animal macrophage responses in cancer and allergy. The in vitro data based on cell lines and on blood cells from allergic & cancer patients will contribute to the understanding of the mechanisms and improve treatment options and prognosis in animals and humans.

## A95 Maternal evaluation knowledge on Epinephrine Auto-injector usage in Israel

### Meital Almog^1^, Aharon Kessel^1^, Larisa Apov^2^

#### ^1^Bnai Zion Medical Center Division of Allergy & Clinical Immunology, Haifa, Israel; ^2^Bnai Zion Medical Center Division of Allergy & Clinical Immunology, Haifa, Israel

##### **Correspondence:** Meital Almog

**Background**

Worldwide, there has been an increase of potentially life-threatening food allergy events. In Israeli children, anaphylaxis occurs most frequently after ingestion of milk, sesame seeds, peanuts and tree nuts. Correct use of the Epinephrine auto-injector is essential in order to prevent death from anaphylaxis.

**Objective**

The aim of this study was to explore the knowledge and factors that influence caregiver’s use of the Epinephrine among children with food allergy.

**Methods**

The study population comprised 128 children up to 18 years of age with known food allergy. Each caregiver was interviewed by a trained physician who then completed a computer-based questionnaire including parameter as demographic attributes, the caregiver’s years of education, date of their last visit to the allergy clinic, a previous anaphylactic event. The clinical indications and the operation of the Epinephrine auto-injector as recommended by the Israeli Association of Allergy and Clinical Immunology were also assessed and scored.

**Results**

One hundred twenty-eight maternal care givers and 52 paternal care givers participated in the study. The maternal caregiver Epinephrine auto-injector usage and indication competency scores were significantly higher than the paternal caregiver scores, (69±30.7 versus 56.57±35.55, *p*=0.01, 86.15±16.03 versus 80±17.54, *p*=0.03, respectively). Forty-two percent of the maternal caregivers demonstrated proficiency of the Epinephrine manual usage (3 out of 3 correct answers), 20% had a functional Epinephrine using (2 out of 3 essential parameters to operate the Epinephrine device), and 37.5% of the maternal care givers had a minimal knowledge which didn’t allow them to operate the Epinephrine device functionally. A statistically negative correlation was found between the maternal caregivers Epinephrine usage score and the date of their last visit at the allergy clinic (r=-0.66, *p*≤0.01). The mothers of those children who experienced an anaphylactic event in the past had a significantly higher Epinephrine usage score compared to mothers of children who didn’t experienced such an event (15/26 vs. 28/78, *p*= 0.04). No correlation was found between the maternal Epinephrine usage score to maternal education, age of the child, type of food allergen. Moreover, no correlation was found between maternal caregivers indication score and the date of their last visit to the allergy clinic.

**Conclusions**

The optimal use of Epinephrine auto-injector is related to the time that elapsed from a patient’s last allergy clinic visit. Periodic refreshing training, every 4-6 months, for the caregiver, on the indications and manual use of the Epinephrine device should be undertaken to ameliorate food allergy anaphylaxis management.

## A96 Omalizumab in desensitization food techniques

### Carlos Sanchez Salguero, Alvaro Sanchez Chacon

#### University Hospital Puerto Real Pediatric, Puerto Real (Cadiz), Spain

##### **Correspondence:** Carlos Sanchez Salguero

**Background**

According to the Alergologica 2005 study, milk and egg are the foods most often implicated in the diagnosis of allergy in patients under 5 years of age. The estimated prevalence of CMPA in the first year of life is 1.6-3%.

Until a few years, the only treatment accepted for this patients were eliminated these food of the diet, but actually we have a new therapeutical way, the Specific Oral Tolerance Induction (SOTI).

**Methods**

During the period 2010-2016, we had treated with SOTI method a total of 70 childs: 52 with cow’s milk allergy (CMA) and 18 with egg allergy (EgA).

The SOTI protocol in CMA: a first phase with a dilution of 1 ml of cow’s milk with 99 ml of juice, the patient drunk each 60 min 1-2-4-8 ml, after a new dilution 1/10 with the dosage of 1,6-3,2-6-12 ml and finally the first day we finished with 2,5 ml of whole milk (total 4,8 ml). After, each week, we increased the dosage of whole milk: 10-20-40-80-140-250 ml.

Group of CMA the values of the casein proteins were 3,5-500 IU/ml and in this group we had a subgroup of 12 childs they were presented anaphylaxis. The SOTI protocol in EgA: capsules with doses of ovomucoid: 4-20-50-100-225-450-900-1800-3600 mg. The first six doses are capsule with the same dose per day during each week, the last three doses each 3 days and finished with an oral provocation with a omelette.

Group of EgA the values of ovomucoid were 5-100 IU/ml, and also a subgroup of 5 childs with anapylaxis. All the patient with anaphylaxis were treated with Omalizumab 300 mg per month, three months before the new desensitization, during all the time that lasted the second desensitization and three months after finished the SOTI protocol.

**Results**

All patients underwent treatment with Omalizumab got both milk and egg desensitization protocol end to end and now tolerate 250 ml of milk a day and 3 eggs a week respective form.

**Conclusion**

Omalizumab has been useful introducing oral immunotherapy in food-allergic patients. A pilot study in 11 children with cow’s milk allergy showed the benefit of pretreatment with omalizumab from 9 weeks before and during a combined two phases. In summary, we demonstrated that omalizumab treatment combined with oral milk and egg desensitization in children with clinical anaphylactic reactions permitted rapid milk and egg dose escalation in the majority of subjects.

## A97 Climatic conditions, food allergies

### Abbos Nazarov^1^, Shaxbos Ergashev^2^

#### ^1^Tashkent Medical Academy Allergology, Tashkent, Uzbekistan; ^2^Medstar Biology, Qarshi, Uzbekistan

##### **Correspondence:** Abbos Nazarov

**Background**

Currently, food allergy, that is, cooking complicated and widespread intolerance of separate parameters sets. The development of allergies in the present system of food products, artificial additives, plant cultivation and storage of chemicals used in a variety of different types of drinking lead to the emergence of new allergens.

**Objective**

Patients to identify the types of food allergy and its meetings in accordance with the climatic conditions

**Methods**

Nutrition 60% of patients with diseases of the emergence of allergies, allergic history, 20% of allergy symptoms and the presence of genetic susceptibility to other members of a special kind of plays and plays, especially in patients with allergic rhinitis, seasonal or permanent type observed. In this way, members of diseases of the digestive diseases, lack of digestive enzymes intestinal disbacteriosis lead to unhealthy lifestyle and diet. RIIAM monitoring patients for surveillance and investigation results showed that Nutriv 25% of the cases, the immune mechanisms associated with allergies. 10% of patients with disease on the basis of immediate type allergic reactions often cause blisters and angioedema from 13% to 45% of the cases. Nutriv allergy is more often associated with skin changes, 60% of the erythematous vesicular dermatitis, food allergy can cause bronchial asthma adults, 5% of patients, and other allergens as well as 22% of patients with 10% of the cases, other kinds of toxicodermia.

**Results**

According to allergic patients with a history of food to 60% of honey, walnuts 50%, 70% and 30% of the meat and milk of various colored drinks, 18% vegetables, 25% fruits, 35% quziqorin, 33% of eggs, 12% of fish products 3% of allergic reactions observed grasses.

**Conclusions**

Food allergy prone patients that must be hypoallergenic diet.

## A98 The efficiency of the immunomodulatory therapy in treatment of immunocompromised girls with recurrent chronic non-specific vulvovaginitis associated with atopic dermatitis

### Irina Nesterova, Svetlana Kovaleva, Galina Chudilova, Ludmila Lomtatidze

#### The Peoples’ Friendship University of Russia, Department of Allergology and Immunology, Moscow, Russia

##### **Correspondence:** Irina Nesterova

**Background**

The treatment of recurrent chronic non-specific vulvovaginitis (CNSV) associated with atopic dermatitis (AD) in girls in age from 2 to 4 years is very difficult problem because methods of the traditional therapy are often ineffective. The development of new approaches for the treatment of these patients are the actual problem.

**Objective**

We had conducted the clinical and immunological study of 25 girls in age from 2 to 4 years, who were at the acute stage of recurrent CNSV. Ten of these girls had AD and clinical features of immunodeficiency (ID): recurrent acute respiratory viral infections (ARVI) - more than 8 times per year and frequent exacerbations of CNSV.

**Methods**

Different subpopulation of T-, B-lymphocytes, EKK, phagocytic, microbicidal functions of neutrophils (NG, the serum IgE, IgA, IgM, IgG, were detected. The control group consisted of 12 healthy girls.

**Results**

It was found, that at the acute stage of recurrent CNSV and AD in ID’ girls the decrease of the absolute number of CD3+CD8+ (0,9 [0,83; 1,02] vs. 1,38 [0,91; 1,63] in the control, *p*<0,05), CD3-CD16+CD56+ (0,33 [0,24; 0,37] vs. 0,43 [0,31; 0,48] in the control, (*p*<0,05), CD3-CD19+ (0,62 [0,52; 0,71] vs. 1,01 [0,95; 1,24] in the control, p<0,05) lymphocytes, deficiency of serum IgA (*p*<0,05), IgG (*p*<0,05) and IgM (*p*<0,05), defects of phagocytic (44,86 [39; 51]% vs. 61,08 [53,8; 68,8]% in the control, p<0,05) and microbicidal activities of NG (*p*<0,05) had occurred. It was revealed that 63% of girls suffered from IgE independent, and 37% from IgE dependent food allergy with clinical manifestations of atopic dermatitis. The level of IgE amounted 112,51 [96,4; 172,2] IU/ml against 26,83[10,4; 38,3] IU/ml in control (*p*<0,05). The program of the prolonging combine immunotherapy (PPCI) was created and was used in the complex of the traditional therapy. The PPCI included local (gel) and systemic (suppositories) treatment with recombinant interferon α2b (viferon) and glucosaminilmuramildipeptide (likopid). It was obtained positive clinical effects using PPCI: the severity of the inflammatory process and the frequency, the duration of exacerbations of CNSV was decreased on 3-5 days and the significant reduction of frequency of ARVI and the regression of manifestations of the atopic dermatitis was obtained.

**Conclusions**

The peculiarities of the immunopathogenesis of CNSV and AD in ID girls are causes of persistent recurrence of CNSV and it resistance to the tradinional therapy.The use of PPCI had demonstrated the good protective and positive clinical effect in ID girls with recurrent CNSV, AD.

## A99 START: Susceptibility to food allergies in a registry of twins

### Sarah De Schryver^1^, Alizee Dery^2^, Ann Clarke^3^, Kari Nadeau^4^, Laurie Harada^5^, Kimberly Weatherall^6^, Celia Greenwood^7^, Denise Daley^8^, Yuka Asai^9^, Moshe Ben-Shoshan^1^

#### ^1^McGill University Pediatric Allergy and Immunology, Montreal, Canada; ^2^McGill University Clinical Epidemiology, Montreal, Canada; ^3^University of Calgary Rheumatology, Calgary, Canada; ^4^Stanford University Pediatric Allergy, Immunology and Rheumatology, Palo Alto, CA, USA; ^5^Anaphylaxis Canada, Toronto, Canada; ^6^Multiple Births Canada, Lefroy, Canada; ^7^McGill University Oncology, Epidemiology, Biostatistics, Occupational Health and Human Genetics, Montreal, Canada; ^8^University of British Columbia Medicine, Vancouver, Canada; ^9^Queen’s University Dermatology, Kingston, Canada

##### **Correspondence:** Moshe Ben-Shoshan

**Background**

To compare proband-concordance [(twice number concordant pairs)/(twice number of concordant pairs+ number of non-concordant pairs)] in MZ (monozygotic) and DZ (dizygotic) twins with food allergy.

**Methods**

Twins, with at least one member with IgE-mediated food allergy, were recruited from Anaphylaxis Canada, Multiple-Births-Canada, a US registry and the Montreal Children’s Hospital allergy clinics. Diagnosis of food allergy was based on a convincing clinical history AND a skin prick test or allergen-specific IgE level beyond previously published thresholds or a positive food challenge.

**Results**

Among 17 pairs of DZ and 15 pairs of MZ twins with peanut allergy, the concordance rate was 0.59 (95% CI 0.39, 0.77) and 0.52 (95% CI, 0.29, 0.75) respectively [difference=0.07 (95% CI 0.06, 0.40)].

Among 9 pairs of DZ twins and 12 pairs of MZ twins with cashew/pistachio allergy, the concordance-rate was 0.57 (95% CI 0.29, 0.81) and 0.50 (95% CI 0.27, 0.72) respectively [difference=0.07 (95% CI 0.02, 0.75)].

Among 4 pairs of DZ and 9 pairs of MZ twins with pecan/walnut allergy, the concordance rate was 0.57 (95% CI, 0.20, 0.89) and 0.54 (95% CI 0.25, 0.82) respectively [difference=0.03 (95% CI, -0.47, 0.52].

Among 6 pairs of DZ and 8 pairs of MZ twins with hazelnut allergy the concordance-rate was 0.33 (95% CI 0.06, 0.76) and 0.2 (95% CI 0.03, 0.55) respectively [difference=0.13 (95% CI -0.45, 0.72)].

Among 4 pairs of DZ and 5 pairs of MZ twins with almond allergy the concordance rate was 0.5 (95% CI 0.15, 0.84) and 0.33 (95% CI 0.05, 0.75) respectively [difference=0.17 (95% CI -0.62, 0.95)].

Among 10 pairs of DZ and 8 pairs of MZ twins with egg allergy the concordance-rate was 0.53 (95% CI 0.27, 0.77) and 0.22 (95% CI 0.03, 0.59) respectively [difference= 0.31 (95% CI 0.14, 0.77)].

**Conclusions**

For peanut, tree-nuts and egg allergy, the concordance among DZ twins was higher compared to MZ twins. For other foods assessed such as milk and shellfish, the difference was not conclusive. It is likely that non-genetic factors play a major role in the development of food allergy. However, our small sample size precludes definitive conclusions.

## A100 HIV and HCV co-infection: the order of HIV and HCV acquisition and liver fibrosis

### Irina Balmasova, Elena Malova

#### Peoples’ Friendship University of Russia Allergology & Immunology, Moscow, Russia

##### **Correspondence:** Irina Balmasova

**Background**

Coinfection with human immunodeficiency virus (HIV) and hepatitis C virus (HCV) is one of the most common pathological conditions worldwide. Liver disease is a major cause of death for these patients. We attempted to determine whether the order of HIV and HCV acquisition is important for the progression of liver fibrosis.

**Objective**

Four hundred twenty-five patients with verified HIV/HCV coinfection were followed for 1 year. The order of HIV and HCV acquisition was identified in 106 patients: in 61 patients HCV was acquired after established HIV infection (i.e., the first pathogen was HIV) while in 45 patients HIV was acquired after established HCV infection (i.e., the first pathogen was HCV).

**Methods**

The patients underwent transient elastometry to measure liver stiffness, viral load monitoring was performed by real-time PCR, CD3+CD4+ cell count was measured by flow cytometry. SPSS was used for statistical analyses.

**Results**

Among 106 patients with HIV/HCV coinfection, progressive liver fibrosis was diagnosed in 21%, stable liver fibrosis in 48%, regressive liver fibrosis in 31%. It was found that the order of HIV and HCV acquisition has a significant effect on the development of progressive liver fibrosis. It was demonstrated that, when HIV was the first pathogen, progressive liver fibrosis occurred in 27% of patients. When HCV was the first pathogen, progressive liver fibrosis occurred in 13% of patients only. the highest (30%) prevalence of progressive fibrosis was observed in patients younger than 35 who acquired HIV as the first pathogen. In high-risk patients, HIV viral load was significantly higher both by the prevalence and absolute value as compared with minimum-risk patients. HCV viral load demonstrated no significant difference between the groups. CD3+CD4+ cell count in the high-risk group was less than in the control group.

**Conclusions**

The order of HIV and HCV acquisition has a significant effect on the progression of liver fibrosis in co-infected patients. In was shown that in patients younger than 35, the order of HIV and HCV acquisition has a crucial role in this process. In particular, the patients who acquired HIV as the first pathogen are at higher risk of progressive liver fibrosis and have higher HIV viral load and lower CD3+CD4+ cell count.

## A101 The hyper-allergenic isoform Bet v 1a of the major birch pollen allergen displays reduced allergenic potential after binding of retinoic acid

### Karin Hufnagl^1^, Stefanie Wagner^1^, Luis F. Pacios^2^, Michael Wallner^3^, Markus Wiederstein^3^, Gerlinde Hofstetter^1^, Franziska Roth-Walter^1^, Erika Jensen-Jarolim^4^

#### ^1^The interuniversity Messerli Research Institute of the University of Veterinary Medicine, the Medical University and the University of Vienna Comparative Medicine, Vienna, Austria; ^2^ETSI Montes, Technical University of Madrid Department of Natural Systems and Resources, Madrid, Spain; ^3^University of Salzburg Department of Molecular Biology, Salzburg, Austria; ^4^Center of Pathophysiology, Infectiology and Immunology, Medical University Vienna Department of Pathophysiology and Allergy Research, Vienna, Austria

##### **Correspondence:** Erika Jensen-Jarolim

**Background**

Bet v 1a (Bet v 1.0101) represents the most typical allergen prototype. Its isoform Bet v 1d (Bet v 1.0102), however, is a hypoallergen despite of being structurally highly homologous. We hypothesized that the ligand-binding capacity of these lipocalin-like molecules could discriminate the level of allergenicity.

**Objective**

We investigated thus whether the active vitamin A metabolite retinoic acid (RA) can bind to Bet v 1a or Bet v 1d, and how this affects their allergenicity in vitro.

**Methods**

Binding of RA to Bet v 1 isoforms was determined by in silico docking analyses and ANS displacement assay. We measured antigen-specific IgE by ELISA and ß-hexosaminidase release from humanized rat basophil leukemia cells (RBL) in sera from birch pollen allergics using the “empty” birch pollen allergens (apo-Bet v 1a/d) or Bet v 1 isoforms loaded with RA (holo-Bet v 1a/d).

**Results**

In silico calculations predicted a high affinity energy (-8.7 kcal/mol) of RA to both Bet v 1a and Bet v 1d, with an estimated dissociation constant (KD) of 0.364 μM. In vitro RA was able to dose-dependently displace ANS from both isoforms to a similar extent. In ELISA holo-Bet v 1a loaded with RA showed significantly reduced binding of serum IgE compared to the empty apo-Bet v 1a. In agreement, holo-Bet v 1a produced significantly less mediator release from RBL cells. In contrast, both apo- and holo- Bet v 1d displayed much lower IgE binding capacity and preliminary data indicate that RA loading of Bet v 1d has no effect on RBL releasability.

**Conclusions**

RA loaded into the intramolecular pocket of Bet v 1a reduces its IgE-binding and IgE-crosslinking potential in vitro. Our data may have implications for improving the safety of birch pollen allergen immunotherapy.

This work was supported by grant SFB F4606-B28 of the Austrian Science Fund FWF.

## A102 Effect of immunotherapy autological activated T-cells on the parameters of the immune status in patients with bronchial asthma

### Anna E. Tevs^1^, Ekaterina A. Pashkina^2^, Marina I. Leonova^1^, Vera M. Nepomnyaschikh^1^, Darya V. Demina^1^, Vladimir A. Kozlov^2^, Elena A. Blinova^2^

#### ^1^Clinic of Immunopathology RIFCI Allergological Department, Novosibirsk, Russia; ^2^RIFCI Laboratory of Clinical Immunopathology, Novosibirsk, Russia

##### **Correspondence:** Anna E. Tevs

**Background**

Lack of effectiveness of standard treatment protocols for patients with bronchial asthma served as the basis for the search new directions of systemic therapy of diseases affecting the pathogenetic links. One of such area is a cellular immunotherapy aimed to activation anti-ergotypic response realized in response to activated T cells, for regulation of allergic inflammation.

**Methods**

The study included 10 patients with asthma, atopic and mixed etiology, moderate severity, and aged 23 to 49 years. Patients were treated at the allergological department on Clinic immunopathology RIFCI, with a basic therapy during the 12 weeks prior to entry to the study. In the group, total IgE level estimated 1115 IU / ml, the rate of FEV1 - 69%. After obtaining informed consent, patients were administered subcutaneously autologous activated T-cells with multiplicity once a week 4 injections, and then once a month 6 injections, on the background of basic asthma therapy (10 injections in total). Preparation protocol for autologous activated T-lymphocytes included activation of mononuclear cell from peripheral blood by anti-CD3 antibodies (1mkg/ml) and IL-2 (100IU/ml). It was used 30 million cells per dose. Evaluation of the immune status was carried out in the dynamics of cellular immunotherapy (before and on 5th injection).

**Results**

There was a decrease of total IgE levels up to 257 IU/mL, as well as rising of FEV1 to 88%. Positive effects of T-cell immunotherapy can be expressed in enhancing natural regulation mechanisms on activated T-cells: reducing cell proliferation, changing the products of Th1 and Th2 cytokines and immunoglobulins, especially IgE. On the 5th injection, patients showed a slight increase in the number of CD8+ T-cells (27% to 35%), the number of CD19+ B-cells decreased from 14% to 10%. Anti-ergotypic effectors are cytotoxic lymphocytes (CD8), thus increasing their level during immunotherapy indicates the induction of anti-ergotypic response. It is known that Th1-type cytokines act on B-cells, leading to reduction in the number of activated CD23+ B-lymphocytes with low affinity receptor IgE (FceRII). Therefore, reducing the number of CD19+ B cells in the process of T-cell vaccination may occur by reducing the number of CD23+ B-lymphocytes. There was no effect of immunotherapy by activated T cells on the amount of CD4+ and NK-cells.

**Conclusions**

Thus, in the course of immunotherapy with autologous activated T cells in asthmatic patients there was an increase of anti-ergotypic effector cells that clinically manifested in the reduction of total serum IgE and improving respiratory function.

## A103 Virus-associated allergic rhinitis as a phenotype and its clinic-immunological specifics

### Nataly Tataurshchikova, Baigalmaa Sangidorj, Anna Ronzhina

#### Peoples Allergology and Immunology, Moscow, Russia

##### **Correspondence:** Nataly Tataurshchikova

**Background**

Persistent prevalence increase in torpid to traditional treatment schemes forms of allergic rhinitis and worsening clinical symptomatics during the last years is connected to specifics of mucosal immunity functioning among patients. Mucosal barrier functioning status among patients, suffering from allergic rhinitis, whose comorbid status is herpetic-viral infection, are the basis of clinical phenotype – virus-associated allergic rhinitis. Nasal mucosal among patients with herpetic-viral infection loses immunological tolerance to allergens, infectious pathogens, thus becoming a permanent palindromic inflammation process area.

**Methods**

During an open prospective research a clinic-immunological survey was conducted among 63 patients (male and female both) aged 18 to 53, suffering from perennial allergic rhinitis for 3 to 10 years. All the patients received and standard basic therapy according to indications. Glucocorticosteroids were excluded 1 month before survey start. 2 groups of patients were formed as a result of clinic-immunological survey – main group with perennial virus-associated allergic rhinitis (HSV infections and/or CMV infections) - 42 patients and control group - 21 patient with perennial allergic rhinitis. Alongside with standard clinic-immunological survey a key specific cytokine content analysis was made using immunoenzymic method (proinflammatory - (α-TNF – tumor necrosis factor, IL-8, γ – INF) and antiphlogistic IL-4) of serum and local fractures. Material blood serum, taken fasting in the morning from ulnar vein and nasal lavage.

**Results**

Clinic-immunological changes analysis among group of patients, suffering from virus-associated allergic rhinitis showed the following pathognomonic evidence. Clinic specifics – frequent ARD (more than 8 times a year, chronic ENT-pathology, herpetic infection. Among 31 patient (73,8%) an increase of IL-8 content in nasal lavage was stated. Among 8 patients (19%) a sharp increase of α-TNF was stated, accompanied by high herpetic infection titles (HSV infection and/or CMV infection).

While investigating local cytokine status among patients, suffering from perennial allergic rhinitis both for main and control groups an increase in IL-4 level in nasal secret was stated, alongside with non-significant changes in blood serum. All the patients had multidirectional changes in γ interferone content for both serum and local fractures that shows a typical cytokine profile of an allergic rhinitis. No reliable distinctions were stated among groups.

**Conclusions**

Presence of herpetic-viral infection among patients, suffering from allergic rhinitis leads to nasal mucosal inflammatory process specifics and forms an allergic rhinitis clinical phenotype – virus-associated allergic rhinitis.

## A104 Clinico-immunological and aeropolinologic monitoring in patients with respiratory allergies

### Revaz Sepiashvili^1^, Tatiana Slavyanskaya^1^, Manana Chikhladze^2^

#### ^1^Peoples’ Friendship University of Russia Allergy and Immunology, Moscow, Russia; ^2^National Institute of Allergology, Asthma and Clinical Immunology of Georgian Academy of Sciences Immunology and Allergy, Tskhaltubo, Georgia

##### **Correspondence:** Revaz Sepiashvili

Clinico-epidemiologic study proves that the Western Georgia is characterized by frequency and variety of allergic diseases, especially respiratory allergies, determined by sharp features of eco-geographical climate. Due to the above-mentioned, at this stage the study aimed to establish the correlation between the concentrations of phadiatop, general IgE and specific aeropollutants, taking into consideration the annual calendar of plants blossoming for the reality of Imereti region.

In the study were involved 69 patients, 5-14 of ages, with allergic rhinitis and asthma (34 boys and 35 women)

The study covered the following allegro-diagnostic stages:I stage - For precise verification of the allergen, patient’s blood serum was examined on a particular specific-IgE antibodies by modern automated system - “ImmunoCAP 100” (PHADIA). II stage - Monitoring of the concentration of aeropoluments was conducted by using aeropolinometer “Burkard Trap” (Great Britain).

The analysis of the laboratory results revealed that the studied patients had high titers of common IgE, which amounted to 380kU/I (Norm 60-200kU/I) on average, while the concentration of the phadiatop was above average (norm - negative). In parallel, the data obtained through the aeropolinometris- “Burkard Trap” were specified in line with the annual calendar of distribution of aeroallergens, reflecting the concentrations of blossoming tree-plants and atmospheric aerosolsin air for the reality of Imereti region. From January to April, 2016, a high concentration of aeropollutants which are characterized with high allergenization and at the same time widely spread in this region, was revealed. It should be emphasized that especially high concentrations were detected in alder, birch-tree and nut-grove. Among the widely spread aeropollutants of low allergenization were distinguished poplar, elm, willow and plane trees. Every aforementioned diagnostic marker highly correlated with each other. Increased concentrations of Immuno CAP/Phadiatop in the blood as well as the high levels of IgE prove existence of atopic allergy to the inhaled allergens. Aeropollinometer “Burkard Trap” was used to detect he concentrations of various plant dust and surrounding atmospheric aeropollutants at a given period of time and to specify annual calendar of distribution of aeroallergens developed by us for the reality of Imereti region, respectively. The strong correlation between the above mentioned diagnostic markers indicates to their importance to the respiratory allergies.

## A105 The role of B cells in rhinovirus infection

### Oliver F. Wirz^1^, Willem van de Veen^1^, David Mirer^1^, Hideaki Morita^2^, Can Altunbulakli^1^, Sebastian L. Johnston^3^, Nicholas Glanville^3^, Nikolaos G. Papadopoulos^4^, Cezmi A. Akdis^1^, Mübeccel Akdis^1^

#### ^1^University of Zurich Swiss Institute of Allergy and Asthma Research (SIAF), Davos Platz, Switzerland; ^2^University of Tokyo National Research Institute for Child Health and Development, Tokyo, Japan; ^3^Imperial College London Airway Disease Infection Section, National Heart and Lung Institute, London, United Kingdom; ^4^University of Manchester Division of Infection, Immunity and Respiratory Medicine, Manchester, United Kingdom

##### **Correspondence:** Oliver F. Wirz

**Background**

Rhinoviruses (RV) play a major role in asthma exacerbations. Effects of RV on many cell types have already been described including epithelial cells and lymphocytes, such as CD4+ T cells. However, not much is known about the interaction of rhinovirus with B lymphocytes.

**Objective**

The aim of this study was to investigate the role of B cells during rhinovirus infection.

**Methods**

B cells were sorted using fluorescent-activated cell sorting. Viral RNA was measured in these cells using qPCR. Antibodies produced by B cells were measured using ELISA and multiplex assays.

**Results**

Peripheral blood mononuclear cells (PBMC) from non-allergic individuals were stimulated with RV16. A virus dose-related cellular proliferation was observed, which was particularly confined to B lymphocytes. Proliferation was prevented by UV-irradiation of the virus. Blocking the surface receptor for RV16 (ICAM-1) leads to reduced cell proliferation. Blocking the endosomal Toll-like receptors (TLR) 3, 7 and 8 reduced the proliferation and viability of the B cells. Using multiplex assays, we measured all antibody subclasses produced by B cells during rhinovirus stimulation. We identified a change in antibody production towards elevated IgM, when cells were stimulated with rhinovirus. Positive (+) and negative (-) strands of viral RNA of RV16 were detected by quantitative PCR. Virus load stayed constant in the B lymphocyte compartment over a period of seven days, while it decreased in PBMC.

**Conclusions**

This study shows for the first time that B cells can be infected with RV by using ICAM-1 and induce proliferation through TLRs 3,7, and 8. We found both (+) and (-) viral RNA strands, suggesting that there is not only RV inside or attached to the cells, but also that RV is replicating in B cells. RV can be detected inside the B cells already within one day after infection. Unlike PBMC, the viral load stays mostly constant over an extended period of cell culture in B cells. Therefore, the data suggest that B cells could be a natural reservoir of RV.

## A106 Randomized, double-blind, placebo-controlled trial of recombinant human C1 inhibitor for prophylaxis of hereditary angioedema attacks

### Avner Reshef^1^, Marc Riedl^2^, Vesna Grivcheva Panovska^3^, Dumitru Moldovan^4^, James Baker^5^, William H. Yang^6^, Sladjana Andrejevic^7^, Richard F. Lockey^8^, Roman Hakl^9^, Shmuel Kivity^10^, Luca Bellizzi^11^, Joseph R. Harper^12^, Anurag Relan^11^, Marco Cicardi^13^

#### ^1^University of Tel Aviv Sheba Medical Center, Tel-Hashomer, Israel; ^2^University of California, San Diego Department of Medicine, La Jolla, CA, United States; ^3^Medical University of Skopje PHU Clinic for Dermatology, Skopje, Macedonia; ^4^ Mures County Hospital University of Medicine and Pharmacy, Tirgu Mures, Romania; ^5^Baker Allergy Asthma Dermatology Departments of Allergy and Asthma, Lake Oswego, CA, United States; ^6^Ottawa Allergy Research Corporation/University of Ottawa Medical School Division of General Internal Medicine, Department of Medicine, Ottawa, Canada; ^7^ Clinical Center of Serbia Clinical Center, Belgrade, Serbia; ^8^University of South Florida Morsani College of Medicine Division of Allergy and Immunology, Department of Internal Medicine, Tampa, FL, United States; ^9^St. Anne’s University Medical Department, Brno, Czech Republic; ^10^The Tel Aviv Medical Center, Sackler Medical School The Allergy and Immunology Unit, Tel Aviv, Israel; ^11^Pharming Technologies BV Department of Clinical Research and Medical Affairs, Leiden, The Netherlands; ^12^Salix Pharmaceuticals Department of Medical Affairs, Raleigh, NC, United States; ^13^Universita degli Studi di Milano Department of Internal Medicine, Milan, Italy

##### **Correspondence:** Avner Reshef

**Background**

Recombinant human C1 inhibitor (rhC1INH) has been shown to effectively treat acute attacks of hereditary angioedema (HAE) and is currently approved as on-demand therapy in several countries. Additionally, prevention of HAE attacks by rhC1INH has been demonstrated in a previous open-label study.

**Objective**

To evaluate the efficacy and safety of rhC1INH as prophylaxis against acute HAE attacks in adolescents and adults with HAE and frequent attacks.

**Methods**

In this phase 2, double-blind, 3-period crossover study, patients (≥13 years of age) with functional C1INH levels <50% of normal and history of ≥4 HAE attacks during the preceding 3 months received 1 of 3 treatments in 3 periods: intravenous rhC1INH 50 IU/kg (max, 4200 IU) once weekly, twice weekly, and placebo. Each treatment was administered for 4 weeks, with a 1-week separation (wash-out) between treatments. All patients received each of the dosing regimens as part of the crossover design of the study. Symptoms of each HAE attack were recorded daily. The primary endpoint was the number of HAE attacks per 4-week treatment phase. The secondary endpoint was the percentage of patients who had a clinical response (≥50% reduction in number of attacks from treatment with placebo to treatment with rhC1INH). Safety assessments included reports of adverse events [AEs] and immunogenicity.

**Results**

Of the 35 patients enrolled, 32 (91.4%) were randomized to treatment and included in the intent-to-treat population (all randomized patients). Mean patient age (standard deviation) was 45.9 (14.5) years, and most patients were female (81.3%). The mean number of HAE attacks was significantly reduced with rhC1INH twice weekly (2.7 attacks, P < 0.0001) and once weekly (4.4 attacks, P = 0.0004) compared with placebo (7.2 attacks). The percentage of patients who had a ≥50% reduction in number of HAE attacks was greater with rhC1INH twice weekly (74.2%; 95% CI, 57-86) than rhC1INH once weekly (41.9%; 95% CI, 26-59). The most commonly reported AEs were headache (17.2% with twice weekly rhC1INH, 6.9% with once weekly rhC1INH, 0% with placebo), nasopharyngitis (0%, 10.3%, 7.1%), and anxiety (0% 6.9%, and 0%). No thrombotic or thromboembolic events, drug hypersensitivity or anaphylactic reactions, or neutralizing antibodies were observed, and no patients withdrew because of AEs.

**Conclusion**

rhC1INH provided significant and clinically relevant reductions in HAE attack frequency and was well-tolerated. Data support the continued development of rhC1INH for HAE prophylaxis.

